# Synthesis, Characterization and Photoactivation Studies on the Novel Pt(IV)-Based [Pt(OCOCH_3_)_3_(phterpy)] Complex

**DOI:** 10.3390/ijms24021106

**Published:** 2023-01-06

**Authors:** Giovanni Canil, Juan Gurruchaga-Pereda, Simona Braccini, Lorella Marchetti, Tiziana Funaioli, Fabio Marchetti, Alessandro Pratesi, Luca Salassa, Chiara Gabbiani

**Affiliations:** 1 Centro di Riferimento Oncologico di Aviano, IRCCS, Via Franco Gallini, 2, 33081 Aviano, Italy; 2 Donostia International Physics Center, Paseo Manuel de Lardizabal 4, 20018 Donostia, Spain; 3 CIC biomaGUNE, Paseo de Miramón 182, 20014 Donostia, Spain; 4 Department of Chemistry and Industrial Chemistry (DCCI), University of Pisa, Via Giuseppe Moruzzi 13, 56124 Pisa, Italy; 5 Ikerbasque, Basque Foundation for Science, 48011 Bilbao, Spain; 6 Polimero eta Material Aurreratuak: Fisika, Kimika eta Teknologia, Kimika Fakultatea, Euskal Herriko Unibertsitatea UPV/EHU, Paseo Manuel de Lardizabal 3, 20018 Donostia, Spain

**Keywords:** Pt(IV) compounds, photoactivation, anticancer compounds, metal-based drugs, flavin-catalyzed light-activation

## Abstract

Photoactivatable Pt(IV) prodrugs represent nowadays an intriguing class of potential metal-based drugs, endowed with more chemical inertness in their oxidized form and better selectivity for the target with respect to the clinically established Pt(II) compounds. In fact, they have the possibility to be reduced by light irradiation directly at the site of interest. For this reason, we synthesized a new Pt(IV) complex, [Pt(OCOCH_3_)_3_(4′-phenyl-2,2′:6′,2′′-terpyridine)][CF_3_SO_3_] (**1**), that is well soluble in aqueous medium and totally unreactive towards selected model biomolecules until its reduction. The highlight of this work is the rapid and efficient photoreduction of **1** with visible light (460 nm), which leads to its reactive Pt(II) analogue. This behavior was made possible by taking advantage of an efficient catalytic system based on flavin and NADH, which is naturally present in the cellular environment. As a comparison, the reduction of **1** was also studied with simple UV irradiation, but both UV-Vis spectrophotometry and ^1^H-NMR spectrometry showed that the flavin-catalyzed reduction with visible light was faster. Lastly, the reactivity against two representative biological targets, i.e., human serum albumin and one monofilament oligonucleotide fragment, was evaluated by high-resolution mass spectrometry. The results clearly pointed out that the prodrug **1** did not interact with these targets until its photoreduction to the Pt(II) analogue.

## 1. Introduction

Cancer chemotherapy relies strongly on conventional Pt(II) drugs, i.e., cisplatin, carboplatin and oxaliplatin, which are used in almost 50% of treatments [1] because they have proven to be active in several solid tumors [2]. However, well-known severe side effects limit their application and may cause patients to drop the chemotherapy regimen. This downside is usually associated with the extensive off-target reactivity of Pt(II) compounds inside the body; indeed, after being administered intravenously, the drugs can bind to serum proteins and small molecules they find on their path before reaching the tumor site, leading to diffuse toxicity [3,4]. One of the most abundant proteins in the blood and a major target for metal-based compounds is human serum albumin (HSA) [3,5,6], but basically any suitable donor ligand can bind to cisplatin and analogues [7].

Nowadays, many research groups working in medicinal chemistry have shifted their research interest from Pt(II) to Pt(IV) anticancer drugs [8,9,10] because they are more inert and, therefore, less toxic. Pt(IV) compounds are considered prodrugs that have to be reduced to Pt(II) to exert their cytotoxic activity through the “activation by reduction” mechanism [11,12]. As a consequence, Pt(IV) compounds should remain inactive when injected into the bloodstream and not bind to biological targets. However, it has been shown that Satraplatin, one of the most promising Pt(IV) prodrugs, was reduced quite rapidly in the bloodstream and was eventually discarded during the phase III clinical trial since it did not show a convincing benefit in terms of overall survival with respect to cisplatin and carboplatin [13,14].

In this context, the resistance to reduction of Pt(IV) complexes could be considered beneficial in order to limit their toxicity, but we have to keep in mind that these drugs have to be reduced at a certain point to exert their action on the cellular DNA, which leads to the cancer cell death. The optimum level of stability that reduces the toxicity without losing anticancer activity might be very difficult to obtain. Another aspect that complicates even further the picture and limits possible predictions is the fact that reduction potentials measured with electrochemical methods do not always match with the reduction rates of the Pt(IV) complexes, as reported by Zhang [15]. This may be due to the possibility that some axial ligands form bridges with the reducing agents, facilitating the reduction. In fact, as they point out in their investigation, chlorides and hydroxides facilitate the reduction process, whereas carboxylates do not [15].

Another possible strategy for the activation of Pt(IV) prodrugs exploits the photosensitivity of Pt(IV) complexes; during the last two decades, Bednarski, Sadler and coworkers pioneered the use of light to induce the Pt(IV) to Pt(II) reduction [16,17,18,19,20], obtaining remarkable results in the field of photoactivated chemotherapy. In the light of these new and exciting findings, our group joined the challenge of synthesizing photoactivatable Pt(IV) prodrugs. Our design focused on a tridentate terpyridine-like (4′-phenyl-2,2′:6′,2′′-terpyridine, from now on called phterpy) ligand to improve the light absorption process. Even though the complexes proved to be sufficiently photoactivated, UV radiation was necessary to induce the reduction to Pt(II) [21]. Indeed, for the most part, the photoactivatable Pt-containing compounds that appeared in the literature require the use of UV radiation and high light doses to complete the photoreduction [22]. However, UV radiation should be avoided because light penetration in tissues drops dramatically, going from longer (red) to shorter (UVA) wavelengths [23]. In addition, a fast and efficient photoreduction process is vital in order to reduce the side effects that a prolonged light treatment could potentially cause in patients [24]. These issues definitely hamper the progression of this class of compounds towards preclinical studies.

Therefore, the aim of this work is to develop and study a fast photoreduction strategy on the aforementioned terpyridine Pt(IV) substrate, employing visible light irradiation.

Recently, the group of Salassa discovered a novel approach for the activation of Pt(IV) anticancer prodrugs with longer wavelengths and extremely low light doses. Exploiting a new type of bioorthogonal catalytic reactions, they were able to photocatalyze the reduction on Pt(IV) using flavin cofactors or flavoproteins and an electron donor, i.e., NADH and MES (2-(N-morpholino)ethanesulfonic acid) [25,26,27,28,29]. In their approach, flavins efficiently oxidize electron donors under light excitation at 460 nm, generating the flavin hydroquinone form that is crucial in the catalytic conversion of the Pt(IV) compound into biologically active Pt(II) analogues [30]. The electron donor (NADH or MES buffer) provides the electrons necessary for the reduction, thus preventing the self-decomposition of the flavin, allowing the reaction to proceed as highly efficient catalytic cycle [28].

Herein, we successfully apply this new activation strategy to a novel Pt(IV) complex. Its structure recalls the terpyridine-Pt(IV) metal core we described in a recent work [21]; however, our aim was to obtain a compound that was more stable toward unwanted reduction reactions and more water-soluble—features that are crucial for any potential Pt(IV) compound to be clinically interesting.

## 2. Results and Discussion

### 2.1. Synthesis and Characterization

**Scheme 1:**
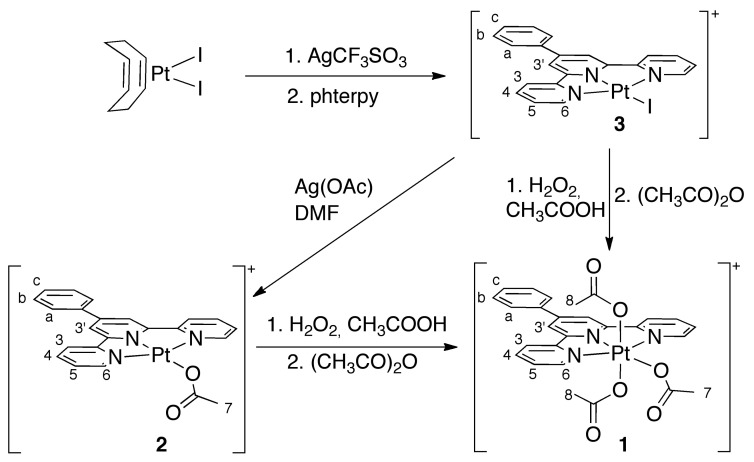
Highlights on the synthesis route of **3**, **2** and **1**. The CF_3_SO_3_^−^ counterion has been omitted for clarity.

In very recent years, we started the study of a similar [Pt(IV)-phterpy] cationic compound, and the first results were already described in our previous work [21]. Now, we intend to proceed with this research activity on the synthesis, study and characterization of new photoactivatable complexes endowed with more favorable properties for their use as potential anticancer agents. Our aim is to obtain a compound that is inert and stable in its +4 oxidation state, preventing unwanted reduction reactions. However, such a compound should simultaneously display higher water solubility and the capacity of being activated by visible light instead of UV light. The synthesis started from the organometallic [PtI_2_(COD)] (where COD is 1,5-cyclooctadiene), obtained from [PtCl_2_(COD)], as previously described [31]. Then we coordinated the ligand phterpy to obtain complex [PtI(phterpy)][CF_3_SO_3_] (**3**), following the strategy reported in the literature [21]. Complex **3** was then reacted with Ag(OCOCH_3_) in DMF to obtain [Pt(OCOCH_3_)(phterpy)][CF_3_SO_3_], (**2**). The presence of the iodide ligand in complex **3** has proven to be necessary to obtain the complete substitution of the halide with the acetate ligand in complex **2**. In fact, the chloride ligand is not easily removed, as described in the Supplementary Materials (Paragraph 1 and Figures S1–S3 in the Supplementary Materials). Finally, **2** was oxidized with H_2_O_2_ in acetic acid, leading to Pt(IV) intermediates containing axial hydroxy/acetate ligands, whose structure is not depicted in Scheme 1, but their ^1^H-NMR spectra can be found in Figures S4 and S5 in the Supplementary Materials. A similar behavior was already found in a previous work [21]. The subsequent treatment of the reaction mixture with acetic anhydride converted all the hydroxy groups coordinated to the Pt metal centre to acetate ligands, leading to the one desired Pt(IV) complex, [Pt(OCOCH_3_)_3_(phterpy)][CF_3_SO_3_] (**1**), (Scheme 1).

**Figure 1:**
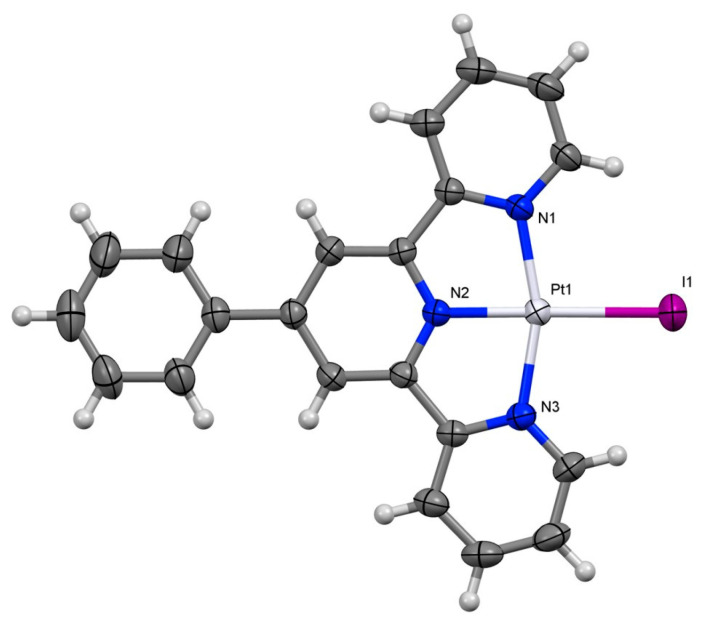
Crystal structure of complex **3**. Thermal ellipsoids are at 50% probability. Main bond distances (Å) Pt1−N1 2.030(3); Pt1−N2 1.942(2); Pt1−N3 2.025(3); Pt1−I1 2.5834(3). Crystal system: triclinic.

The aforementioned compounds **1**, **2** and **3** were fully characterized by means of elemental analysis, NMR, IR, UV-Vis, cyclic voltammetry and ESI mass spectrometry (for selected compound, **1**). The ^195^Pt NMR of compound **1** showed a broad downshift upon formation due to the Pt oxidation state: from −2405 ppm for the Pt(II) precursor to 1293 ppm for the Pt(IV) complex. The ^1^H, ^13^C NMR and IR spectra confirmed the presence of two new acetate ligands coordinated to the metal center, and the overall structure was further verified by means of ESI-MS. The complete description of the spectroscopic details of all the compounds is given in the Supplementary Materials (Figures S6–S18). Crystals suitable for X-Ray analysis were obtained after slow evaporation of a solution of compound **3** and **2** in CH_3_CN (CCDC 2002946 and 2121178, respectively). The crystal structure of compound **3** is reported in Figure 1 (additional details in Figures S19 and S20). The crystal structure of compound **2** is displayed in Figure S21, and the details on the unit cell, bond lengths and angles are reported in Tables S1–S4 in the Supplementary Materials. Unfortunately, it was not possible to obtain crystals also for the compound **1**.

The oxidation of complex **2** with hydrogen peroxide in acetic acid resulted in a mixture of two species, [Pt(OCOCH_3_)_2_(OH)(phterpy)][CF_3_SO_3_] and [Pt(OCOCH_3_)_3_(phterpy)][CF_3_SO_3_], as determined by ^1^H NMR (Figures S4 and S5 in Supplementary Materials) [32]. As we observed in a previous work [21], the axial hydroxy ligand is replaced by an acetate ligand as the reaction time goes on, and this could be the source of the minor species found: [Pt(OCOCH_3_)_3_(phterpy)][CF_3_SO_3_]. In any case, the successive carboxylation with acetic anhydride converts all the intermediates to the tricarboxylato complex **1**, as shown in Scheme 1.

Interestingly, the same oxidation reaction and carboxylation performed on compound **3** (with one iodide ligand in the equatorial plane) led to the pure triacetato compound **1** (Scheme 1), as evidenced by the ^1^H, ^13^C, ^195^Pt NMR spectra (Figures S22–S24) and ESI mass spectrum (Figures S25–S27). In this direct pathway, the iodide coordinated to Pt(II) needs to leave the coordination sphere, possibly during the oxidation step. Indeed, iodine clearly evaporated during the removal of the volatiles from the reaction mixture, and the subsequent carboxylation with acetic anhydride led to only one Pt(IV) species (complex **1**). On the other hand, when studying the same oxidation and subsequent carboxylation of the closely related [PtCl(phterpy)][CF_3_SO_3_], we did not find any species other than [Pt(OCOCH_3_)_2_Cl(phterpy)][CF_3_SO_3_] in the products, and this could be due to the ease of oxidation for I^−^ → I_2_ with respect to Cl^−^ → Cl_2_ [21]. Nonetheless, even if the direct transformation of **3** produced complex **1** of enough purity, we synthesized compound **2** to have a Pt(II) scaffold that we could use to compare the (photo)reduction products of **1**.

### 2.2. Stability in the Dark

The stability in physiological medium is an essential parameter to consider when dealing with Pt(IV) prodrugs, therefore we tested the stability of compound **1** under several circumstances (see Figures S28–S30 in the SI). Even if **1** was not degraded after one week in phosphate buffer (1 × 10^−2^ M, pH = 7.3; see Figure S28), we tested its stability when glutathione (GSH, one of the most abundant and powerful cellular reducing agents) [33] was present in the solution. We planned our experiment taking into consideration that the concentration of GSH would be in the mM range inside the cell, whereas that of the Pt(IV) compound would be in the μM range. Therefore, we added 500 equivalents of GSH (5 × 10^−3^ M) with respect to compound **1** (1 × 10^−5^ M) in phosphate buffer at 37 °C, and the reaction was monitored by UV-Vis. Interestingly, the reduction of compound **1** under these experimental conditions was not complete even after 8 h (see Figure S31 in Supplementary Materials).

We also evaluated the behavior of the Pt(II) complex **2** in aqueous solution to see if it underwent hydrolysis and/or aquation reactions. Its UV-Vis spectrum (Figure S32) did not show significant changes up to 2 days after the preparation of the solution and its ^1^H NMR spectrum (Figure S33) confirmed this trend up to 2 weeks. However, a closer look into the NMR spectrum revealed that there were two species present in solution, whose ratio remained unchanged over time, suggesting that an initial rapid equilibrium was established between **2** and its aquated form, which did not change with time. To obtain only one species, we added a large excess of sodium acetate, which suppressed the aquation equilibrium in D_2_O (Figure S34). This study is meaningful in the view of the photoreduction experiments carried out on the Pt(IV) complex, because it highlights that the reactivity of the platinum center becomes important only after the reduction to the Pt(II) oxidation state.

### 2.3. Irradiation Studies

The irradiation with the UV lamp (used as reference and comparison with previous work) [21] could transform an aqueous solution of **1** into the Pt(II) species in about 30 min, with a time constant of 498 s (Figures S35 and S36). However, a desirable improvement would be to induce the same photoreduction using longer wavelengths, avoiding the UV portion of the spectrum. To this aim, we irradiated an identical aqueous solution of **1** with a Xenon lamp equipped with a longpass filter that cuts all the wavelengths shorter than 400 nm (Figure S37). In this condition, more than 145 min of continuous irradiation are required to produce an appreciable amount of Pt(II). This remarkable difference is thought to correlate with the poor absorbance of **1** in the visible portion of the spectrum. Indeed, when the wavelength filter was removed from the excitation source, the reduction of the complex was much faster (Figure S38).

Finally, to investigate the nature of the species formed after the irradiation, UV light was shone on a CD_3_CN solution of **1** in an NMR tube. We could confirm that the photoreduced Pt(II) species corresponds to complex **2**, as shown in the Supplementary Materials (Figures S39–S41), during a process that lasted for 100 min of irradiation under our experimental conditions.

### 2.4. Photocatalysis Studies

**Figure 2:**
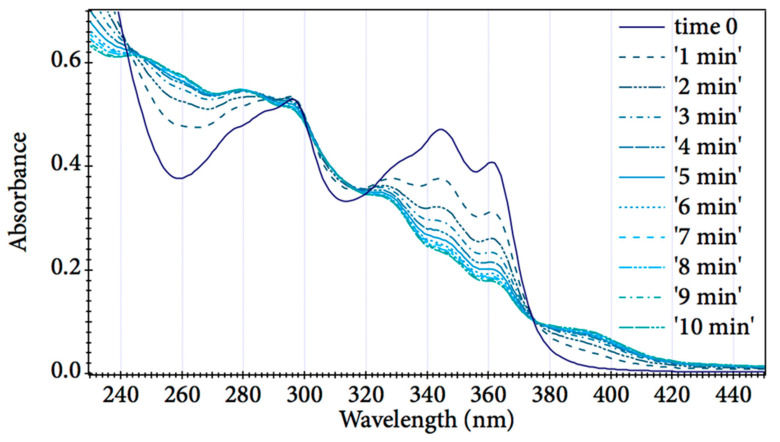
Photoconversion of **1** (2 × 10^−5^ M) with NADH (4 × 10^−5^ M) and FMN (1 × 10^−6^ M) in PB (5 × 10^−2^ M, pH = 7.0); λ = 460 nm.

To achieve higher photoactivation efficiency under visible light excitation, we successfully employed a new photocatalytic approach that uses flavin mononucleotide (FMN) as catalyst and an electron donor (i.e., NADH or MES buffer) to induce the reduction of compound **1**. Under these circumstances, our studies indicate that an LED lamp operating at 460 nm (6 mW × cm^−2^) is sufficient to induce a faster and more efficient reduction process. As shown in Figure 2, the photoreduction of **1** in cuvette was complete after 10 min of irradiation, using an excess of NADH (2 eq) and with 5% of catalyst load with respect to the substrate, with a time constant of 130s (see Figure S42a–c).

The photocatalysis experiment just described was repeated, slightly decreasing the concentrations of **1** and NADH but leaving their ratio constant (conditions detailed in Figure S43). As a result, a nearly identical value of 135 s for the time constant was measured (Figures S43 and S44), which supports the robustness of the catalytic system. More importantly, we highlight that the ratio between the time constants obtained with UV irradiation (498 s) and with the catalytic system is 3.7. This means that the photo-catalytic reduction was almost four times faster than the photo-reduction at 365 nm, and that the reduction of complex **1** was achieved using visible light at 460 nm, with a red shift of almost 100 nm with respect to the UV irradiation. These two aspects, taken together, overcome (in part) the biggest problems encountered in the photoactivation of platinum prodrugs.

In addition, other experiments were set up to investigate if the concentrations of the substrates have an effect on the rate of the reaction. When the amount of NADH was increased with respect to substrate **1** (10:1), an even faster reduction rate was obtained—more than five times with respect to UV irradiation (time constant of 93 s, Figures S45 and S46). Finally, stability studies were performed to confirm that solutions containing **1** and NADH alone were stable in the dark (Figures S47–S49) and under irradiation at 460 nm (Figure S49). Reduction to Pt(II) was not visible after several hours in the dark, and irradiation did not cause substantial reduction in the absence of FMN. Moreover, the presence of FMN, together with **1** and NADH in the dark, did not produce appreciable spectral changes in the photo-reduction timescale (Figure S50).

Additionally, in this case we wanted to investigate the chemical nature of the products of the irradiation; therefore, the photocatalytic experiments were followed by ^1^H-NMR in aqueous solution. MES buffer was used in place of both phosphate buffer and NADH to control the pH of the reaction and as electron donor to regenerate the flavin after the reduction process in the catalytic cycle [27]. This choice was due to the fact that NADH caused a rapid conversion of **1** in the dark at the higher concentration required for ^1^H-NMR (~10^−4^ M), whereas MES caused a smaller conversion in the dark (only 10% of the initial substrate after 48 h, Figure S51). Photocatalytic reactions using FMN 1 × 10^−6^ M reached the full reduction of **1** after only 120 s (at 460 nm Figure S52). The turnover frequency (TOF) for FMN and **1** was 5 ± 2.37 s^−1^, and the turnover number (TON) was 500. However, as anticipated, the stability of **1** was an issue; in fact, when FMN (1 × 10^−6^ M) was added to the MES buffered solution, a more marked decomposition (30% of the initial substrate after 24 h, Figure S53) was observed, demonstrating that a weak residual catalytic activity was also exerted, even in absence of light. Moreover, the photostability of **1** in MES buffer, under irradiation at 460 nm and without FMN, was found to be even lower; the Pt(II) species was detectable after only 5 min of irradiation and, after 10 min, **1** was completely reduced (Figure S54).

We speculate that some kind of interaction takes place between MES and **1** because the reduced stability of **1** in such conditions is dependent on concentration. However, we did not investigate further this aspect because it is beyond the purpose of this work.

### 2.5. Electrochemistry

Electrochemical methods were employed in this work to study the reduction potential of complex **1** and the species generated following its reduction. It is necessary to determine the reduction potential, since it is a parameter closely related to the stability in a reducing environment (like in the cancer cell) [34]. Complex **1** was studied both in DMSO and in aqueous buffered solution with cyclic voltammetry (CV) and linear sweep voltammetry (LSV). Light exposure was carefully avoided during these studies to be sure that the reduction of the species was due only to the negative electrode potential. DMSO was chosen as solvent because all the redox processes are well visible in this solvent and also because it allows a direct comparison with the reduction potential of [Pt(OCOCH_3_)_2_Cl(phterpy)][CF_3_SO_3_], as previously studied [21].

**Figure 3:**
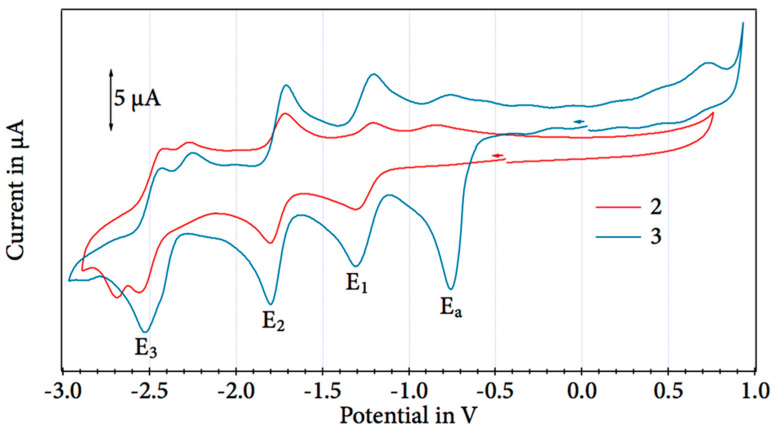
CVs of compounds **2** and **1** (10^−3^ M) in DMSO with 0.1 M [NBu_4_][CF_3_SO_3_]. The direction of the arrows indicates the direction of the scan.

The CVs of **2** and **1** (DMSO solvent) are reported in Figure 3, and the formal reduction potentials for the observed electron transfers are compiled in Table 1. A Teflon encapsulated carbon-glassy stick was used as the working electrode, whereas the counter and reference electrodes were a platinum-gauze and platinum wire (*quasi* reference), respectively; tetrabutylammonium triflate ([NBu_4_][CF_3_SO_3_]) was used as supporting electrolyte.

Compound **2** showed three reduction peaks going toward the negative scan of the potential, similarly to the previously studied [PtCl(phterpy)][CF_3_SO_3_], which is the Pt(II) analogue of **2** with the chloride in place of the acetate [20]. In the present study, the first reduction peak was located at −1.28 V (E_1_), and it was electrochemically *quasi*-reversible; the second was at −1.78 V (E_2_), and it was chemically and electrochemically reversible [35]. The third process was located around −2.6 V (E_3_); it was irreversible and seems to be a double reduction. The most striking difference with [PtCl(phterpy)][CF_3_SO_3_] was the more negative values of the reduction peaks E_1_, E_2_ and E_3_ by ~80 mV, a behavior that other authors also highlighted when studying Pt(IV) compounds with equatorial acetates or chlorides [36]. A possible explanation for this could be the major donor strength of the acetate with respect to the chloride [37].

Compound **1** underwent four sequential reductions: the first was chemically irreversible and happened at −0.76 V (E_a_) with respect to ferrocene (Figure 3), whereas the other reduction waves fell at the same potential as compound **2**. To determine which reduction process was associated with E_a_, we used hydrodynamic voltammetry; in our system, the current for the process E_a_ was double with respect to E_1_ and E_2_ (Figure S55). Therefore, the electrons involved in the E_a_ reduction must be double with respect to E_1_ and E_2_, so we deduced that the reduction process located at −0.76 V was the Pt(IV) to Pt(II) reduction. After the reduction, the axial ligands were rapidly lost from the Pt complex, generating compound **2**; in fact, the subsequent scan toward the negative potential was almost superimposable to that of compound **2**. As a comparison with [PtCl(OAc)_2_(phterpy)]^+^, which has two acetates in the axial positions and one chloride in the equatorial position [21], we found that compound **1** was more stable; its reduction potential was −0.76 V, whereas that of [PtCl(OAc)_2_(phterpy)]^+^ was −0.64 V. The stabilization effect provided by one equatorial acetate with respect to chloride was 120 mV, similarly to what was already reported in the literature [36]. This behavior perfectly matches with our goal to obtain a stable and inert compound that should be reduced only under light exposure.

Since compound **1** was soluble enough in aqueous medium to record its CV, we evaluated its reduction potential in phosphate buffer (5 × 10^−2^ M, pH = 7.0, [**1**] = 5 × 10^−4^ M), Figure S56. With Ag│AgCl as a reference, we obtained a value of −0.18 V ± 0.06 V, which was close to the reduction potential of Satraplatin (−0.25 V with respect to Ag|AgCl) [38]. The standard deviation, 0.06 V, is quite high because subsequent scans gave us different results, even if the electrode surface was polished between subsequent measures. However, a similar high standard deviation was already reported for similar compounds in water-based solutions [39].

In light of the electrochemistry experiments results, we can argue that the ease of reduction of the studied Pt(IV) complex can be closely related to environmental conditions.

### 2.6. Binding Studies to Relevant Biomolecules

**Figure 4:**
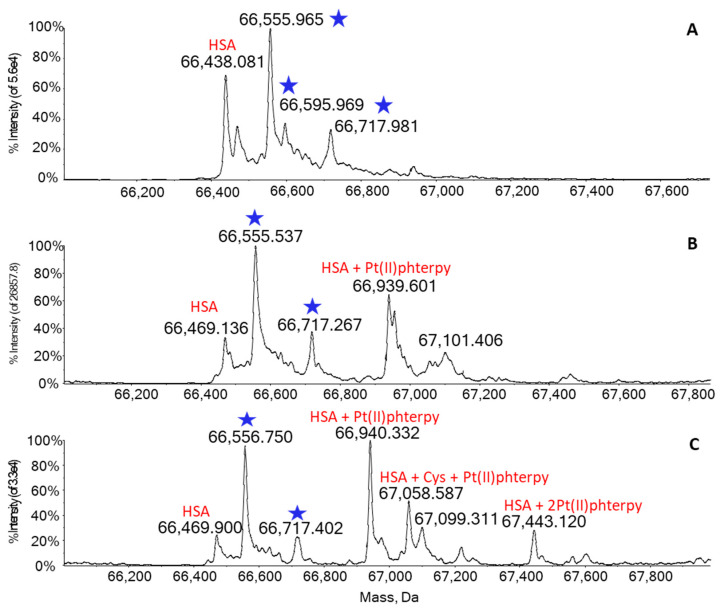
Deconvoluted ESI mass spectra of **2** (1 × 10^−4^ M) and **1** (5.5 × 10^−5^ M) incubated with HSA (1.4 eq per platinum) at room temperature for 24 h in ammonium acetate solution (2 × 10^−3^ M, pH = 6.8). Panel (**A**)—complex **1** in the dark; panel (**B**)—complex **1** irradiated for 5 min at 365 nm (UV lamp); panel (**C**)—complex **2**. Blue stars indicate post-translational modification of HSA.

After the assessment of the reduction potential of the Pt(IV) complex **1**, we evaluated the reactivity features against biomolecular targets both in the dark and after light irradiation. Since human serum albumin (HSA, Figure S57) is the most abundant protein in plasma, with a concentration of about 6 × 10^−4^ M, it represents the first biomolecule that a drug encounters in the bloodstream [40]. Furthermore, this protein is commonly accepted as the main transport protein in the blood, so it is mandatory to assess the reactivity behavior towards this biomolecule, especially in the case of metal-based drugs [3]. To this aim, we incubated **1** with HSA in the dark, and we reported the related ESI mass spectrum (Figure 4, panel A).; no binding occurred, and only the peak of HSA (66,438 Da) was present in the spectrum. The other peaks marked with blue stars belong to some post-translational modification of HSA normally present in the serum-extracted protein [5,41]. On the contrary, when the **1**/HSA mixture was irradiated at 365 nm, an extra peak attributable to the mono-adduct (HSA-Pt(II)phterpy) at 66,939 Da was well-detectable (Figure 4, panel B). Finally, to confirm the nature of this last peak, we also incubated **2** (the Pt(II) analogue of **3**) with the protein. It turned out that the pharmacophore [Pt(phterpy)]^2+^ is able to bind the HSA forming mono- and bis-adducts (66,940 Da and 67,443 Da, respectively). Lastly, the peak at 67,058 Da corresponds to a naturally cysteinylated form of HSA bonded with the [Pt(phterpy)]^2+^ moiety.

Since the main accepted biological target for Pt-based compounds is DNA, we also investigated the reactivity of these molecules toward a single-filament oligonucleotide (ODN2) as a mimic for the reactivity of our compounds toward the DNA [42,43]. ODN2 has the sequence 5′-CTACGGTTTCAC-3′ and a molecular weight of 3596.36 Da, and it is endowed with two adjacent guanine residues that represent the main binding site for Pt(II) compounds.

**Figure 5:**
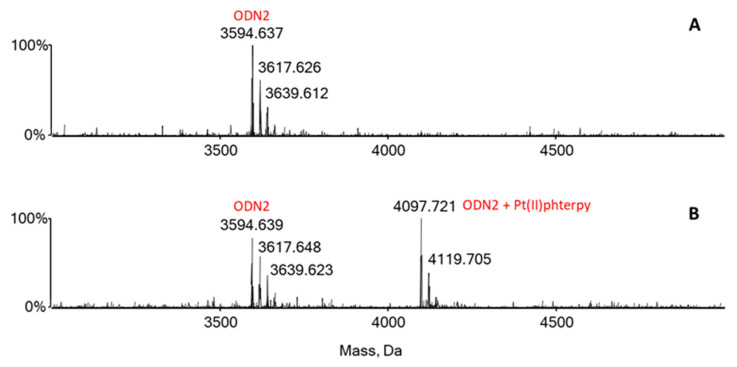
Deconvoluted ESI mass spectra of ODN2 (10^−4^ M) incubated at room temperature for 24 h with **1** (1:1 metal to ODN ratio) in LC-MS water (with 4% DMSO and 3% acetone). Panel (**A**)—complex **1** in the dark; panel (**B**)—complex **1** irradiated for 5 min at 365 nm (UV lamp).

The reactivity of compound **1** with the oligonucleotide is depicted in Figure 5. Newly, we highlighted that complex **1** kept in the dark was also completely unreactive with the genomic target (Figure 5, panel A), and the only visible peak at 3595 Da (and the relative sodiated forms) was attributable to ODN2. Nevertheless, when compound **1** was irradiated with UV light, its reactivity was completely restored, giving rise to a relevant adduct formation. In particular, the peak at 4097 Da (Figure 5, panel B) was related to the ODN2/ Pt(II)phterpy mono-adduct.

### 2.7. LogP Values

The solubility of **1** in water was found to be 0.5 × 10^−3^ M, which was high enough to allow the recording of its ^1^H NMR spectrum and its cyclic voltammetry in a buffered solution. Since acetate ligands are known to promote aqueous solubility, we measured the partition coefficient between water and octanol of **1**, LogP, by means of Inductively Coupled Plasma—Atomic Emission Spectrometry (ICP-AES). Its value (Table 2) was compared to other compounds reported in a previous work [21], which are listed with their molecular structure to highlight the effect of single ligands. Data show that acetate ligands determine a higher affinity for the water phase with respect to chlorides.

Measured as comparison and reference, cisplatin exhibits a LogP that is more negative and, hence, a more hydrophilic character than **1**. However, the LogP value does not refer to the intact cisplatin, but rather to the product of its aquation.

## 3. Materials and Methods

### 3.1. General Considerations

Chemicals and solvents were of reagent grade and used without further purification unless otherwise stated. NMR measurements were recorded with a Varian Gemini 200BB spectrometer or a Bruker Avance II DRX400 instrument equipped with a BBFO broad-band probe. All spectra were recorded at room temperature. NMR chemical shifts (δ) are reported in parts per million (ppm) and referenced as described below. ^1^H and ^13^C{^1^H} NMR spectra were calibrated on the residual solvent peaks, and chemical shifts were expressed relative to tetramethylsilane. ^195^Pt{^1^H} NMR spectra were referenced externally using a standard of K_2_PtCl_6_ in H_2_O. The samples were prepared in DMSO-*d*_6_, acetonitrile-*d*_3_ or in D_2_O at room temperature. Carbon, hydrogen and nitrogen analyses (EA) were performed on a Vario MICRO cube instrument (Elementar, Langenselbold, Germany). IR spectra of neat samples were recorded with a PerkinElmer “Spectrum One” FT-IR spectrometer, with an ATR technique. The pH-meter used was a Ω Metrohm 713. Compounds **2** and **1** are soluble either in aqueous solution (up to a concentration of 5 × 10^−4^ M) or in organic solvents like acetonitrile, DMF or DMSO.

### 3.2. Synthesis

***Caution***! Hydrogen peroxide in the presence of organic solvents can form explosive peroxides. All synthesis and purifications were carried out under dim light conditions, avoiding contact with direct light.

**PtI_2_(COD).** The synthesis was performed according to the literature procedure [31]. Briefly, PtCl_2_(COD) (0.194 g, 0.518 mmol) was suspended in 20 mL of acetone and NaI (0.171 g, 1.12 mmol) was added; the color turned yellow immediately. After 2 h, the solvent was evaporated in vacuo and the residue was suspended in 10 mL of deionized water, stirring for 20 min. The solid was filtered, washed with water and dried. Yield 0.272 g, 94%. C_8_H_12_I_2_Pt (557.07 g × mol^−1^): calculated C 17.25, H 2.17; found C 17.06, H 2.04. ^1^H NMR (CDCl_3_): δ 5.76 (s with ^195^Pt satellites, ^2^*J_PtH_* = 67 Hz, 4H); 2.46 (m, 4H); 1.93 (m, 4H). ^195^Pt{^1^H} NMR (CDCl_3_): δ − 4329 ppm.

**[PtI(phterpy)][CF_3_SO_3_] (3).** A solution of AgCF_3_SO_3_ (0.123 g, 0.478 mmol) in 5 mL of CH_2_Cl_2_ and 1 mL of CH_3_CN was dropped in a mixture containing PtI_2_(COD) (0.241 g, 0.432 mmol) in 24 mL of CH_2_Cl_2_, stirring under aluminium foil. After 3 h, the yellow suspension was slowly passed through a small pad of Celite, resulting in a yellow solution of [PtI(CH_3_CN)(COD)][CF_3_SO_3_]. This was dropped in a solution of phterpy (0.147 g, 0.476 mmol) in 10 mL of CH_2_Cl_2_, stirring overnight. The product was collected, washed with CH_2_Cl_2_ and dried in vacuo. Yield 0.310 g, 92%. C_22_H_15_F_3_IN_3_O_3_PtS (780.42 g × mol^−1^): calculated C 33.86, H 1.94, N 5.38; found C 33.88, H 1.86, N 4.93. ^1^H NMR (DMSO-*d*_6_): δ 9.50 (m with ^195^Pt satellites, ^3^*J_HH_* = 5.8 Hz, 2H, H6); 9.00 (s, 2H, H3′); 8.87 (m, ^3^*J_HH_* = 8.0 Hz, 2H, H3); 8.51 (m, ^3^*J_HH_* = 8.0 Hz, 3JHH = 7.7 Hz, 2H, H4); 8.22 (m, 2H); 7.88 (m, ^3^*J_HH_* = 7.7 Hz, ^3^*J_HH_* = 5.8 Hz, 2H, H5); 7.70 (m, 3H). ^195^Pt NMR (DMSO-d6): δ − 2858 ppm.

**[Pt(OCOCH_3_)(phterpy)][CF_3_SO_3_]·2H_2_O (2).** AgOCOCH_3_ (5.0 mg, 0.028 mmol) was suspended in 7 mL of DMF and added to a solution of [PtI(phterpy)][CF_3_SO_3_] (19 mg, 0.024 mmol) in 2 mL of DMF. The resulting suspension was stirred in the dark for 24 h, then filtered and concentrated in vacuo; addition of diethyl ether (35 mL) resulted in a yellow-orange suspension. The solid was filtered, washed with diethyl ether and dried in vacuo. Yield 14 mg, 78%. C_24_H_22_F_3_N_3_O_7_PtS (748.59 g × mol^−1^): calculated C 38.51, H 2.96, N 5.61, S 4.28; found C 38.71, H 2.94, N 5.65, S 4.41. ^1^H NMR (DMSO-*d*_6_): δ 8.98 (s, 2H); 8.87 (m, 2H); 8.58 (m, 4H); 8.20 (m, 2H); 7.97 (m, 2H); 7.69 (m, 3H); 2.16 (s, 3H). ^13^C NMR (DMSO-*d*_6_): δ 176.1 (C=O); 157.5; 154.8; 152.5; 151.1; 142.3; 134.7; 131.5; 129.4; 128.9; 127.8; 125.7; 121.2; 23.3 (CH_3_). ^195^Pt NMR (DMSO-*d*_6_): δ − 2398 ppm.

**[Pt(OCOCH_3_)_3_(phterpy)][CF_3_SO_3_]·H_2_O (1).** Compound **2** (11 mg, 0.016 mmol) was dissolved (yellow solution) in 2 mL of acetic acid, and H_2_O_2_ (0.5 mL, 4.8 mmol) was added, stirring at room temperature in the dark for 24 h. The resulting solution (very pale yellow) was dried in vacuo, then 4 mL of deionized water were added, and the system was dried again in vacuo, to get rid of all the possible H_2_O_2_ left. Acetic anhydride (2 mL, 21 mmol) was added to the crude product, and, after 20 h, the solid was precipitated with diethyl ether, filtered, washed with diethyl ether and dried in vacuo. Yield 11 mg, 82%. C_28_H_26_F_3_N_3_O_10_PtS (848.66 g × mol^−1^): calculated C 39.63, H 3.09, N 4.95, S 3.78; found C 39.59, H 3.06, N 4.99, S 3.59. ^1^H NMR (CD_3_CN): δ 9.18 (dd with ^195^Pt satellites, ^3^*J_PtH_* = 25 Hz, ^3^*J_HH_* = 5.8 Hz, ^4^*J_HH_* = 1.3 Hz, 2H, H6); 8.74 (s, 2H, H3′); 8.59 (dd, ^3^*J_HH_* = 7.9 Hz, ^4^*J_HH_* = 1.4 Hz, 2H, H3); 8.50 (ddd, ^3^*J_HH_* = 7.9 Hz, ^3^*J_HH_* = 7.8 Hz, ^4^*J_HH_* = 1.4 Hz, 2H, H4); 8.08 (m, 2H, Ha); 7.99 (ddd, ^3^*J_HH_* = 7.8 Hz, ^3^*J_HH_* = 5.8 Hz, ^4^*J_HH_* = 1.3 Hz, 2H, H5); 7.73 (m, 3H, Hb, c). ^13^C NMR (DMSO-*d*_6_): δ 174.8 (axial acetates, C=O); 174.7 (equatorial acetate, C=O); 157.3; 156.0; 153.4; 152.1; 144.5; 133.9; 132.3; 129.5; 129.4; 128.6; 126.9; 122.9; 22.8 (equatorial acetate, C=O); 21.3 (axial acetate C=O). ^195^Pt NMR (DMSO-*d*_6_): δ 1294 ppm.

### 3.3. X-ray Crystallography

Single crystals were glued at the end of glass fibres. Single-crystal X-ray Diffraction have been performed at room temperature by means of a Bruker SMART Breeze CCD diffractometer equipped with graphite monochromated Mo-Kα radiation (λ = 0.71073 Å). All the structure solutions were found using the automated direct methods contained in SHELXS-97 program [45]. The structure refinement was performed by using the same program. Details of the crystal data and of refined structure parameters for compounds **1** and **2** have been deposited with the Cambridge Crystallographic Data Centre and may be obtained by quoting the references to this paper. The deposition numbers are CCDC 2002946 and CCDC 2121178, respectively.

### 3.4. Stability Studies in Solution

UV-Vis electronic spectra were recorded on a Cary 60 spectrophotometer, using quartz cells with 1.0 cm path length at room temperature in aqueous buffered solution ([**1**] = 3 × 10^−5^ M, phosphate buffer 10 × 10^−3^ M, pH = 7.3) for up to 9 days. A Perkin Elmer λ650 spectrophotometer was used to perform the stability studies with the GSH reducing agent ([**1**] = 1 × 10^−5^ M, [GSH] = 5 × 10^−3^ M, in phosphate buffer 50 × 10^−3^ M, pH = 7.0) at 37 °C for 8 h. ^1^H NMR spectra of **1** were recorded either in DMSO-*d*_6_ or in acetonitrile-*d*_3_ at room temperature for at least 7 days with the Bruker Avance II DRX400 spectrometer. Care was used in order to perform all the experiments in the dark or under dim light conditions.

### 3.5. Irradiation Studies

As sources for irradiation, we used a UV lamp Spectroline^TM^ model ENF-260C/F (230 volts, 50 Hz, 0.17 A), Spectronics Corporation Westbury, NY, USA, operating at 365 nm (the optical density of this lamp was measured to be 5.5 mW × cm^−2^) or a Xenon lamp (Muller Elektronik Optik, Salzkotten, Germany, Zundgerat XHZ 100, average ~70 W) with a longpass filter at 400 nm (Thorlabs, Newton, NJ, USA). Solutions of **1** in phosphate buffer (10 × 10^−3^ M, pH = 7.3) were irradiated with both lamps, and their electronic spectra were recorded until complete consumption of the Pt(IV) starting material. In addition, a solution of **1** in acetonitrile-*d*_3_ was irradiated with the UV lamp recording its ^1^H NMR spectrum over time, until all the Pt(IV) starting material was transformed into the Pt(II) product.

### 3.6. Photocatalysis Studies

The irradiation of the samples was performed using an LED lamp (460 nm, 6 mW × cm^−2^) at room temperature in the presence of O_2_, and the results were analyzed both via UV-Vis spectroscopy and via ^1^H NMR spectroscopy.

In a typical NMR experiment, compound **1** (5 × 10^−4^ M) was dissolved in MES buffer (1.8 × 10^−2^ M, pH = 6.0) together with Riboflavin 5′-monophosphate (FMN, 10^−6^ M) that acts as a photocatalyst, and the reaction progression was evaluated monitoring the appearance and disappearance of diagnostic peaks corresponding to the coordinated and free acetate ligands of **1**. The release of axial acetate ligands corresponds to the formation of biologically active Pt(II) compounds. Turnover number (TON, calculated by dividing the total moles of the transformed substrate by the moles of catalyst) and turnover frequency (TOF, calculated by dividing the moles of the transformed substrate by the moles of catalyst per unit of time, when the conversion was around 30% of the total) were determined by quantifying the amount of converted **1** via ^1^H NMR spectroscopy.

In the UV-Vis experiments, **1** was dissolved in phosphate buffer (5 × 10^−2^ M, pH = 7.0) together with NADH (in excess) and FMN (in catalytic amount), and the spectra were recorded until consumption of the starting material.

### 3.7. Time Constant Evaluation

Absorbance at a specific wavelength was plotted against the irradiation time, and an exponential decay resulted. Linearization of the data was done by plotting the ln((A_t_ − A_inf_)/(A_0_ − A_inf_)) against the irradiation time. From the negative slope, we obtained the value of tau (τ) using the software Igor Pro version 6.22A, as reported in Section S7 in the Supplementary Materials. It should be noted that the calculated τ values are consistent in the frame of this investigation, allowing a comparison between the different irradiation techniques. Although, they are not absolute values, because the rate of a photochemical reaction depends upon several parameters (the emission spectrum of the lamp, the number of photons per second, the quantum yield, the molar attenuation coefficient, etc.).

### 3.8. Electrochemistry

Electrochemical measurements were recorded on a PalmSens4 instrument working with PSTrace5 electrochemical software and were performed at room temperature either in DMSO solution ([NBu_4_][CF_3_SO_3_] (0.1 M) as the supporting electrolyte) or in aqueous solution (phosphate buffer 5 × 10^−2^ M, pH = 7.0 as supporting electrolyte).

*Cyclic voltammetry (CV).* CV was performed in a three-electrode cell: the working electrode was a Teflon encapsulated carbon-glassy stick, the counter electrode was a platinum-gauze, and the reference electrode was a platinum wire (*quasi* reference, used for the measurements in DMSO) or a Ag|AgCl (for the aqueous measurements). Prior to measurements, the glassy carbon working electrode was polished according to the following procedure: manual rubbing with 0.3 μm Al_2_O_3_ slurry in water (eDAQ) for 2 min, then sonication in ultrapure water for 10 min, manual rubbing with 0.05 μm Al_2_O_3_ slurry in water (eDAQ) for 2 min, then sonication in ultrapure water for 10 min. The solution of the supporting electrolyte was introduced into the cell and deaerated by bubbling Ar. The potential of the working electrode was cycled several times between the cathodic and anodic limits to determine the solvent window. The analyte was then introduced into the cell with a concentration of ∼1 × 10^−3^ M (5 × 10^−4^ M when the CV was performed in phosphate buffer), and the voltammograms were recorded (0.1 V × s^−1^) under a blanket of Ar. During the measurements in DMSO, a small amount of Cp_2_Fe (bis(η5-cyclopentadienyl)iron, ferrocene) was added to the solution of the analyte and a further voltammogram was recorded: potentials were determined by placing the Cp_2_Fe^+^/Cp_2_Fe couple at 0.0 V (under these experimental conditions, the one-electron oxidation of Cp_2_Fe occurs at E° = +0.50 V vs. Ag|AgCl) [46]. In the case of Pt(IV) compounds, the cell was covered with aluminium foil to avoid direct light exposure.

*Hydrodynamic voltammetry (linear sweep voltammetry, LSV).* LSV with the renewal of the diffusion layer made use of a rotating disk electrode Metrohm 628-10, consisting of a glassy carbon surrounded with an insulating Teflon.

### 3.9. Interaction with the Biomolecules

Interactions between the Pt compounds and human serum albumin (HSA) or monofilament DNA fragment (ODN2) were assessed by high-resolution ESI-MS, following a well-established protocol [39,47,48]. In the case of HSA, **2** (1 × 10^−4^ M) and **1** (5.5 × 10^−5^ M) were reacted separately with the protein (1.4 eq. of HSA per platinum) in 2 × 10^−3^ M ammonium acetate solution at pH 6.8. In the case of ODN2, the Pt complexes to target biomolecule ratio was 1:1 with a final ODN concentration of 10^−4^ M in LC-MS water. For both targets, two different sets of reaction mixtures were produced: one series was irradiated with the 365 nm UV lamp for 5 min, and in the second series all samples were kept in the dark. Each sample was then incubated for 24 h at room temperature.

Aliquots were sampled and diluted with LC-MS water to a final concentration of 5 × 10^−7^ M in the case of HSA and 10^−5^ M for ODN. Respective ESI-MS spectra were acquired through direct infusion at 5 μL min^−1^ flow rate in a TripleTOF^®^ 5600^+^ high-resolution mass spectrometer (Sciex, Framingham, MA, USA) equipped with a DuoSpray^®^ interface operating with an ESI probe. The ESI source parameters were optimized and were as follows: in the case of HSA, 0.1% of formic acid was added to the sample solutions, and the spectra were recorded in positive polarity, Ionspray Voltage Floating 5500 V, Temperature 37 °C, Ion source Gas 1 (GS1) 40 L min^−1^; Ion source Gas 2 (GS2) 0 L min^−1^; Curtain Gas (CUR) 30 L min^−1^, Declustering Potential (DP) 200 V, Collision Energy (CE) 10 V.

In the case of ODN, 1% of triethylamine was added to the sample solutions and the spectra were recorded in negative polarity, Ionspray Voltage Floating −4500 V, Temperature 37 °C, Ion source Gas 1 (GS1) 40 L min^−1^; Ion source Gas 2 (GS2) 0 L min^−1^; Curtain Gas (CUR) 25 L min^−1^, Declustering Potential (DP) −30 V, Collision Energy (CE) −10 V. For acquisition, Analyst TF software 1.7.1 (Sciex, Framingham, MA, USA) was used and deconvoluted spectra were obtained by using the BioToolKit micro-application v.2.2 embedded in PeakView™ software v.2.2 (Sciex).

### 3.10. LogP Determined with ICP-AES

The octanol-water partition coefficient was determined by a modified shake-flask method [49]. The determination of platinum concentration in the water/octanol solutions was performed with a Varian 720-ES inductively coupled plasma atomic emission spectrometer (ICP-AES) equipped with a CETAC U5000 AT+ ultrasonic nebulizer, in order to increase the method sensitivity. Before analysis, fixed volumes of samples were moved in vials and digested in a thermoreactor at 80 °C for 3 h with 1 mL of aqua regia (HCl supra-pure grade and HNO_3_ supra-pure grade in 3:1 ratio) and 5 mL of ultrapure water (≤18 MΩ). Samples were spiked with 1 ppm of Ge used as an internal standard and analyzed. Calibration standards were prepared by gravimetric serial dilution from a commercial standard solution of Pt at 1000 mg L^−1^. The wavelength used for Pt was 214.424 nm, whereas for Ge the line at 209.426 nm was used. The operating conditions were optimized to obtain maximum signal intensity, and, between each sample, a rinse solution of HCl supra-pure grade and HNO_3_ supra-pure grade at a 3:1 ratio was used to avoid any “memory effect”. The logP value is defined as log_10_([complex]oct/[complex]wat). The final value was reported as the mean of three determinations.

## 4. Conclusions

Pt(IV) photoactive compounds represent a promising class of innovative anticancer agents; however, the limitation they generally suffer is related to the harsh UV-light conditions required to photoreduce Pt(IV) complexes to their biologically active Pt(II) counterparts. In the perspective of future clinical studies, UV light needs to be avoided because it does not penetrate deeply into the skin, and because it can damage the epidermis upon prolonged illumination. Therefore, a faster and milder approach for the photoactivation in the visible range is needed, and the catalytic photoactivation with flavin has already shown promise for this purpose. In this work, we could quantitatively show that the riboflavin-catalyzed activation was not only faster than the UV activation, but it was achieved with visible light, at 460 nm instead of 365 nm. These preliminary studies pointed out that the time constant (τ) for the reduction was about four times lower with respect to direct irradiation, giving a mean value of 133 s instead of 498 s. Importantly, the time constant for the activation of **1** was even lower (93 s) when the electron donor was present in a larger excess.

However, for completeness of clarity, we also reported that the NMR experiments showed an appreciable reduction of the substrate **1** in solution, even in dark conditions. Contrariwise, the same experiment conducted with the UV-Vis spectrophotometer did not reveal any significant reduction process. The different behavior could be explained considering the much lower concentration of **1**, flavin and cofactors used in the UV-Vis experiments (**1** was ~10^−5^ M) with respect to the NMR experiments (**1** was ~10^−3^ M). We can speculate that in relatively high concentrations the entire system could be less stable because of aggregation between the species, undergoing a partial and early reduction. However, we have to consider that in our previous study we reported the IC_50_ value in A2780 and A2780cis cell lines (9.4 ± 0.9 and 11.5 ± 0.7 μM, respectively) and the Pt uptake value (1 × 10^−8^ μg Pt/cell) for very close-related compounds [21]. These values reflected the real Pt concentration in cells when it was administered to a cell culture and are closer to the UV-Vis experiments conditions than the NMR ones. Complex **1** was designed to overcome some issues already encountered in the previously mentioned study^21^; in particular, it was designed to improve its water solubility, which is now up to 0.5 × 10^−3^ M, and its chemical stability in the Pt(IV) oxidation state, which is now 120 mV more stable, as reported by electrochemical studies.

Finally, as proof of concept, the reactivity of **1** was tested towards a representative model protein, i.e., HSA, and one monofilament oligonucleotide, i.e., ODN2. The reactions were followed by high-resolution ESI mass spectrometry, revealing that **1** was completely unreactive towards these two model biomolecules. Once activated by photoreduction, the resulting Pt(II) compound was highly reactive, giving rise to an extensive adduct formation both in the case of HSA and ODN2.

We believe that the strategy of catalyzed photoactivation could be of considerable interest in the field of metal-based drugs, since it avoids most parts of the unwanted off-target reactions that are typical of conventional Pt-based chemotherapeutics. In the future, the compounds designed to be activated by visible light need to demonstrate in vitro their superiority with respect to conventional compounds. This should be done with specifically designed cellular studies that take into account the photoactivation step. Moreover, the stability of photoactive compounds before the activation needs to be thoroughly assessed in the tumor-reducing environment. This experiment is essential to ensure the absence of undesired reactions, but it might be extremely difficult to be reproduced accurately in vitro. Clearly, we still have a way to go in this field, but important steps have been done, being that in situ catalytic photoactivation is one of the promising “targeted” chemotherapies.

## Figures and Tables

**Table 1:** Formal electrode potentials (V vs. FeCp_2_) and peak-to-peak separations (ΔEp in mV) in 0.1 M [NBu_4_][CF_3_SO_3_]/DMSO solution for the cited redox changes.

Compound	E_a_	E^0′^_1_	ΔE_p_(1) ^[a]^	E^0′^_2_	ΔE_p_(2) ^[a]^	E_3_^[b]^
2	/	−1.28	110	−1.78	92	−2.53
1	−0.76	−1.28	104	−1.78	96	−2.51

^[a]^ Measured at 0.1 V × s^−1^. ^[b]^ Peak potential value for irreversible processes.

**Table 2:** LogP values of selected complexes.

Compound	LogP
[PtCl_3_(phterpy)][CF_3_SO_3_] ^[a]^	−0.5 ^[a]^
[PtCl(OCOCH_3_)_2_(phterpy)][CF_3_SO_3_] ^[a]^	−1.5 ^[a]^
[Pt(OCOCH_3_)_3_(phterpy)][CF_3_SO_3_], 1	−1.6
Cisplatin, CDDP	−2.4 ^[b]^

^[a]^ Compounds and values from a previous work [21]. ^[b]^ Value obtained from the literature [44].

## Data Availability

Not applicable.

## Supplementary Materials

The following supporting information can be downloaded at: https://www.mdpi.com/article/10.3390/ijms24021106/s1.
