# Supplementary material for: Synthesis, Characterization and Photoactivation Studies on the Novel Pt(IV)-Based [Pt(OCOCH3)3(phterpy)] Complex
Source: Int J Mol Sci. 2023 Jan 6;24(2):1106. doi: 10.3390/ijms24021106 (PMC9864011; doi:10.3390/ijms24021106)
Supplement: Supplementary file 1 [file ijms-24-01106-s001.zip › Canil_SupplementaryInfo_2023-01-05.pdf]

Supplementary information

Synthesis, Characterization, and Photoactivation studies on the novel Pt(IV)-based [Pt($OCOCH_3$)$_3$(phterpy)] complex

Giovanni Canil, Juan Gurruchaga-Pereda, Simona Braccini, Lorella Marchetti, Tiziana Funaioli, Fabio Marchetti, Alessandro Pratesi, Luca Salassa, and Chiara Gabbiani,**

CONTENTS

1. **Different Behaviour of [PtI(phterpy)][CF_3SO_3] and [PtCl(phterpy)][CF_3SO_3] in the reaction with Silver Triflate, $AgCF_3SO_3$**
2. **Mixture of species obtained after oxidation with hydrogen peroxide**
3. **Characterization of complexes 3, 2, 1**
4. **X-Ray structure of compounds 3 and 2**
5. **Direct oxidation from compound 3 to 1**
6. **Stability studies in the dark**
7. **Irradiation of the Pt(IV) compound 1**
8. **Photocatalysis studies**
9. **Electrochemistry**
10. **Interaction with biomolecules**

1 Different Behaviour of [PtI(phterpy)][CF_3SO_3] and [PtCl(phterpy)][CF_3SO_3] in the reaction with Silver Triflate, $AgCF_3SO_3$.

During the study of its solution behavior, we found that [PtI(phterpy)][CF_3SO_3] reacted to a complete extent with the silver salt $AgCF_3SO_3$ in the solvent DMSO (Figures S1, S2). Both the ^{1}H NMR and the ^{195}Pt NMR spectra indicate the presence of a new species, with no detection of the starting material. On the other hand, the analog compound with a chloride in place of the iodide, [PtCl(phterpy)][CF_3SO_3], reacted only to a very limited extent with $AgCF_3SO_3$, most likely due to the difference in K_{ps} constants of AgCl and AgI (Figure S3). In this latter case the ^{195}Pt NMR is not shown because the resonance of the new species was not detected, probably due to insufficient concentration.

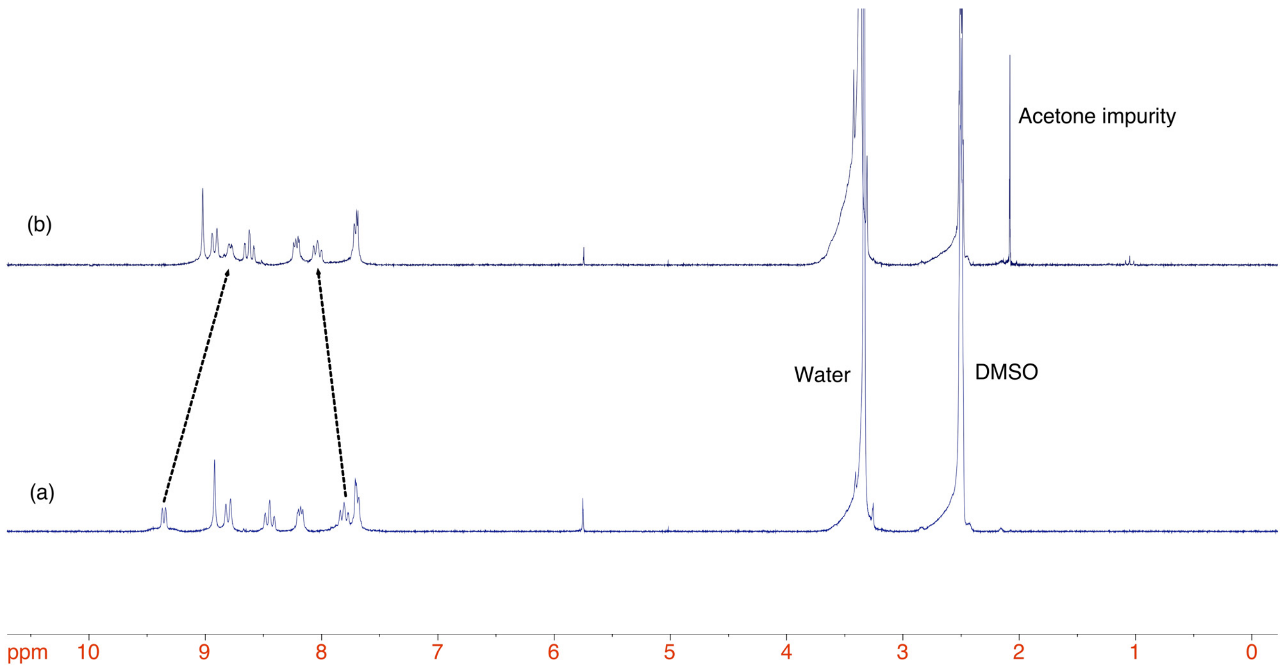

Figure S1. The comparison between the ^{1}H NMR of **3** (a) and the ^{1}H NMR of **3** treated with $AgCF_3SO_3$ (b) both in DMSO-*d*$_6$.

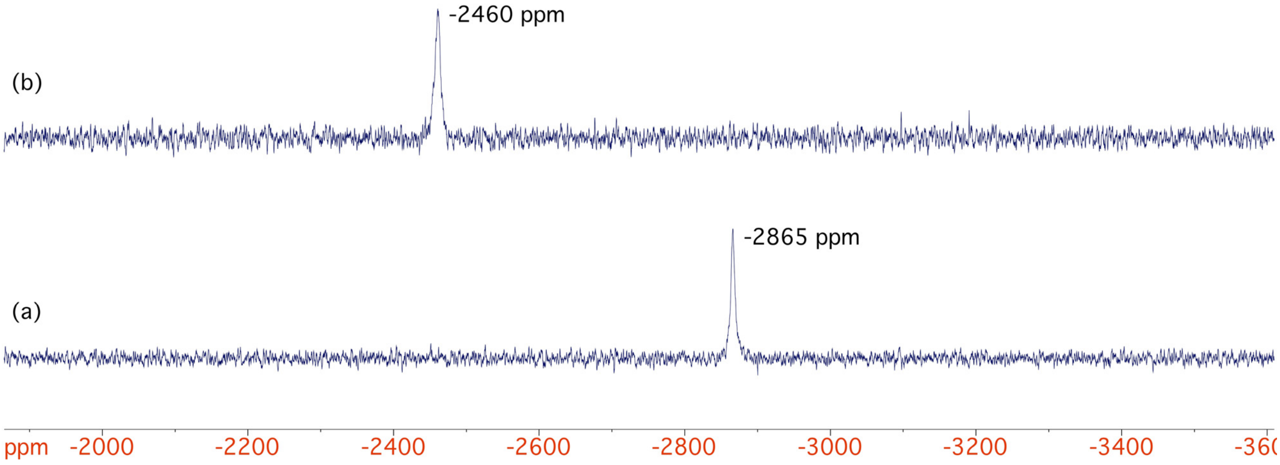

Figure S2. The comparison between the ^{195}Pt NMR of **3** (a) and the ^{195}Pt NMR of **3** treated with $AgCF_3SO_3$ (b) both in DMSO-d_6.

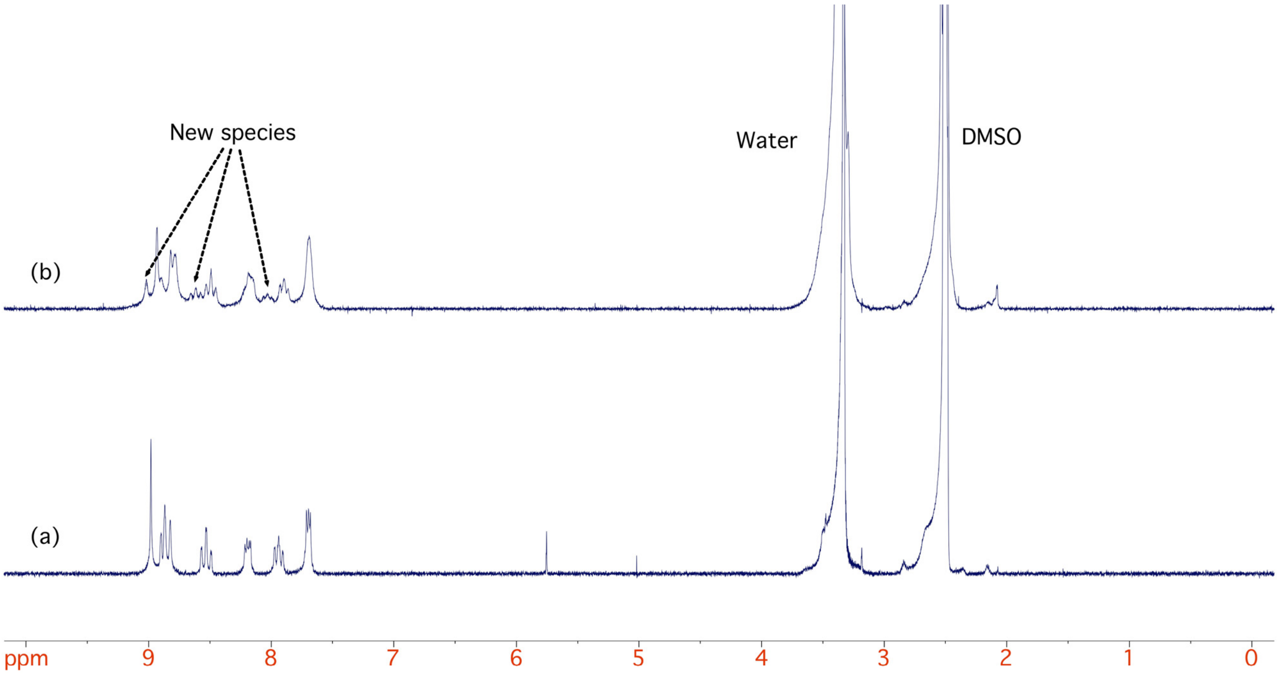

Figure S3. The comparison between the ^1H NMR spectra of pure $[PtCl(phterpy)][CF_3SO_3]$ (a) and the ^1H NMR of $[PtCl(phterpy)][CF_3SO_3]$ treated with $AgCF_3SO_3$ (b) both in DMSO-d_6.

2. ***Mixture of species obtained after oxidation with hydrogen peroxide***

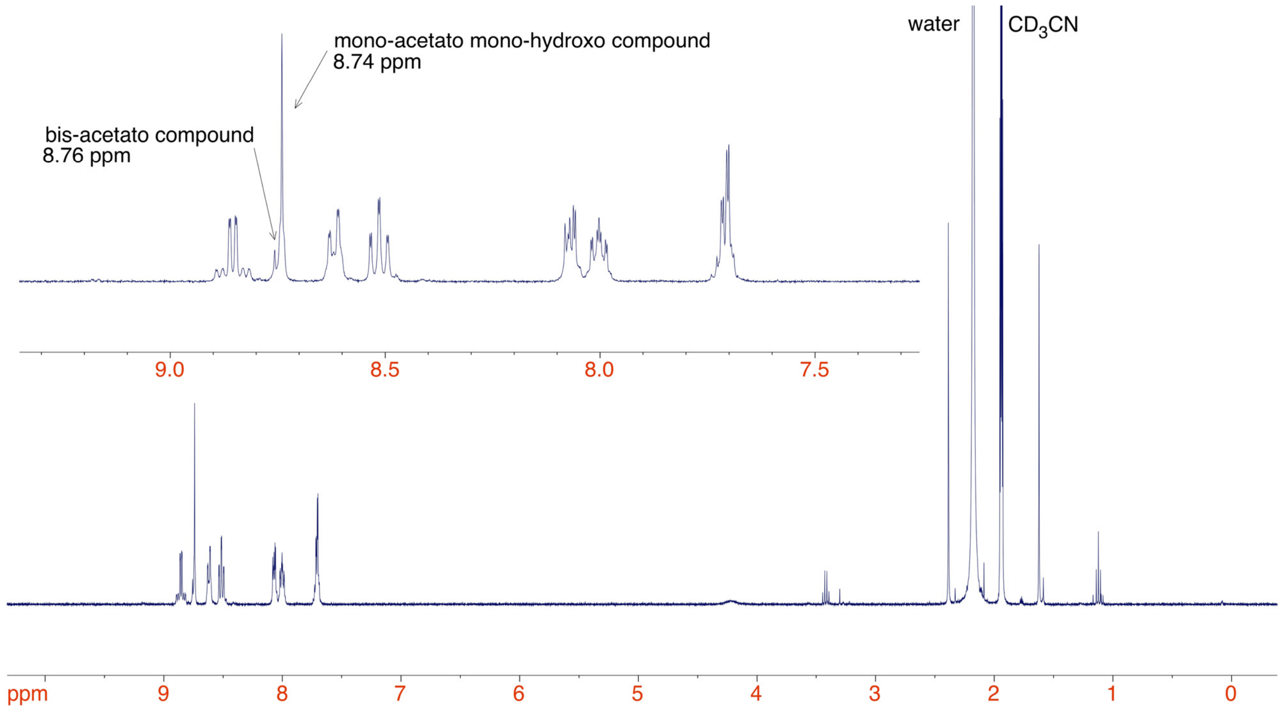

Figure S4. ^{1}H NMR spectrum of the mixture of Pt(IV) species obtained after oxidation with hydrogen peroxide in acetic acid. The solvent is CD_3CN, in the inset is shown the enlarged aromatic portion of the spectrum.

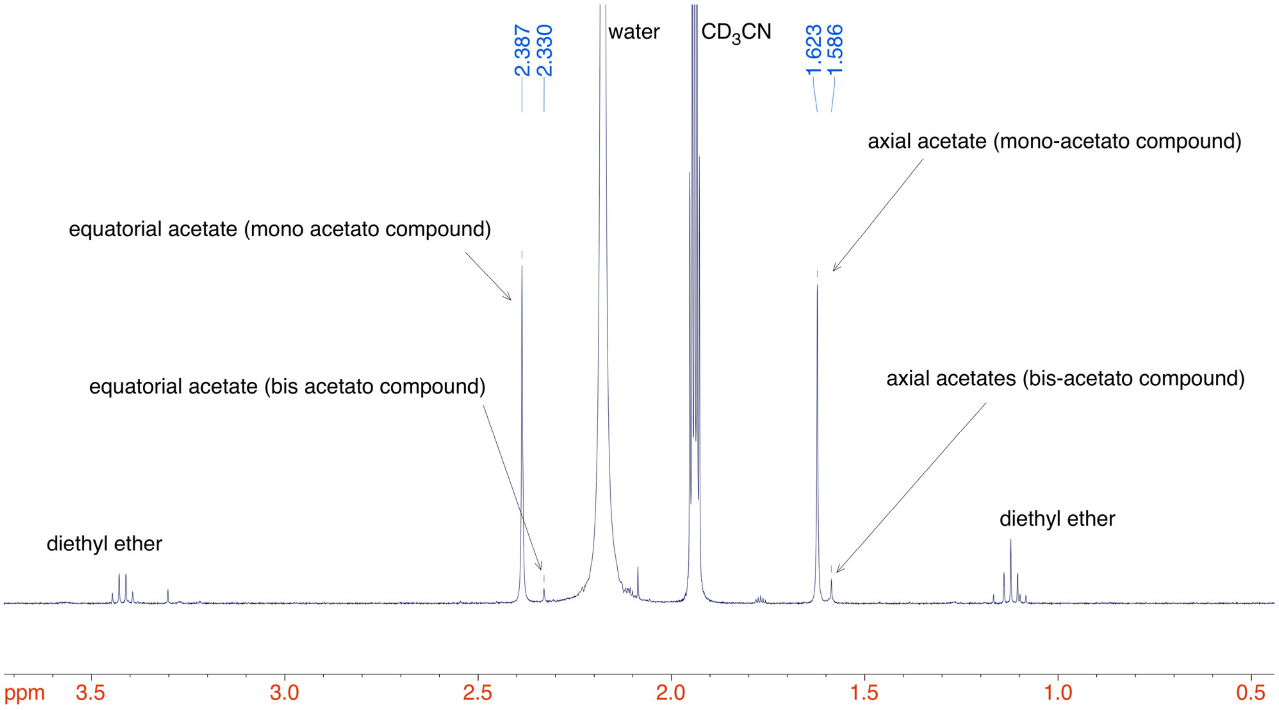

Figure S5. ^{1}H NMR spectrum in CD_3CN of the mixture of Pt(IV) species obtained after oxidation with hydrogen peroxide in acetic acid, enlarged aliphatic portion of the spectrum.

3. *Characterization of complexes 3, 2, 1.*

The ^{1}H NMR spectrum of **3** (Figure S4) shows only the peaks due to the phterpy ligand in the aromatic portion of the spectrum. Its ^{195}Pt NMR resonance fell at -2866 ppm (Figure S5), slightly upfield with respect to [PtCl(phterpy)][CF_3SO_3] (^{195}Pt NMR resonance at -2698 ppm), which is a common behavior shifting from a chloro to an iodo analog. Also, the shape of the signal resulted to be much broader with respect to the [PtI_2(COD)] starting material, due to the coordination of three quadrupolar nuclei, ^{14}N, to the platinum metal centre (this feature affected all the new compounds synthesized in this work). The precipitation of the iodide from **3** and the coordination of the acetate in **2** produced the expected signal of the methyl protons in the ^{1}H NMR spectrum, found at 2.10 ppm (Figure S6, S7). In the ^{13}C NMR (Figure S8) we found two new signals, at 23.32 ppm (methyl) and at 176.12 ppm (carbonyl). The ^{195}Pt NMR resonance shifted to -2405 ppm (Figure S9), a value which is downfield with respect of the starting material. Also, the IR spectrum of this compound showed a new stretching band at 1640 cm^{-1} (Figure S10), typical for carbonyl stretching. Ongoing from **2** to **1**, the ^{195}Pt NMR resonance moved largely downfield, at 1293 ppm (Figure S11). Moreover, a new signal due to the axial acetate groups was found in the ^{1}H NMR spectrum, at 1.59 ppm (in CD_3CN) and integrating for 6 protons (Figure S12). As expected, the two axial acetate display the same resonance in the ^{1}H and in the ^{13}C NMR spectra (Figure S13), due to their symmetric position. Finally, the oxidation from Pt(II) to Pt(IV) slightly shifts the resonances of all

the protons downfield (Figure S14 shows an enlarged area of the aromatic protons in CD3CN), due to the increased electronegativity of the Pt(IV) centre. The IR spectrum of compound **1** shows multiple stretching signals for the newly coordinated axial acetates, falling in the region 1670 cm^{-1}, while the other portions of the spectrum are almost unchanged (Figure S16).

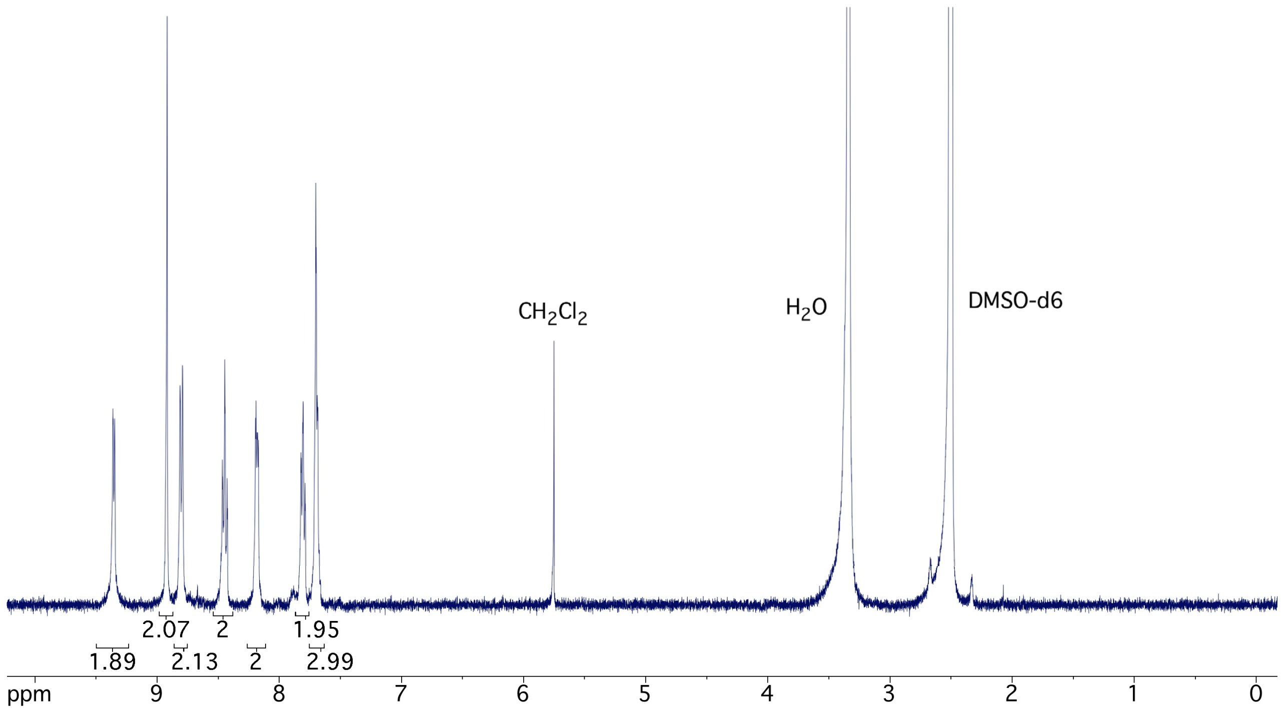

Figure S6. ^1H NMR spectrum of **3** in DMSO-d_6.

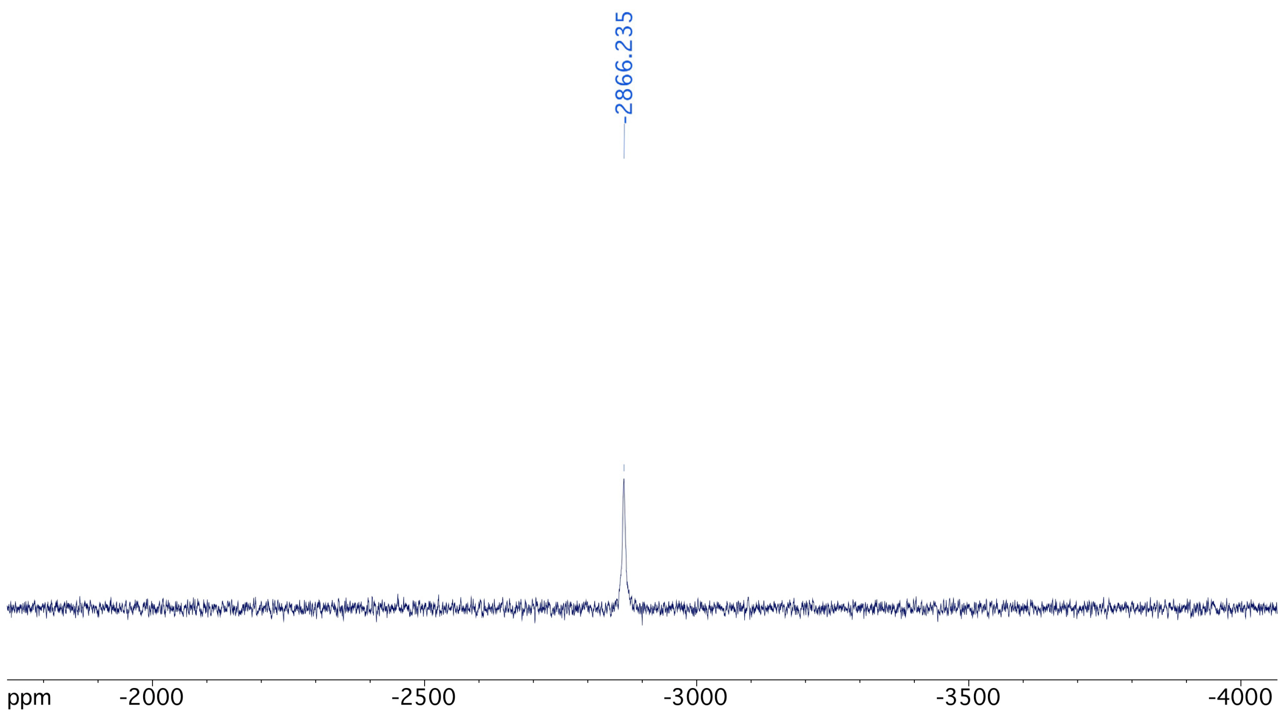

Figure S7. ^{195}Pt NMR spectrum of **3** in DMSO-*d*6.

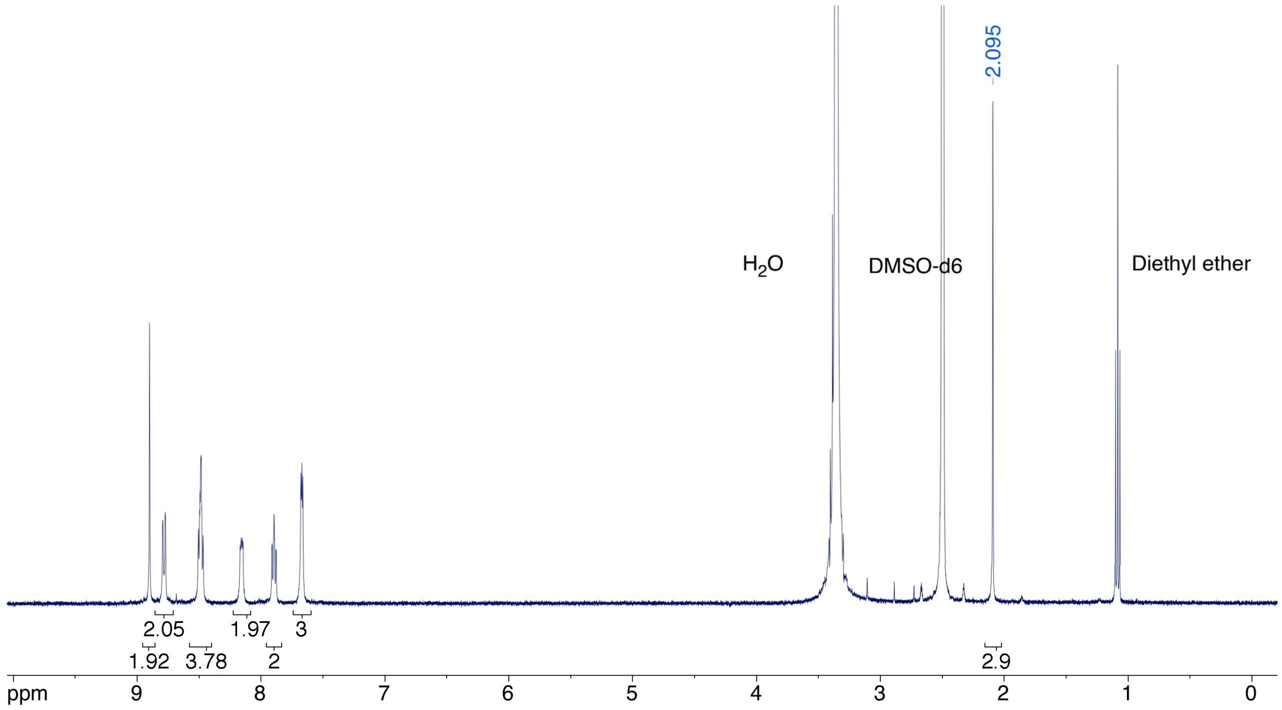

Figure S8. ^{1}H NMR spectrum of **2** in DMSO-*d*6.

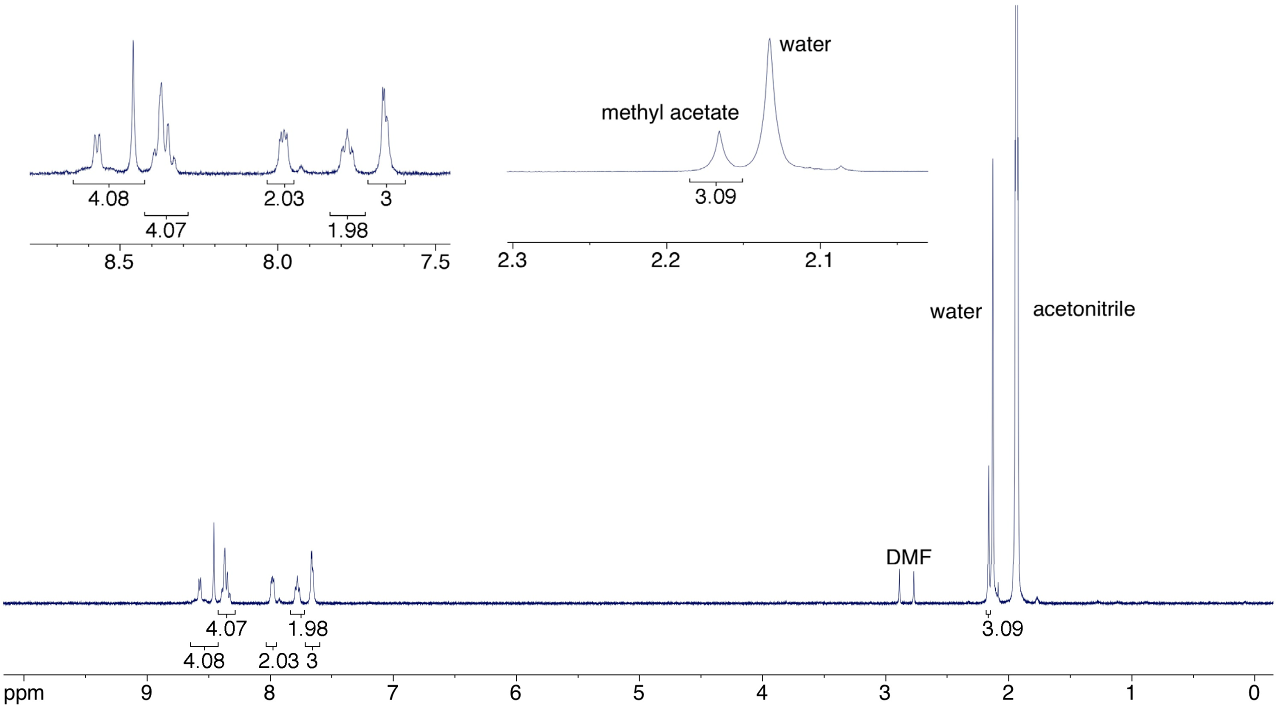

Figure S9. ^{1}H NMR spectrum of **2** in CD_3CN.

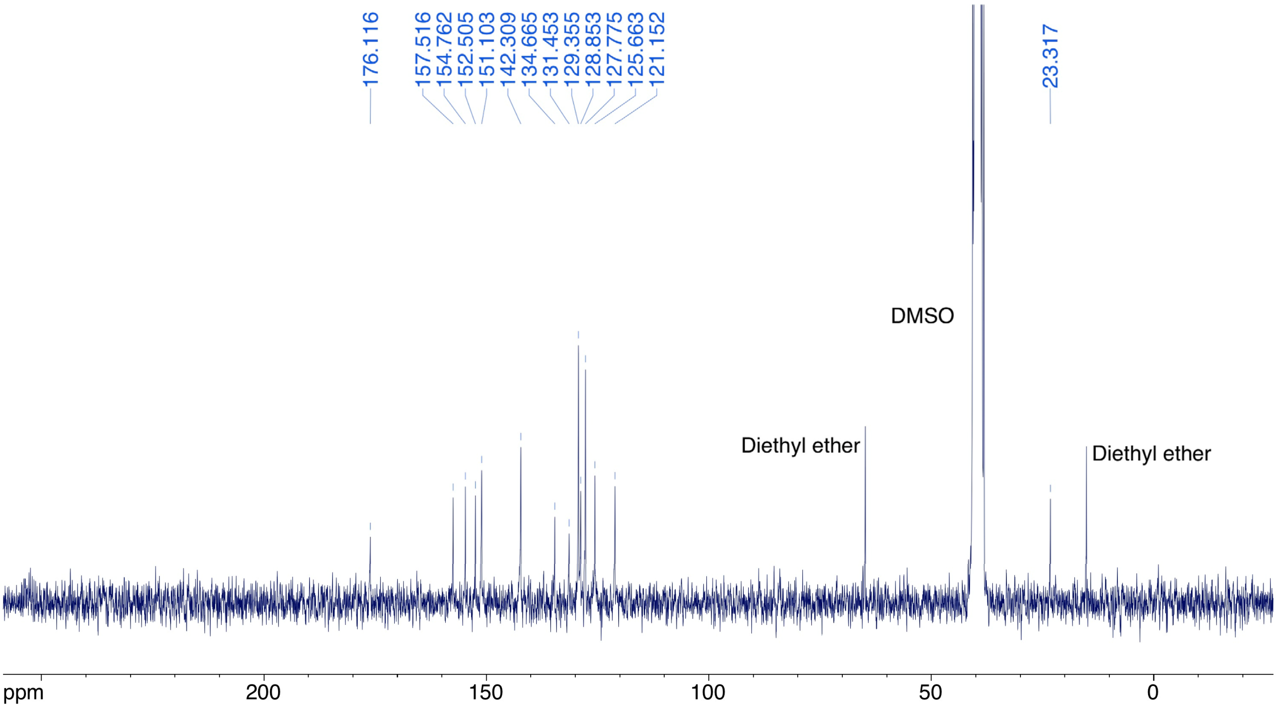

Figure S10. ^{13}C NMR spectrum of **2** in DMSO-d_6. It shows the 12 signals due to the phterpy aromatic rings (both tertiary and quaternary carbons) and the 2 carbons due to the acetate ligand, found at 23.32 ppm (methyl) and at 176.12 ppm (carbonyl C=O).

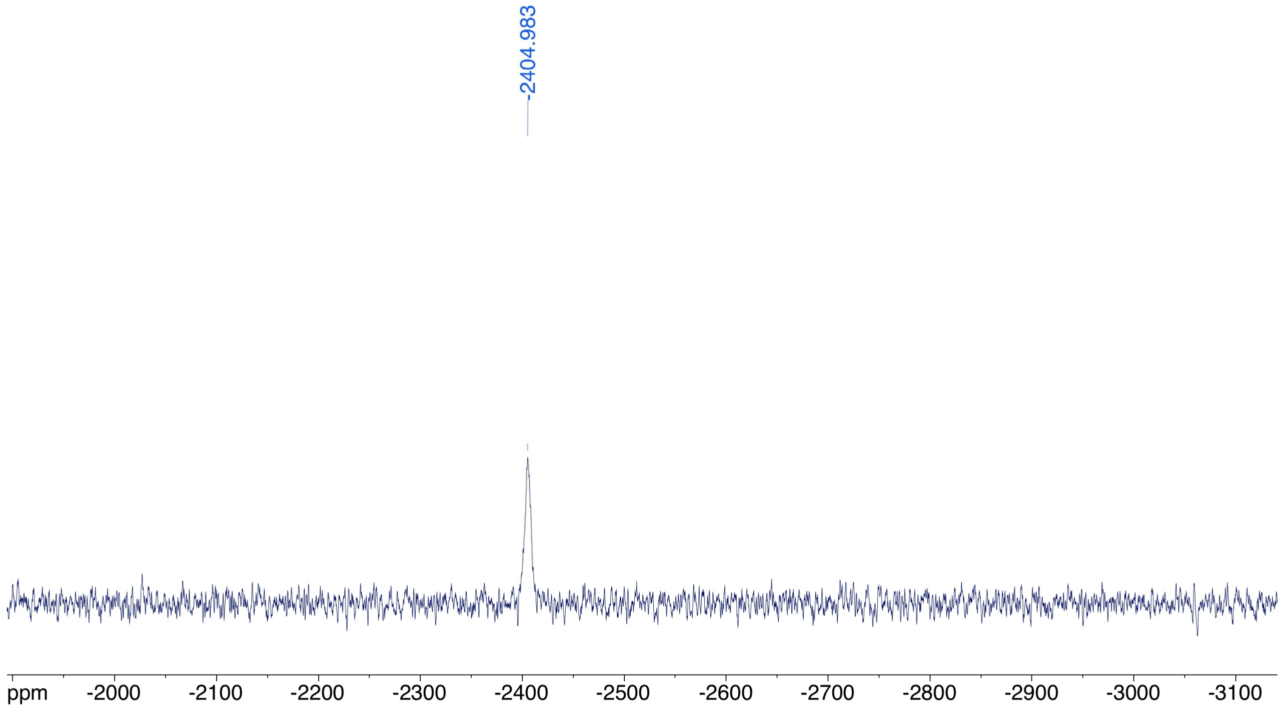

Figure S11. ^{195}Pt NMR spectrum of **2** in DMSO-*d*₆.

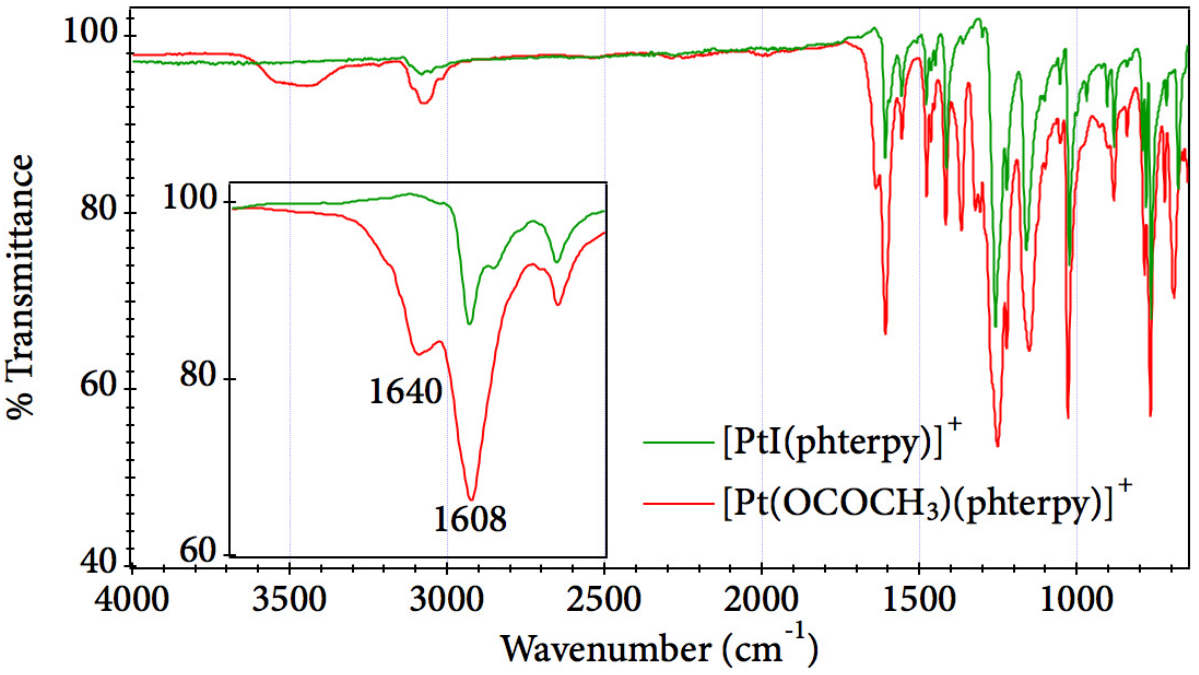

Figure S12. IR spectrum showing the differences between compounds **3** and **2**, with highlight in the region of the carbonyl stretching. The other portions of the spectrum are almost superimposable.

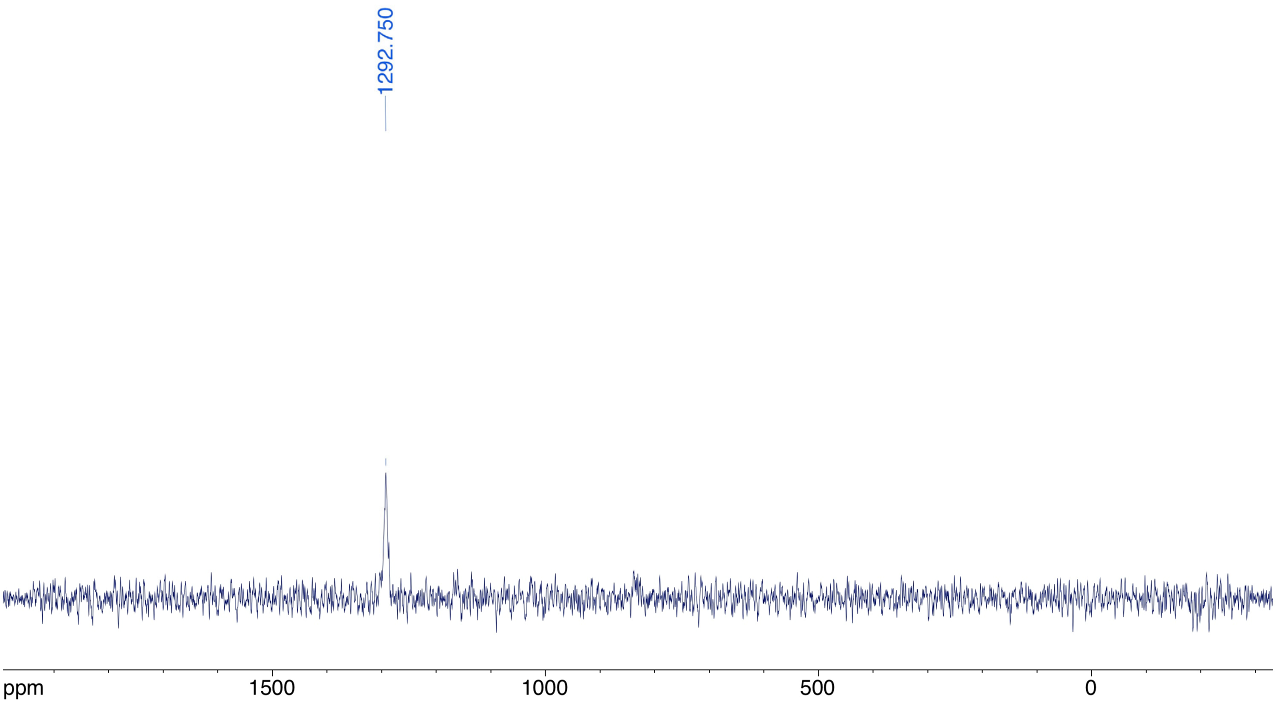

Figure S13. ^{195}Pt NMR spectrum of **1** in DMSO-d_6.

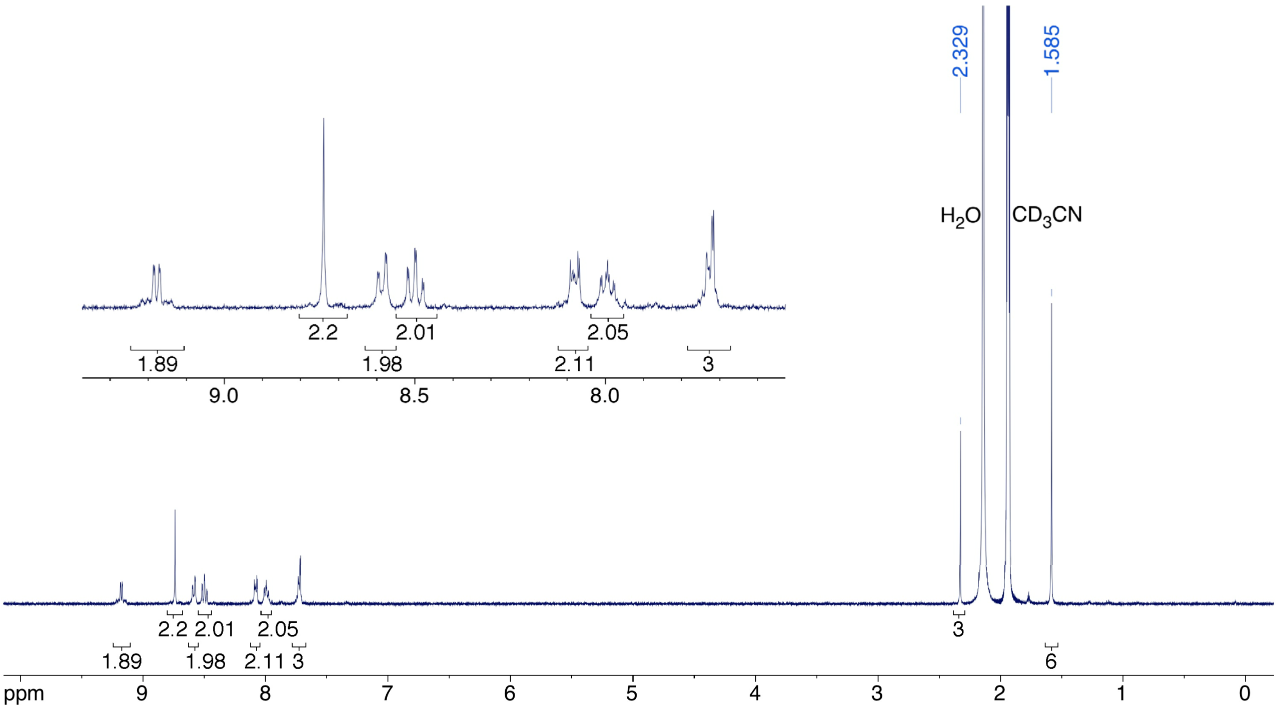

Figure S14. ^{1}H NMR spectrum of **1** in CD_3CN.

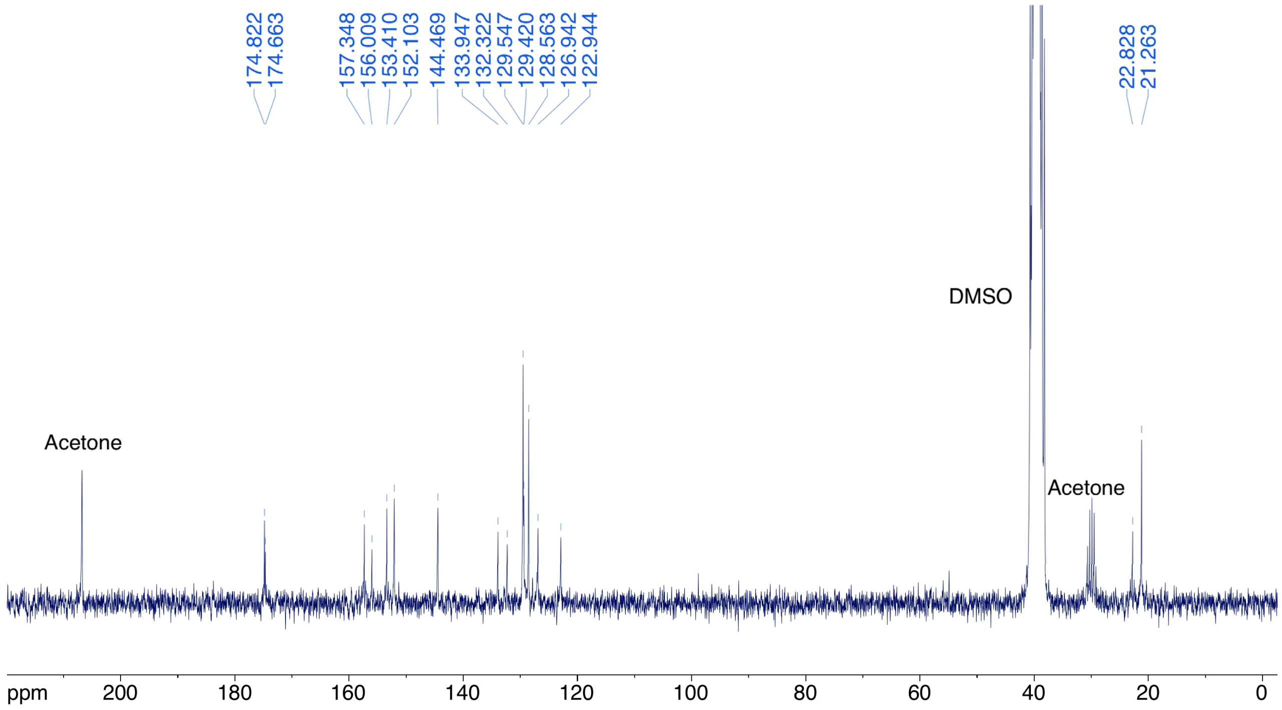

Figure S15. ^{13}C NMR spectrum of **1** in DMSO-d_6. It shows the 12 signals due to the phterpy aromatic rings (both tertiary and quaternary carbons) and the 4 carbons due to the acetate ligand, found at 22.83 ppm (equatorial methyl), 21.26 (axial methyls) 174.66 ppm (equatorial carbonyl) and at 174.82 (axial carbonyl).

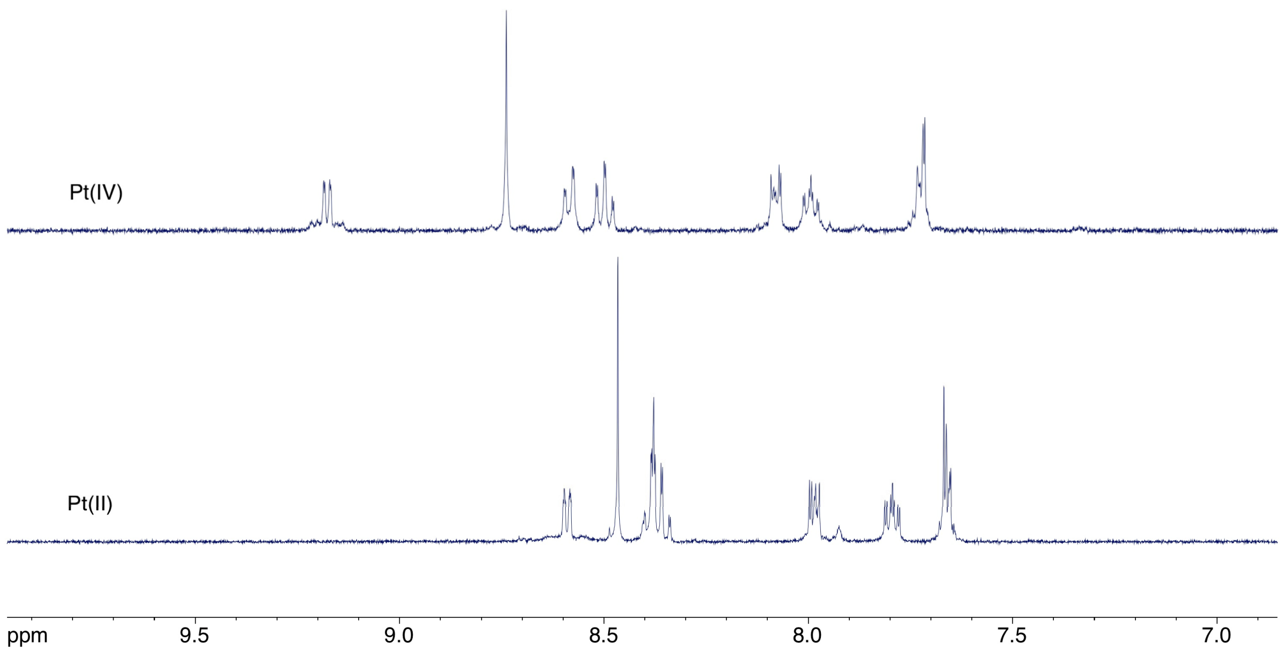

Figure S16. ^{1}H NMR spectra of compounds **2** (PtII) and **1** (PtIV) in the solvent CD_3CN, enlarged aromatic portion of the spectrum.

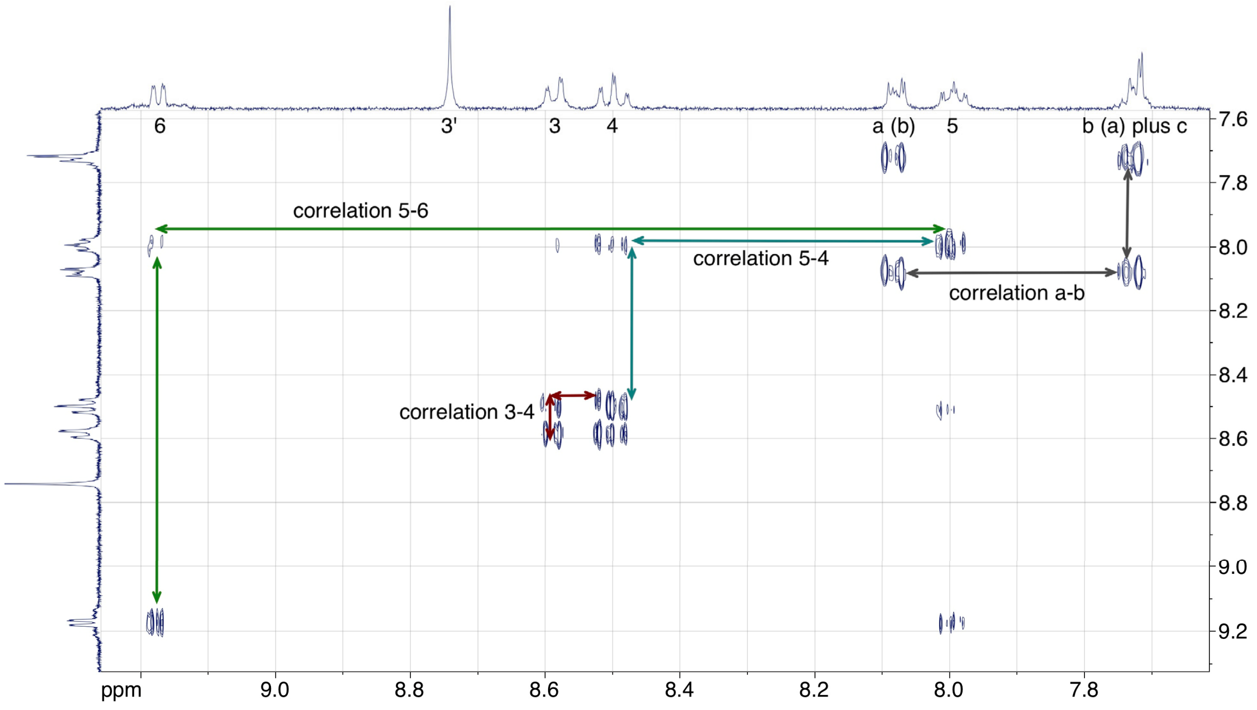

Figure S17. 2D ^{1}H NMR spectra of compound **1** in CD_3CN, enlarged aromatic portion showing the correlations between the protons. For the numeration scheme see Scheme 1 in the main text.

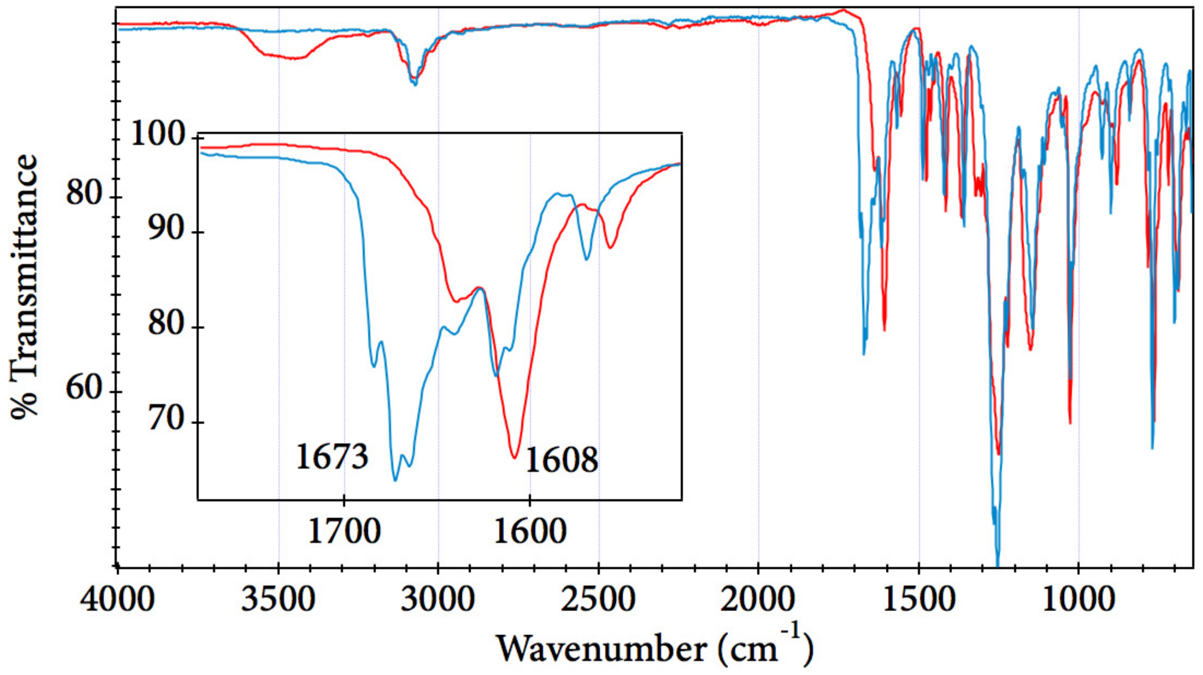

Figure S18. IR spectrum showing the differences between compounds **2** and **1**, with highlight in the region of the carbonyl stretching. The other portions of the spectrum are almost superimposable.

4. ***X-Ray structure of compounds 3 and 2.***

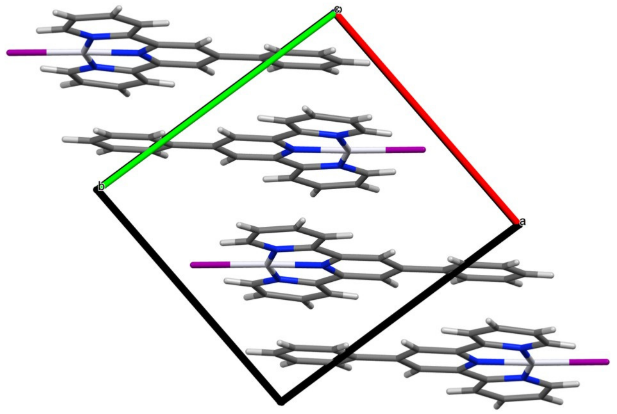

Figure S19. Unit cell in the c direction, showing the stacking of the cations for compound **3**.

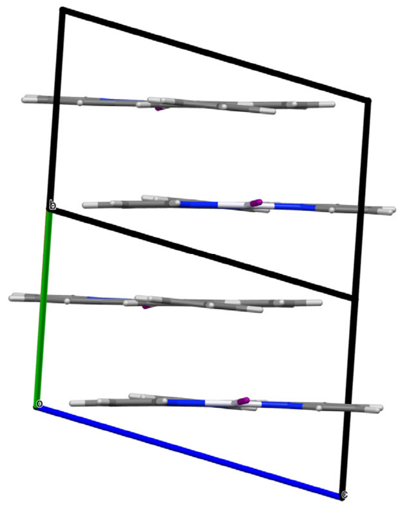

Figure S20. Along the [1 $\overline{1}$ 0] direction of the staking of the cations for compound **3**.

Table S1. Crystal data and structure refinement for compound **3**. All the structure solutions were found using the automated direct methods contained in SHELXS-97 program.

Identification code	shelx
Empirical formula	C22 H15 F3 I N3 O3 Pt S

Formula weight	780.42	
Temperature	296(2) K	
Wavelength	0.71073 Å	
Crystal system	Triclinic	
Space group	P -1	
Unit cell dimensions	a = 9.7976(4) Å	α= 104.6670(10)°.
	b = 10.6848(4) Å	β= 93.2560(10)°.
	c = 11.3494(5) Å	γ = 93.1290(10)°.
Volume	1144.62(8) $Å^3$	
Z	2	
Density (calculated)	2.264 Mg/m^3	
Absorption coefficient	7.624 mm^{-1}	
F(000)	732	
Crystal size	0.480 x 0.150 x 0.055 mm^3	
Theta range for data collection	3.139 to 34.998°.	
Index ranges	-14<=h<=15, -17<=k<=17, -18<=l<=18	
Reflections collected	52729	
Independent reflections	9992 [R(int) = 0.0367]	
Completeness to theta = 25.242°	98.5 %	
Absorption correction	Semi-empirical from equivalents	
Max. and min. transmission	0.7473 and 0.2955	
Refinement method	Full-matrix least-squares on F^2	
Data / restraints / parameters	9992 / 0 / 307	
Goodness-of-fit on F^2	1.041	
Final R indices [I>2sigma(I)]	R1 = 0.0317, wR2 = 0.0764	
R indices (all data)	R1 = 0.0400, wR2 = 0.0815	
Extinction coefficient	n/a	
Largest diff. peak and hole	1.762 and -2.569 e.$Å^{-3}$	

Table S2. Selected bond lengths [Å] and angles [°] for compound **3**.

Pt(1)-N(2)	1.942(2)
Pt(1)-N(3)	2.025(3)
Pt(1)-N(1)	2.030(3)

Pt(1)-I(1)	2.5834(3)
N(2)-Pt(1)-N(3)	80.47(10)
N(2)-Pt(1)-N(1)	80.54(10)
N(3)-Pt(1)-N(1)	160.99(11)
N(2)-Pt(1)-I(1)	178.06(7)
N(3)-Pt(1)-I(1)	98.78(8)
N(1)-Pt(1)-I(1)	100.23(7)

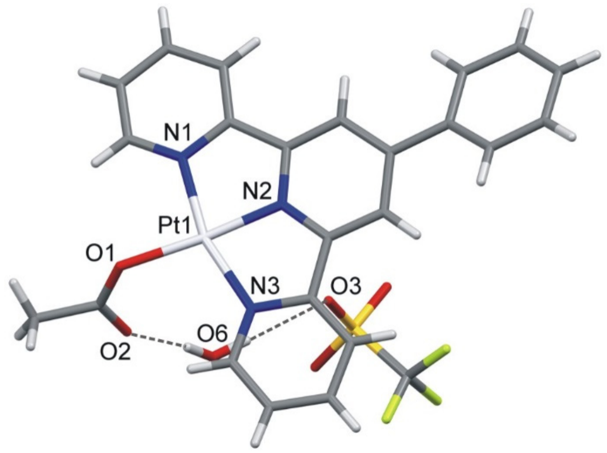

Figure S21. View of the asymmetrical unit in the crystal structure of **2**.

Table S3. Crystal data and structure refinement for compound **2**. All the structure solutions were found using the automated direct methods contained in SHELXS-97 program.

Identification code	shelx	
Empirical formula	C24 H20 F3 N3 O6 Pt S	
Formula weight	730.58	
Temperature	296(2) K	
Wavelength	0.71073 Å	
Crystal system	Monoclinic	
Space group	P 21/n	
Unit cell dimensions	a = 10.1509(3) Å	a= 90°.
	b = 15.2578(5) Å	b= 97.9700(10)°.
	c = 16.3529(5) Å	g = 90°.
Volume	2508.28(13) Å^3	
Z	4	

Density (calculated)	1.935 Mg/m^3
Absorption coefficient	5.746 mm^{-1}
F(000)	1416
Crystal size	0.350 x 0.170 x 0.140 mm^3
Theta range for data collection	2.859 to 30.586°.
Index ranges	-14<=h<=14, -21<=k<=21, -23<=l<=23
Reflections collected	41208
Independent reflections	7617 [R(int) = 0.0303]
Completeness to theta = 25.242°	98.3 %
Absorption correction	Semi-empirical from equivalents
Max. and min. transmission	0.7461 and 0.4600
Refinement method	Full-matrix least-squares on F^2
Data / restraints / parameters	7617 / 0 / 347
Goodness-of-fit on F^2	1.078
Final R indices [I>2sigma(I)]	R1 = 0.0243, wR2 = 0.0579
R indices (all data)	R1 = 0.0276, wR2 = 0.0595
Extinction coefficient	n/a
Largest diff. peak and hole	1.418 and -1.177 e.Å^{-3}

Table S4. Bond lengths [Å] and angles [°] for compound **2**.

Pt(1)-N(2)	1.926(2)
Pt(1)-N(1)	2.012(2)
Pt(1)-N(3)	2.023(2)
Pt(1)-O(1)	2.030(2)
O(1)-C(1)	1.246(4)
O(2)-C(1)	1.206(4)
C(1)-C(2)	1.516(5)
C(2)-H(2A)	0.9600
C(2)-H(2B)	0.9600
C(2)-H(2C)	0.9600
N(1)-C(3)	1.344(3)
N(1)-C(7)	1.372(3)
C(3)-C(4)	1.375(5)

C(3)-H(3)	0.9300
C(4)-C(5)	1.374(5)
C(4)-H(4)	0.9300
C(5)-C(6)	1.391(4)
C(5)-H(5)	0.9300
C(6)-C(7)	1.379(4)
C(6)-H(6)	0.9300
C(7)-C(8)	1.473(3)
C(8)-N(2)	1.347(3)
C(8)-C(9)	1.385(3)
N(2)-C(12)	1.343(3)
C(9)-C(10)	1.399(4)
C(9)-H(9)	0.9300
C(10)-C(11)	1.404(4)
C(10)-C(18)	1.484(3)
C(11)-C(12)	1.380(3)
C(11)-H(11)	0.9300
C(12)-C(13)	1.481(4)
C(13)-N(3)	1.372(3)
C(13)-C(14)	1.381(4)
N(3)-C(17)	1.342(4)
C(14)-C(15)	1.393(4)
C(14)-H(14)	0.9300
C(15)-C(16)	1.380(5)
C(15)-H(15)	0.9300
C(16)-C(17)	1.378(5)
C(16)-H(16)	0.9300
C(17)-H(17)	0.9300
C(18)-C(19)	1.394(4)
C(18)-C(23)	1.395(4)
C(19)-C(20)	1.397(4)
C(19)-H(19)	0.9300
C(20)-C(21)	1.363(6)
C(20)-H(20)	0.9300
C(21)-C(22)	1.367(6)

C(21)-H(21)	0.9300
C(22)-C(23)	1.382(5)
C(22)-H(22)	0.9300
C(23)-H(23)	0.9300
S(1)-O(3)	1.414(3)
S(1)-O(5)	1.428(3)
S(1)-O(4)	1.435(3)
S(1)-C(24)	1.783(6)
C(24)-F(3)	1.253(8)
C(24)-F(1)	1.292(7)
C(24)-F(2)	1.410(11)
O(6)-H(6A)	0.8441
O(6)-H(6B)	0.8496

N(2)-Pt(1)-N(1)	81.41(9)
N(2)-Pt(1)-N(3)	81.00(9)
N(1)-Pt(1)-N(3)	162.29(9)
N(2)-Pt(1)-O(1)	174.61(9)
N(1)-Pt(1)-O(1)	95.30(10)
N(3)-Pt(1)-O(1)	102.40(10)
C(1)-O(1)-Pt(1)	120.06(19)
O(2)-C(1)-O(1)	125.7(3)
O(2)-C(1)-C(2)	119.0(3)
O(1)-C(1)-C(2)	115.2(3)
C(1)-C(2)-H(2A)	109.5
C(1)-C(2)-H(2B)	109.5
H(2A)-C(2)-H(2B)	109.5
C(1)-C(2)-H(2C)	109.5
H(2A)-C(2)-H(2C)	109.5
H(2B)-C(2)-H(2C)	109.5
C(3)-N(1)-C(7)	119.3(3)
C(3)-N(1)-Pt(1)	127.7(2)
C(7)-N(1)-Pt(1)	112.91(17)
N(1)-C(3)-C(4)	121.4(3)
N(1)-C(3)-H(3)	119.3

C(4)-C(3)-H(3)	119.3
C(5)-C(4)-C(3)	120.1(3)
C(5)-C(4)-H(4)	119.9
C(3)-C(4)-H(4)	119.9
C(4)-C(5)-C(6)	119.0(3)
C(4)-C(5)-H(5)	120.5
C(6)-C(5)-H(5)	120.5
C(7)-C(6)-C(5)	119.3(3)
C(7)-C(6)-H(6)	120.4
C(5)-C(6)-H(6)	120.4
N(1)-C(7)-C(6)	120.9(2)
N(1)-C(7)-C(8)	114.7(2)
C(6)-C(7)-C(8)	124.3(2)
N(2)-C(8)-C(9)	119.3(2)
N(2)-C(8)-C(7)	112.6(2)
C(9)-C(8)-C(7)	128.1(2)
C(12)-N(2)-C(8)	123.1(2)
C(12)-N(2)-Pt(1)	118.72(17)
C(8)-N(2)-Pt(1)	117.87(17)
C(8)-C(9)-C(10)	119.6(2)
C(8)-C(9)-H(9)	120.2
C(10)-C(9)-H(9)	120.2
C(9)-C(10)-C(11)	119.0(2)
C(9)-C(10)-C(18)	121.3(2)
C(11)-C(10)-C(18)	119.8(2)
C(12)-C(11)-C(10)	119.4(2)
C(12)-C(11)-H(11)	120.3
C(10)-C(11)-H(11)	120.3
N(2)-C(12)-C(11)	119.7(2)
N(2)-C(12)-C(13)	112.4(2)
C(11)-C(12)-C(13)	127.9(2)
N(3)-C(13)-C(14)	121.8(3)
N(3)-C(13)-C(12)	114.5(2)
C(14)-C(13)-C(12)	123.6(2)
C(17)-N(3)-C(13)	118.8(3)

C(17)-N(3)-Pt(1)	128.1(2)
C(13)-N(3)-Pt(1)	113.08(17)
C(13)-C(14)-C(15)	118.5(3)
C(13)-C(14)-H(14)	120.7
C(15)-C(14)-H(14)	120.7
C(16)-C(15)-C(14)	119.2(3)
C(16)-C(15)-H(15)	120.4
C(14)-C(15)-H(15)	120.4
C(17)-C(16)-C(15)	119.9(3)
C(17)-C(16)-H(16)	120.1
C(15)-C(16)-H(16)	120.1
N(3)-C(17)-C(16)	121.6(3)
N(3)-C(17)-H(17)	119.2
C(16)-C(17)-H(17)	119.2
C(19)-C(18)-C(23)	118.9(3)
C(19)-C(18)-C(10)	121.1(3)
C(23)-C(18)-C(10)	120.0(3)
C(18)-C(19)-C(20)	119.6(3)
C(18)-C(19)-H(19)	120.2
C(20)-C(19)-H(19)	120.2
C(21)-C(20)-C(19)	120.5(4)
C(21)-C(20)-H(20)	119.7
C(19)-C(20)-H(20)	119.7
C(20)-C(21)-C(22)	120.2(3)
C(20)-C(21)-H(21)	119.9
C(22)-C(21)-H(21)	119.9
C(21)-C(22)-C(23)	120.6(4)
C(21)-C(22)-H(22)	119.7
C(23)-C(22)-H(22)	119.7
C(22)-C(23)-C(18)	120.1(4)
C(22)-C(23)-H(23)	119.9
C(18)-C(23)-H(23)	119.9
O(3)-S(1)-O(5)	112.3(2)
O(3)-S(1)-O(4)	115.6(2)
O(5)-S(1)-O(4)	116.5(2)

O(3)-S(1)-C(24)	103.5(4)
O(5)-S(1)-C(24)	104.1(3)
O(4)-S(1)-C(24)	102.5(3)
F(3)-C(24)-F(1)	110.1(7)
F(3)-C(24)-F(2)	105.7(7)
F(1)-C(24)-F(2)	108.1(7)
F(3)-C(24)-S(1)	114.1(5)
F(1)-C(24)-S(1)	112.5(5)
F(2)-C(24)-S(1)	105.9(6)
H(6A)-O(6)-H(6B)	117.4

Detailed crystallographic data have been deposited in the form of CIF file with the Cambridge Crystallographic Data Center, deposition number 2002946 and 2121178, and can obtained free of charge at www.ccdc.cam.ac.uk/data_request/cif by quoting the present paper.

5. Direct oxidation from compound 3 to 1.

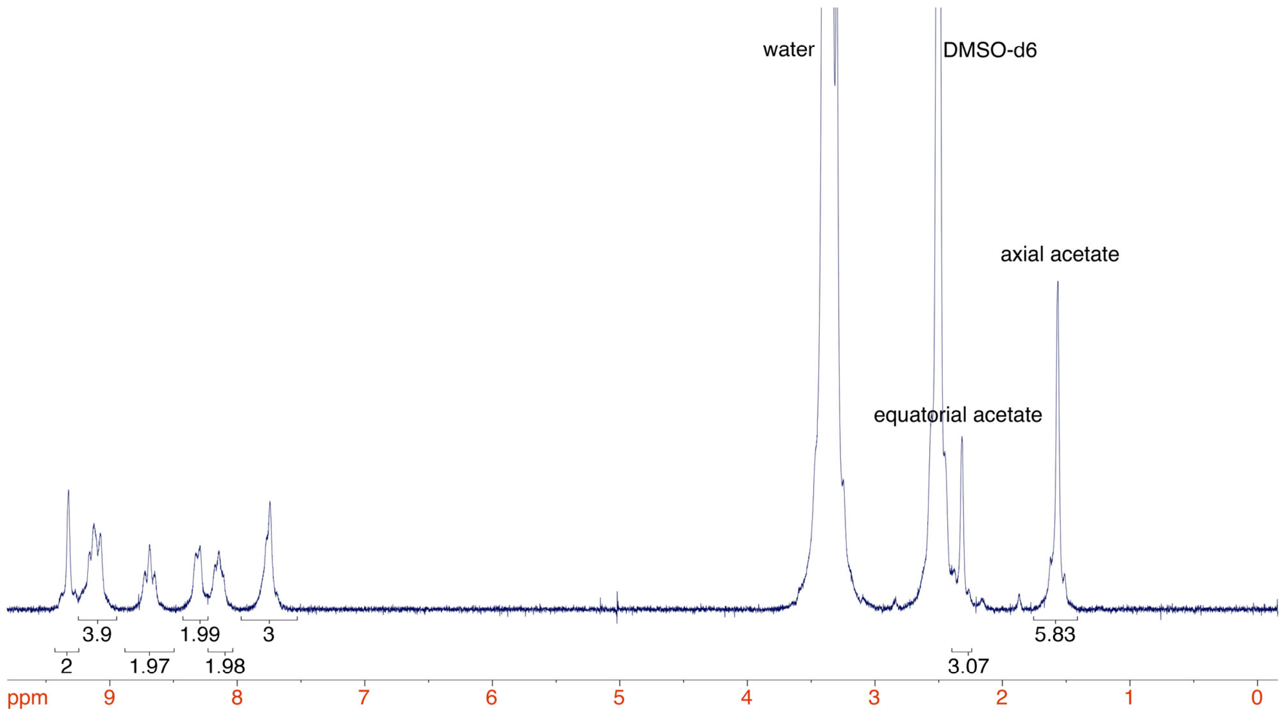

Figure S22. ^{1}H NMR spectrum in DMSO-d_6 of the Pt(IV) compound obtained after direct oxidation and carboxylation of **3**. The resonances and the integration correspond to those of compound **1**.

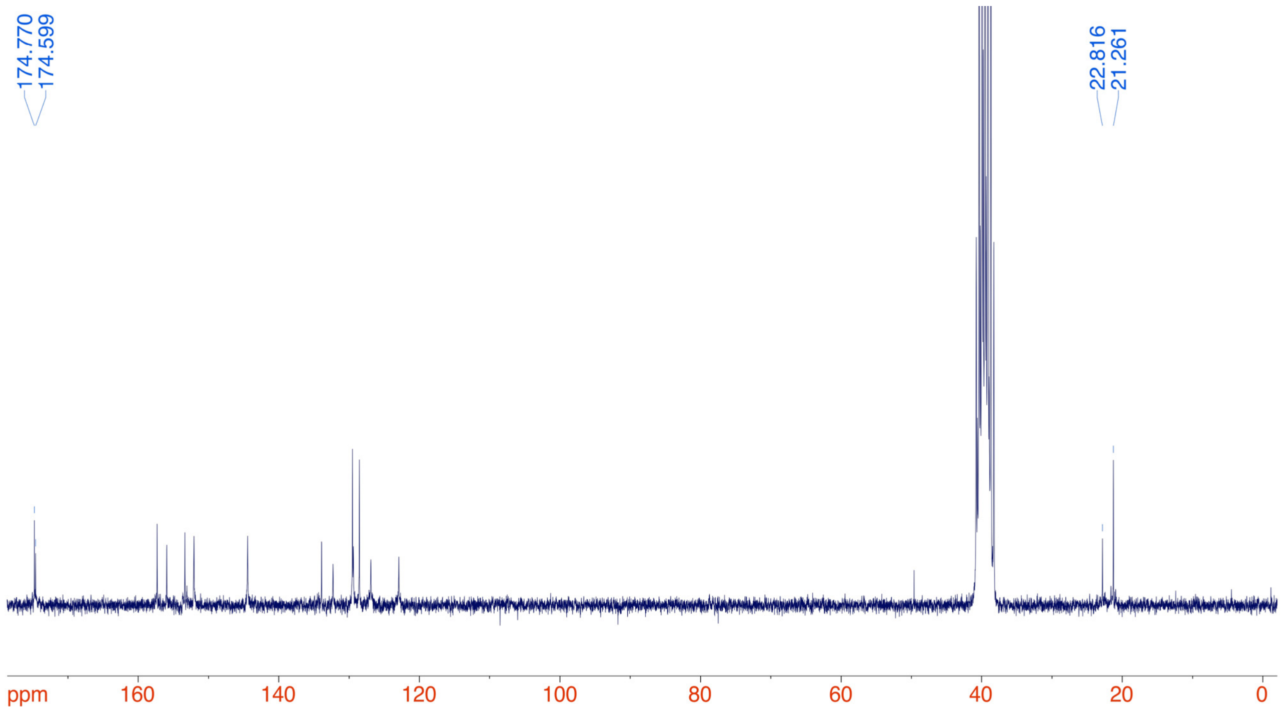

Figure S23. ^{13}C NMR spectrum in DMSO-d_6 of the Pt(IV) compound obtained after direct oxidation and carboxylation of **3**. The resonances of the aliphatic carbons, and those of the carbonyl carbons (highlighted in the picture) correspond to those of compound **1**.

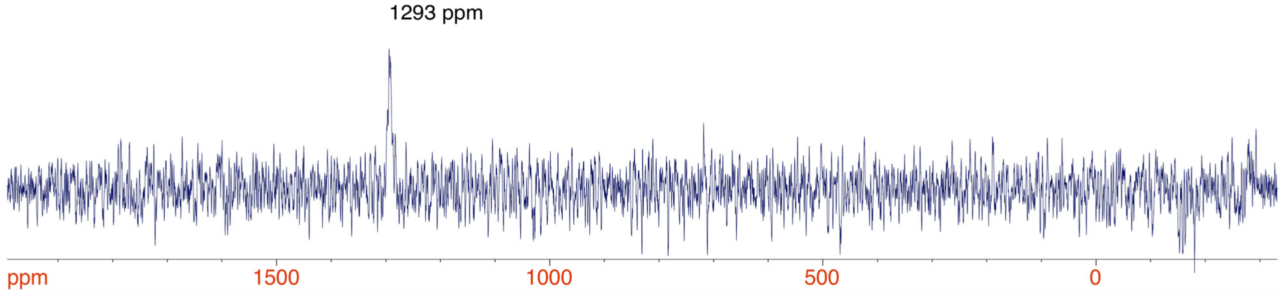

Figure S24. ^{195}Pt NMR spectrum in DMSO-d_6 of the Pt(IV) compound obtained after direct oxidation and carboxylation of **3**. The resonance of the platinum compound is the same as that of

compound **1**.

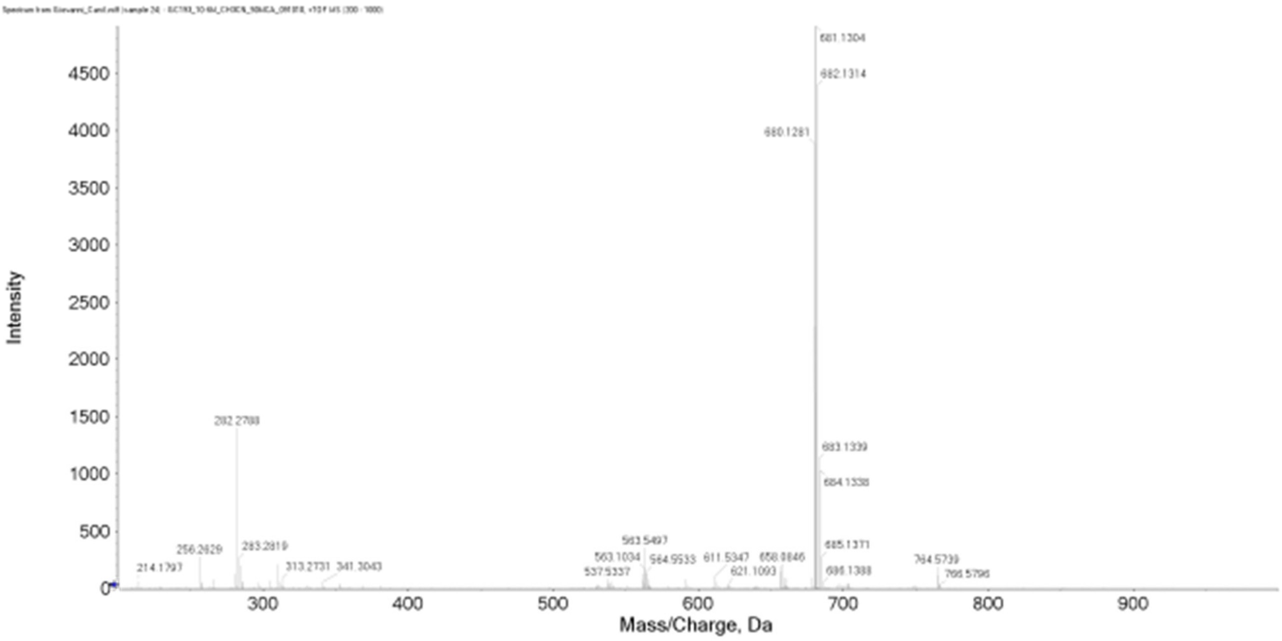

Figure S25. ESI mass spectrum of the Pt(IV) compound obtained after direct oxidation and carboxylation of **3**, full spectrum.

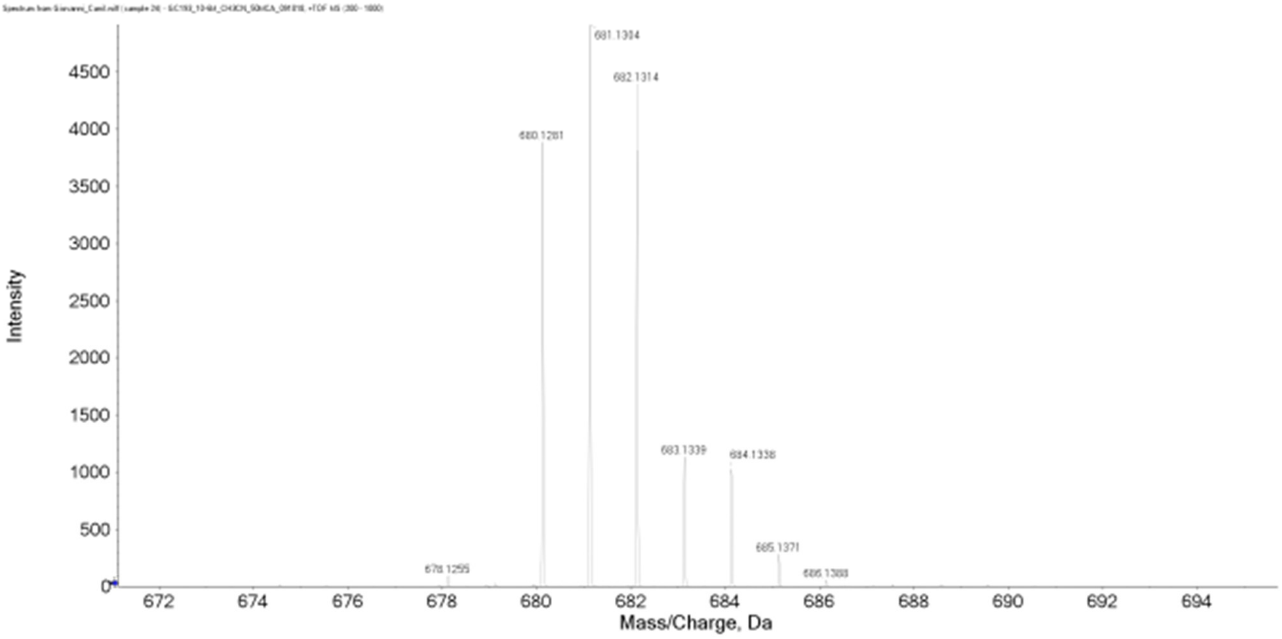

Figure S26. ESI mass spectrum of the Pt(IV) compound obtained after direct oxidation and carboxylation of **3**, zoom on the portion around the most intense peak, at 681.1304 Da. The fragment corresponds to the molecular weight of the cation $[Pt(OCOCH_3)_3(phterpy)]^+$ (681.13 Da,

exact mass), which corresponds to compound **1**.

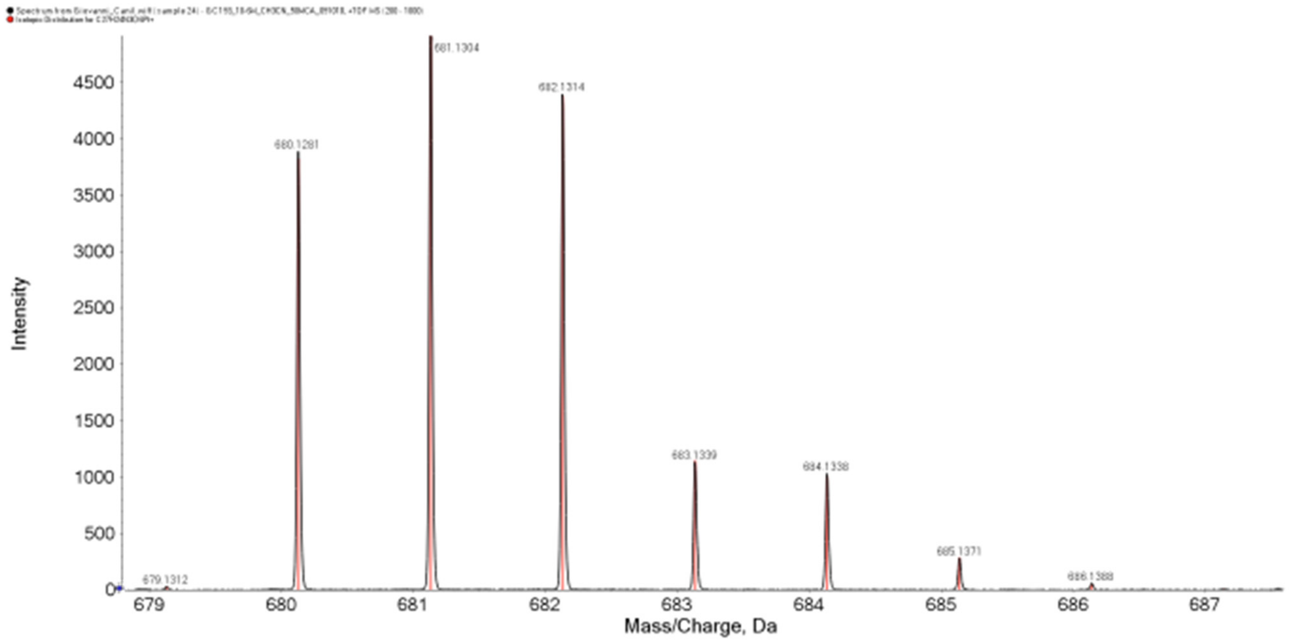

Figure S27. ESI mass spectrum of the Pt(IV) compound obtained after direct oxidation and carboxylation of **3**, zoom on the portion around the most intense peak, at 681.1304 Da and comparison with the theoretical mass spectrum.

6. *Stability studies in the dark.*

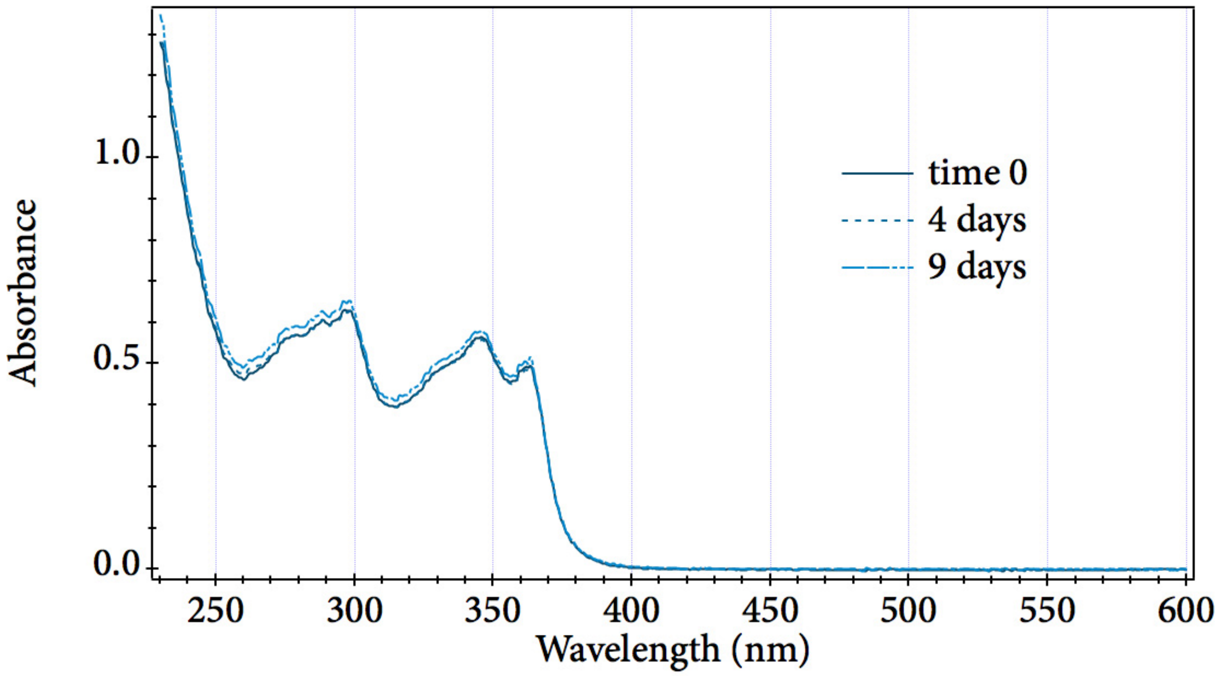

Figure S28. UV-Vis absorption spectra of **1** (3×10^{-5} M) in PB (10×10^{-3} M, pH=7.3). The spectra were recorded at different times, showing no appreciable change after 9 days.

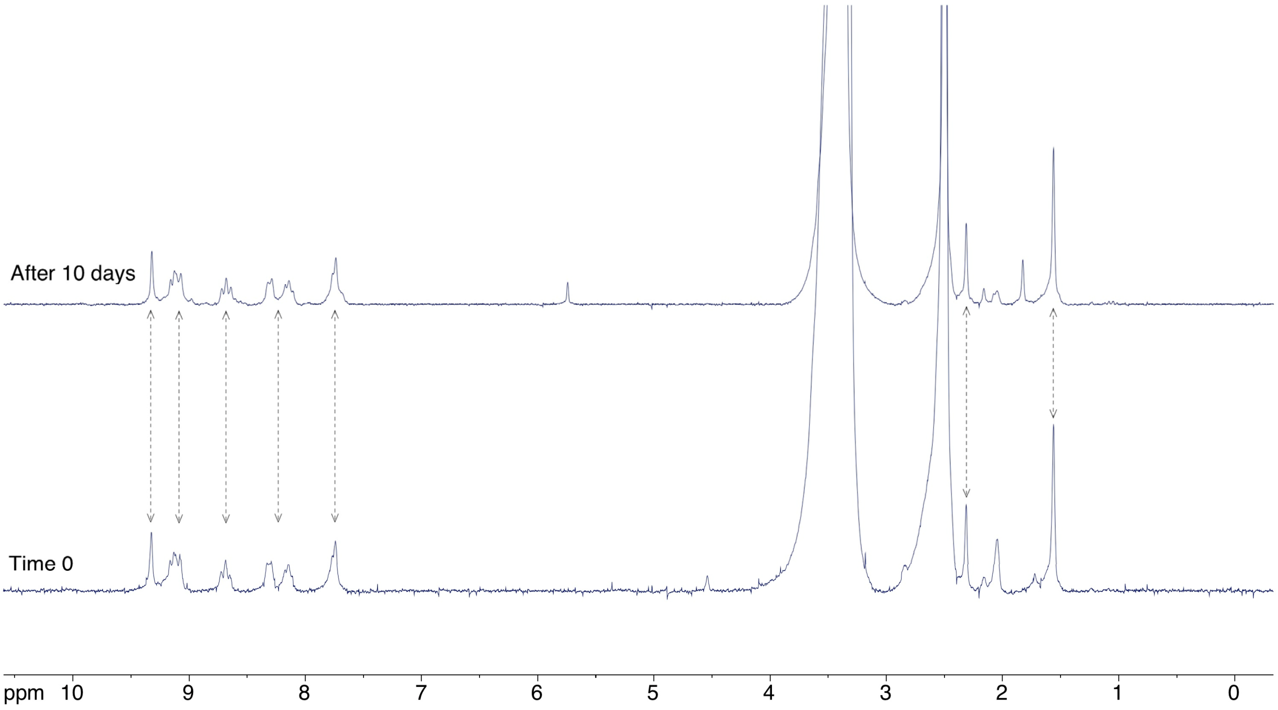

Figure S29. ^{1}H NMR spectra of **1** in DMSO-d_6 recorded at time 0 and after 10 days. The arrows indicate the diagnostic resonances that confirm the Pt(IV) oxidation state, with no appreciable peaks of the Pt(II) species.

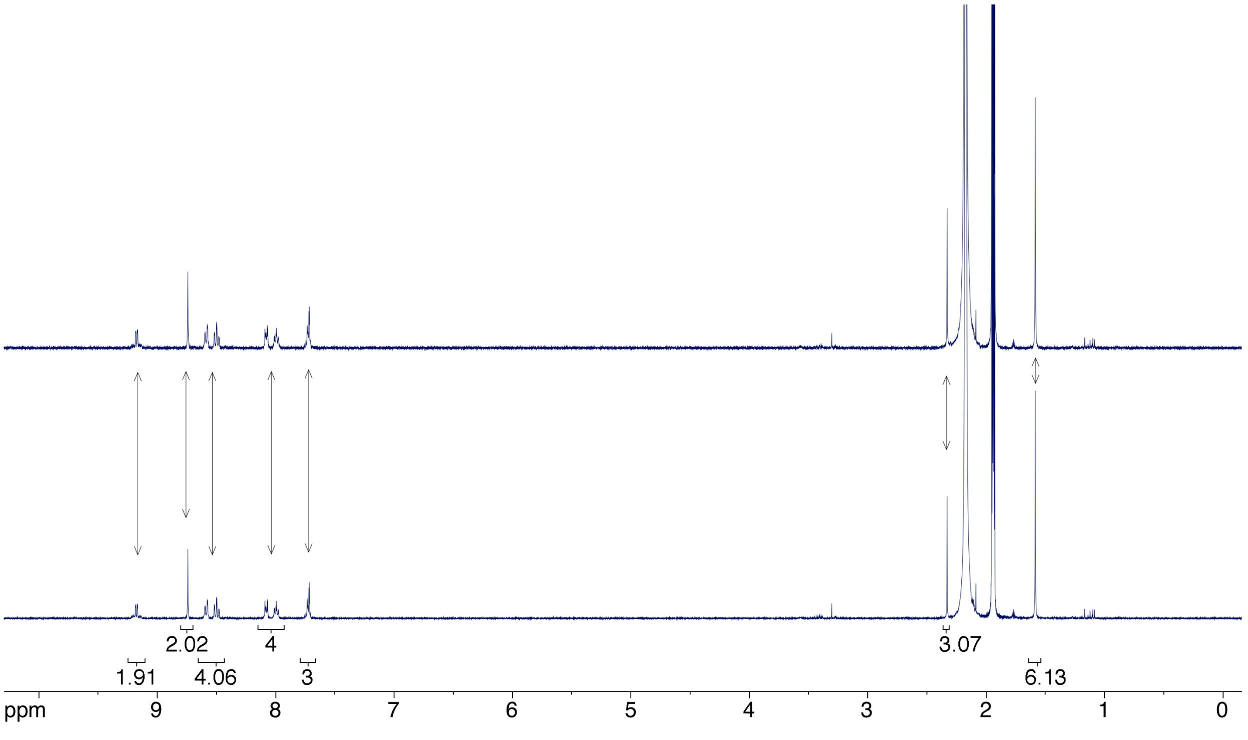

Figure S30. ^{1}H NMR spectra of **1** in CD_3CN recorded at time 0 and after one week. The arrows indicate the diagnostic resonances that confirm the Pt(IV) oxidation state, without any trace of the Pt(II) compound.

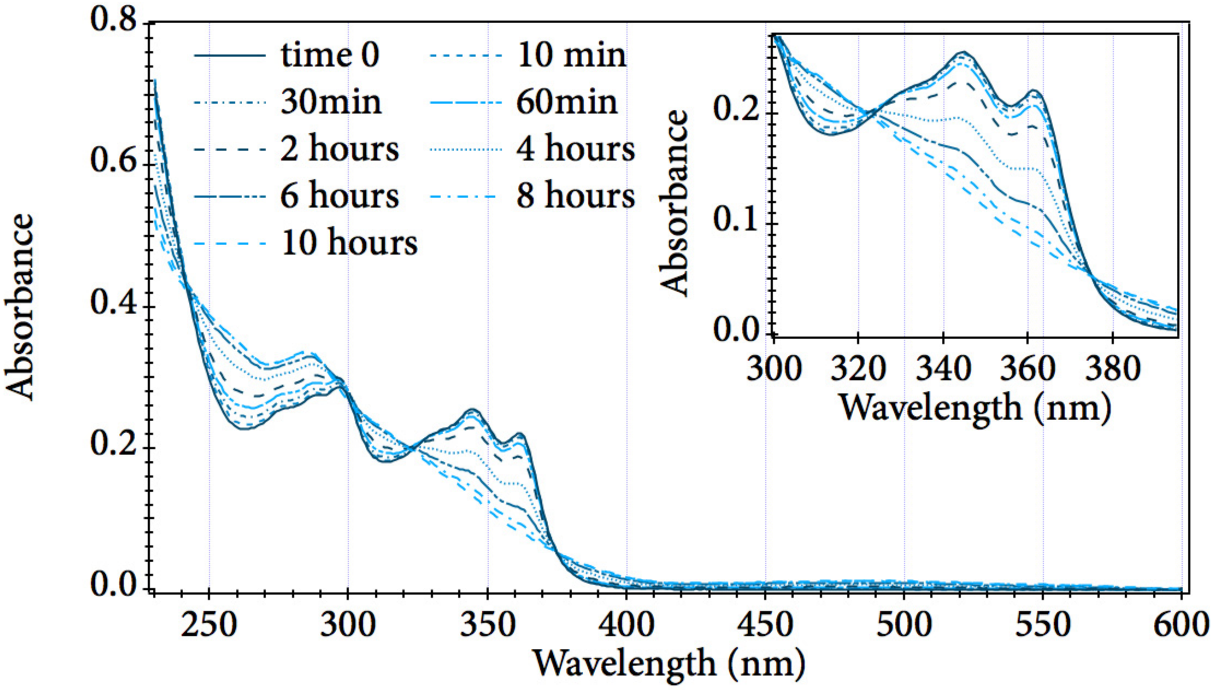

Figure S31. UV-Vis absorption spectra of **1** (1×10^{-5} M) in PB (50×10^{-3} M, pH=7.0) at 37 °C in the presence of GSH (5×10^{-3} M, 500 eq). The spectra were recorded at the times specified in the legend, showing the effect of GSH on the Pt(IV) compound.

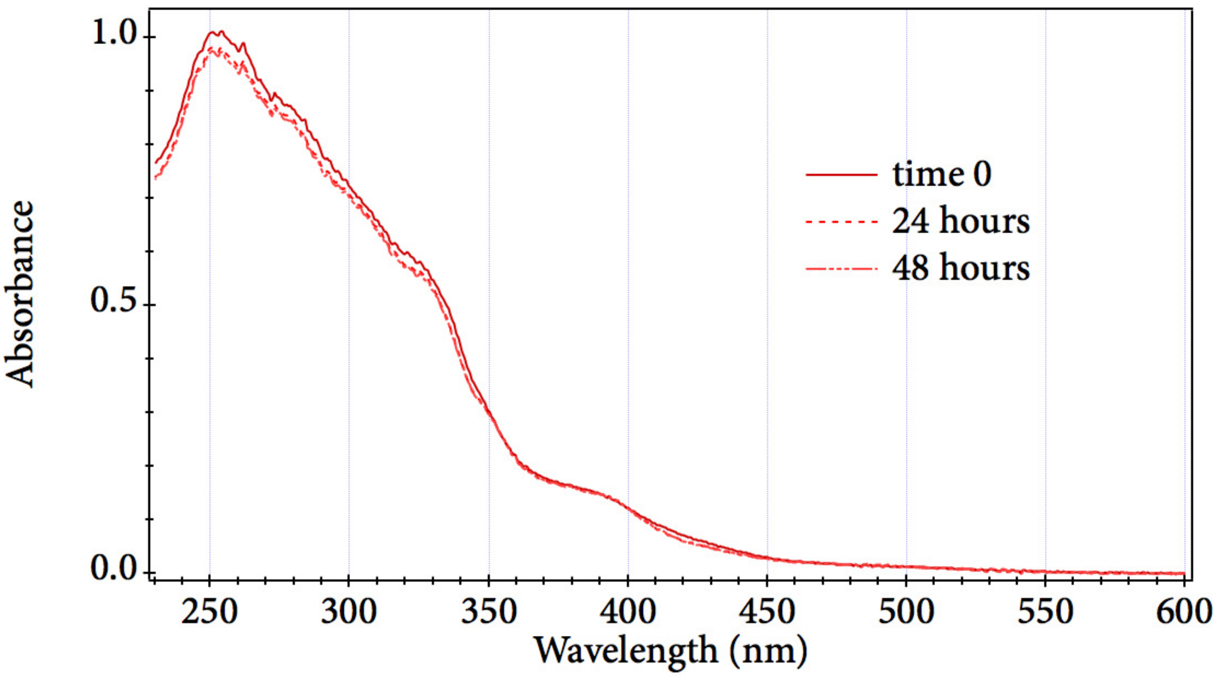

Figure S32. UV-Vis absorption spectra of **2** (3×10^{-5} M) in PB (10×10^{-3} M, pH=7.3). The spectra were recorded at different times, showing no appreciable change after 2 days.

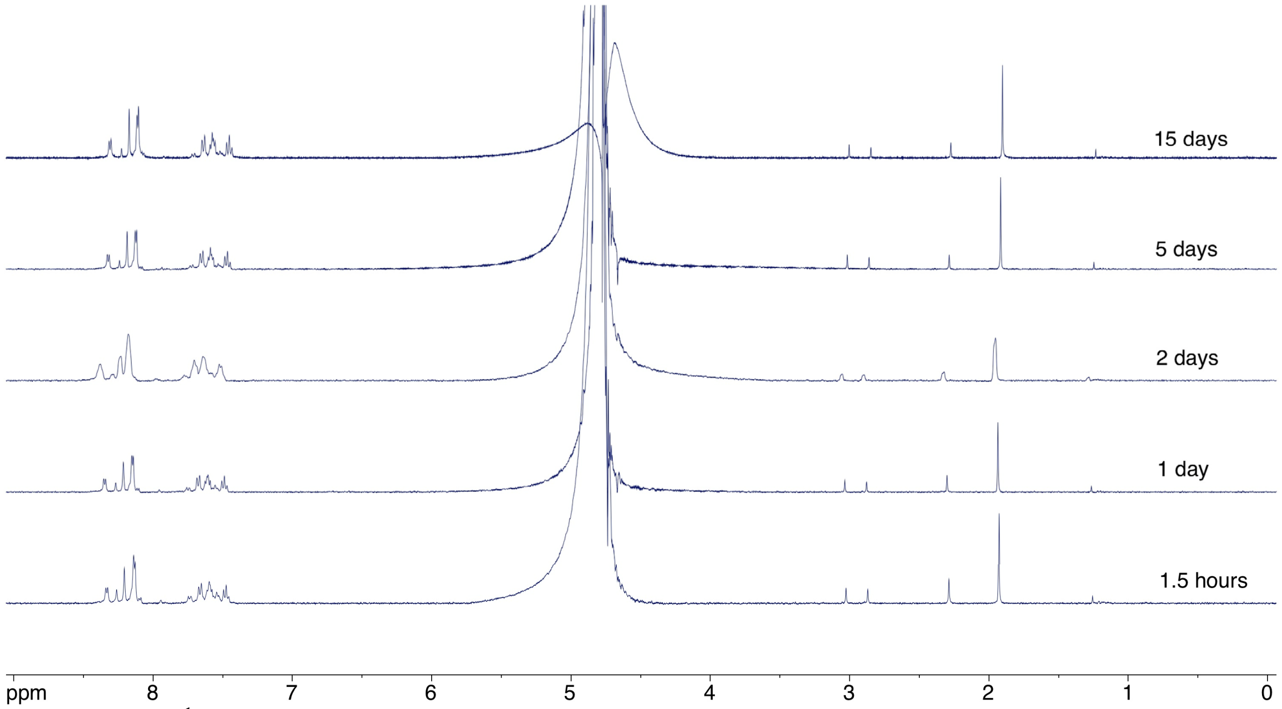

Figure S33. ^{1}H NMR spectra of **2** in D_2O recorded at different times. The spectra are all superimposable, confirming that an initial rapid equilibrium is reached, which does not change over time.

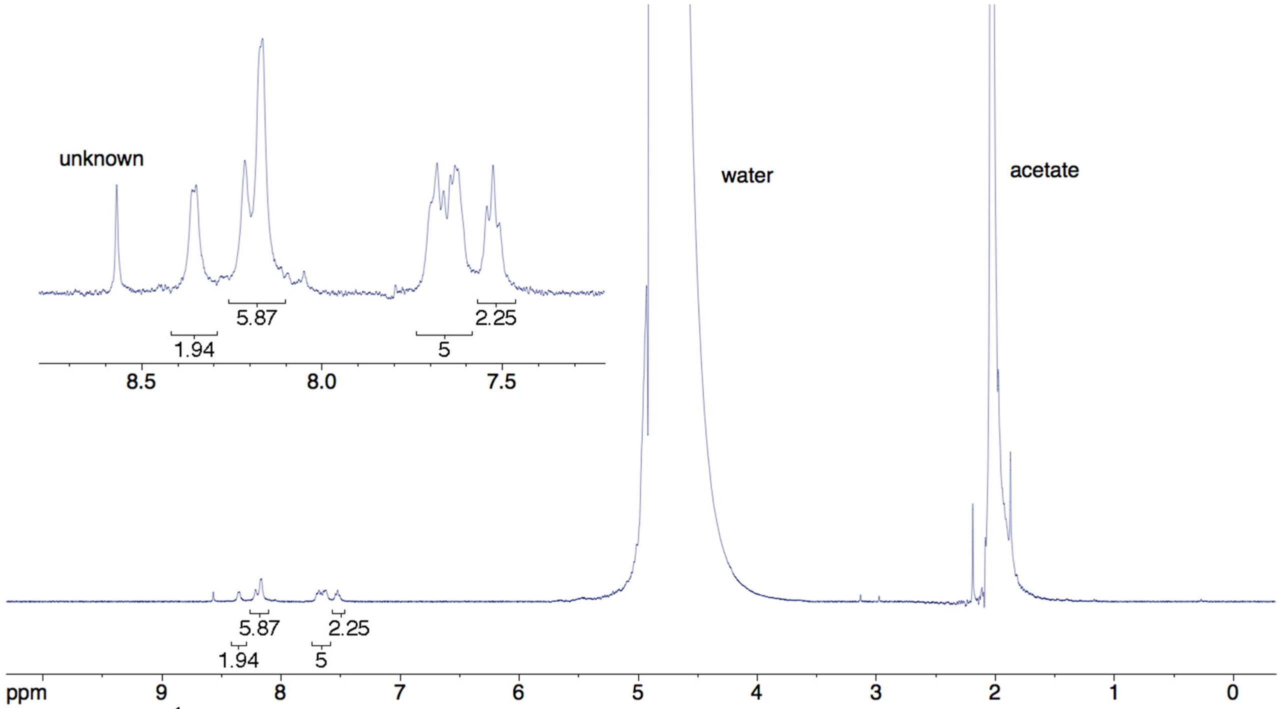

Figure S34. ^{1}H NMR spectrum of **2** with sodium acetate (large excess) in D_2O. The hydrolysis equilibrium of $[Pt(OAc)(phterpy)]^{+}$ is suppressed by the addition of the acetate anion (one platinum species is visible according to the number of the signals and their integration).

7. *Irradiation of the Pt(IV) compound 1.*

After recording the change in the UV-Vis spectra after irradiation, the absorbance at 361 nm and at

344 nm (where the spectral change was maximum) were plotted against the irradiation time. The data points were fitted with an exponential decay, which corresponds to a first order reaction. The data were linearized by plotting $\ln((A_t-A_{inf})/(A_0-A_{inf}))$ versus the irradiation time, which allowed us to obtain the time constant τ.

Irradiation with UV light source (365 nm) followed with UV-Vis spectroscopy

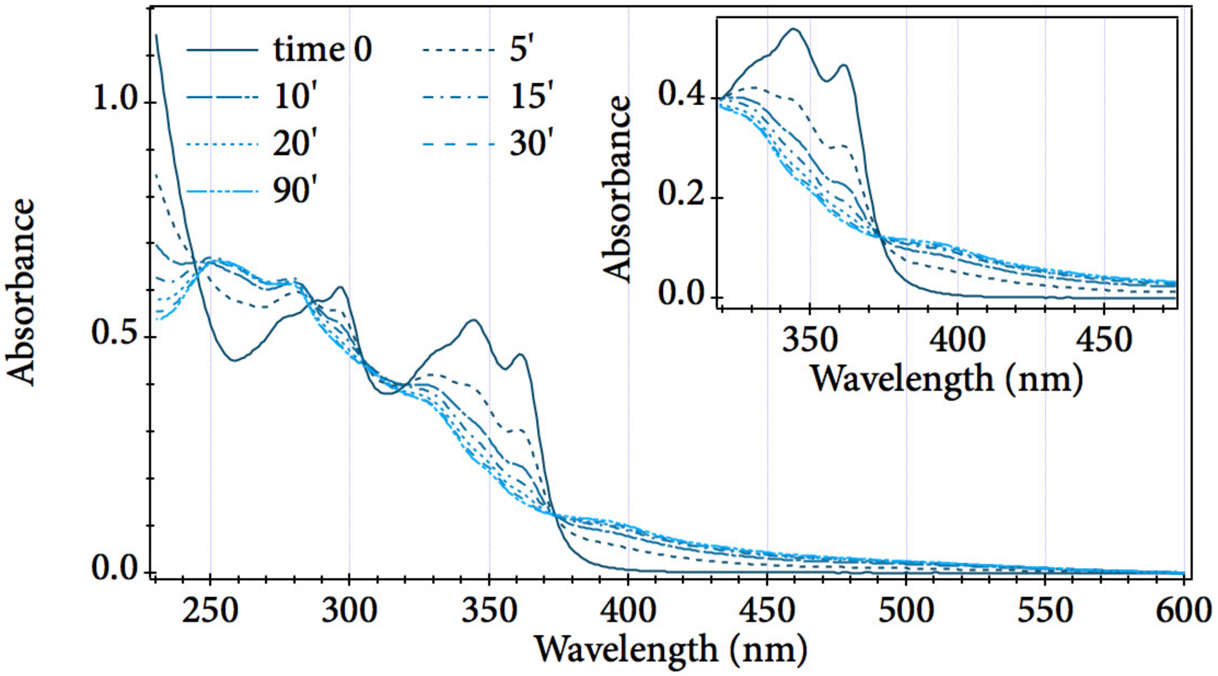

Figure S35. UV-Vis absorption spectra of **1** (3×10^{-5} M) in PB (10×10^{-3} M, pH=7.3). The spectra were recorded after irradiation with a UV lamp operating at 365 nm at the time specified in the legend. The inset shows the neat isosbestic point at 374 nm.

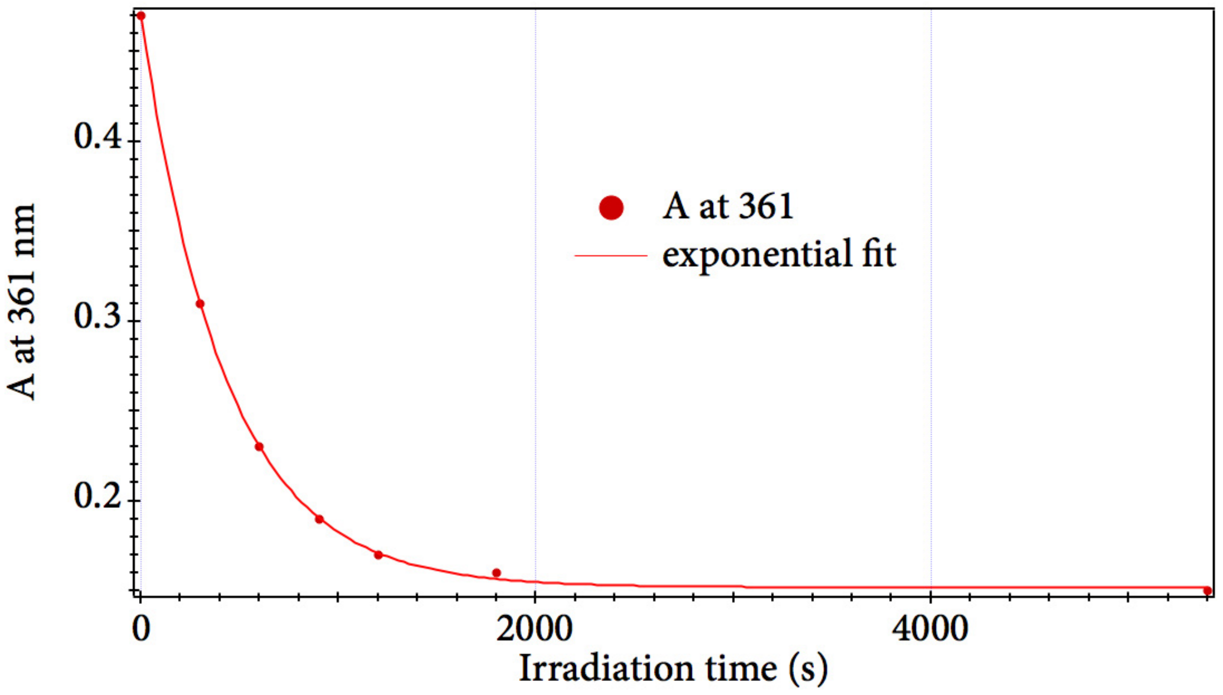

Figure S36a. Absorbance at the maximum (361 nm) from Figure S35 plotted against the irradiation time. **1** decays exponentially with time, reaching a plateau after less than 30 minutes in our conditions (power of the lamp = 5.5mW×cm^{-2}, irradiation time and concentrations specified in Figure S35).

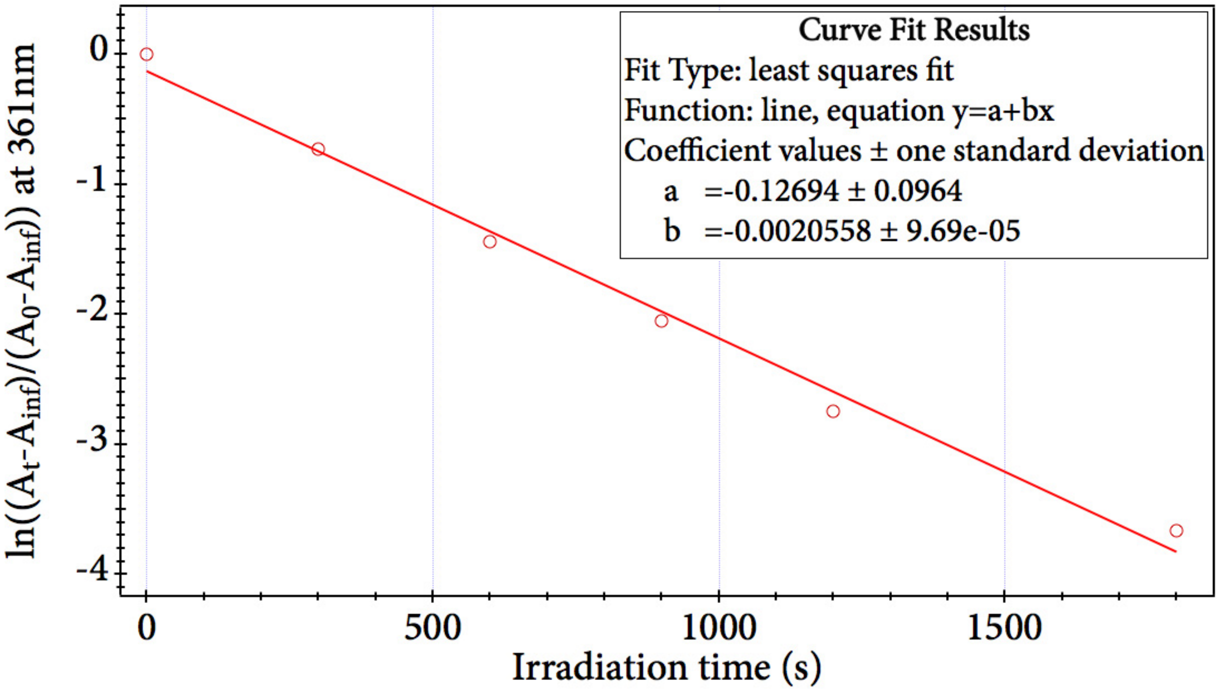

Figure S36b. Plot of ln((A_t-A_{inf})/(A_0-A_{inf}) against irradiation time at 361 nm for compound **1**. The linearization of the data is done based on the graph in Figure S36a and provides the value of τ=486s (obtained as τ=-1/b).

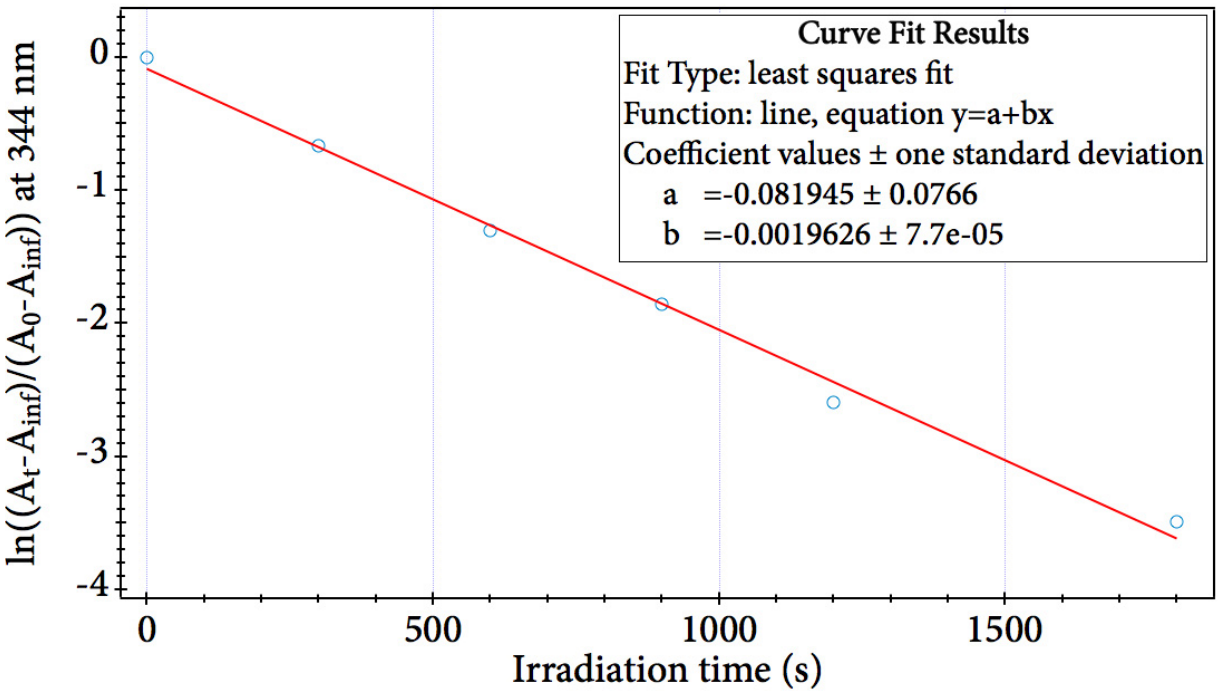

Figure S36c. Plot of $\ln((A_t-A_{inf})/(A_0-A_{inf})$ against irradiation time at 344 nm for compound **1**. The linearization of the data is done based on the graph in Figure S36a and provides the value of τ=510s (obtained as τ=-1/b).

The mean τ value reported from Figures S36b and S36c is 498 s.

Irradiation with Xenon lamp (longpass filter, λ>400 nm), followed with UV-Vis spectroscopy

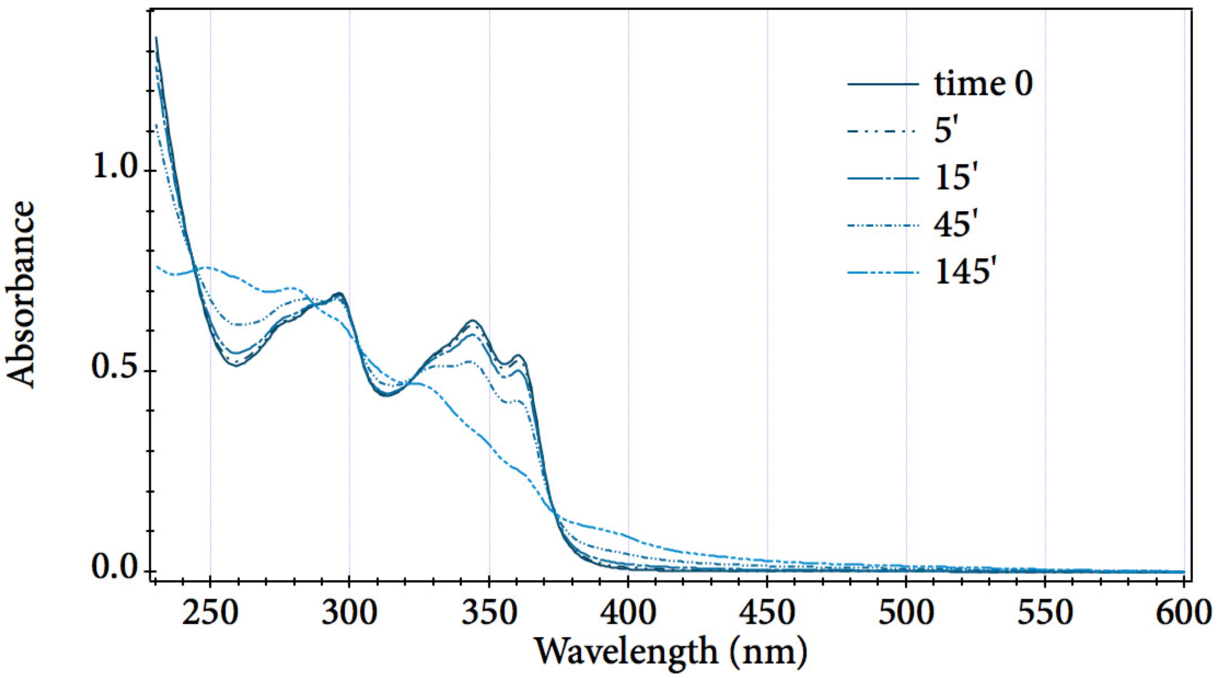

Figure S37. UV-Vis absorption spectra of **1** (3×10^{-5} M) in PB (10×10^{-3} M, pH=7.3) irradiated with a Xenon lamp, equipped with a longpass filter that cut all the wavelengths shorter than 400 nm. The irradiation time is specified in the legend.

Xenon lamp without the longpass filter.

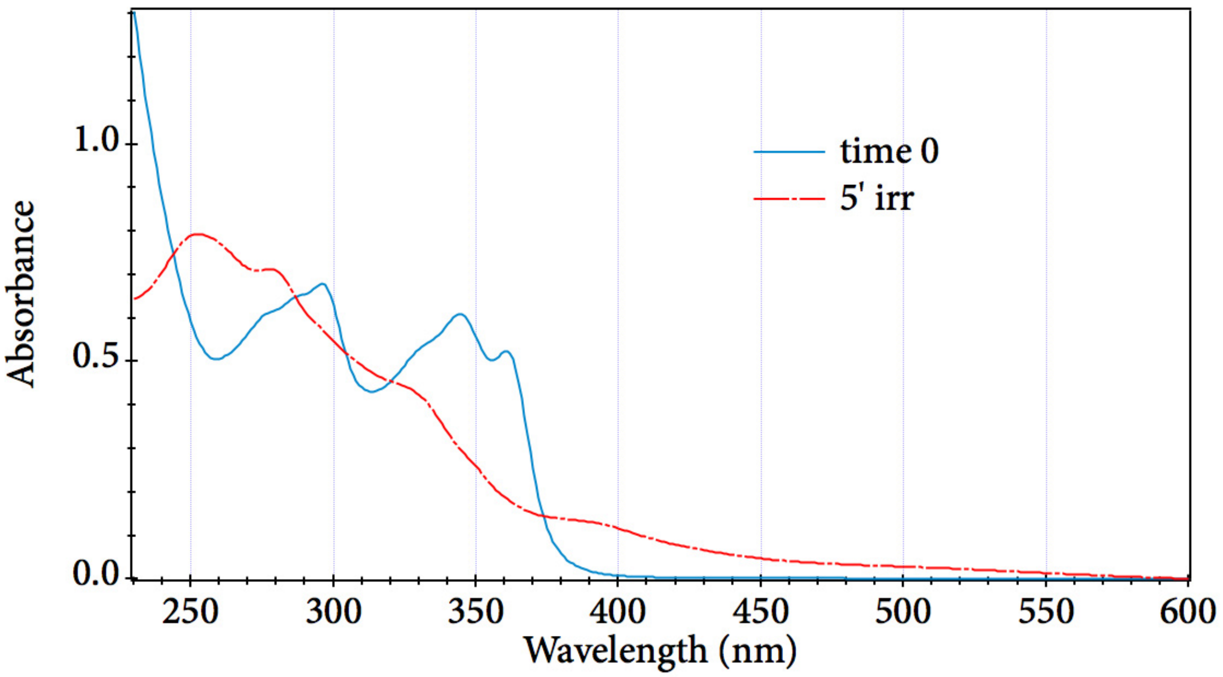

Figure S38. UV-Vis absorption spectra of **1** (3×10^{-5} M) in PB (10 mM, pH=7.3). The spectra were recorded before and after 5 min of irradiation.

Irradiation with UV light source (365 nm) followed with ^{1}H-NMR spectroscopy

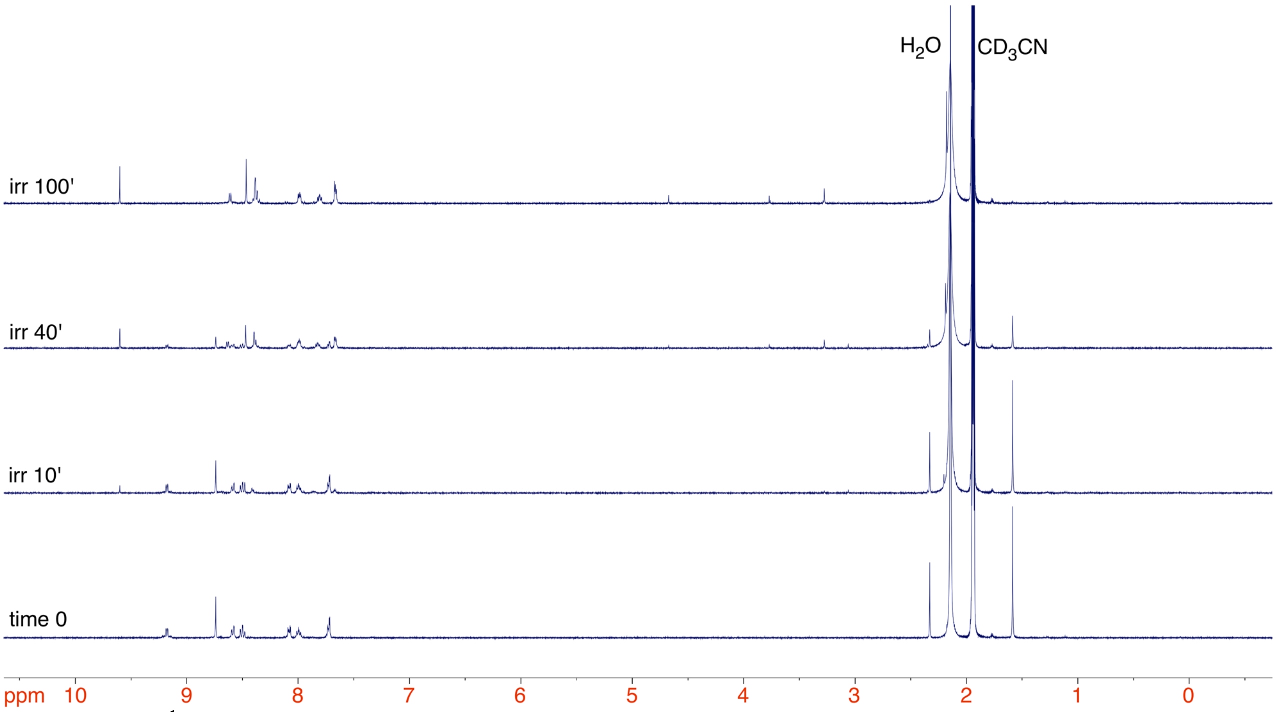

Figure S39. ^{1}H NMR spectra obtained after irradiation of complex **1**. The total irradiation time was 100 minutes, during which we observed the complete conversion from **1** to **2**. The total light dose delivered was 33 J×cm^{-2} greater than in the spectrophotometric experiment (Figure S35) due to the different concentration of the species in solution. The signal around 9.5 ppm and those between 3 and 5 ppm could be due to the degradation of the solvent CH_3CN for the prolonged irradiation time.

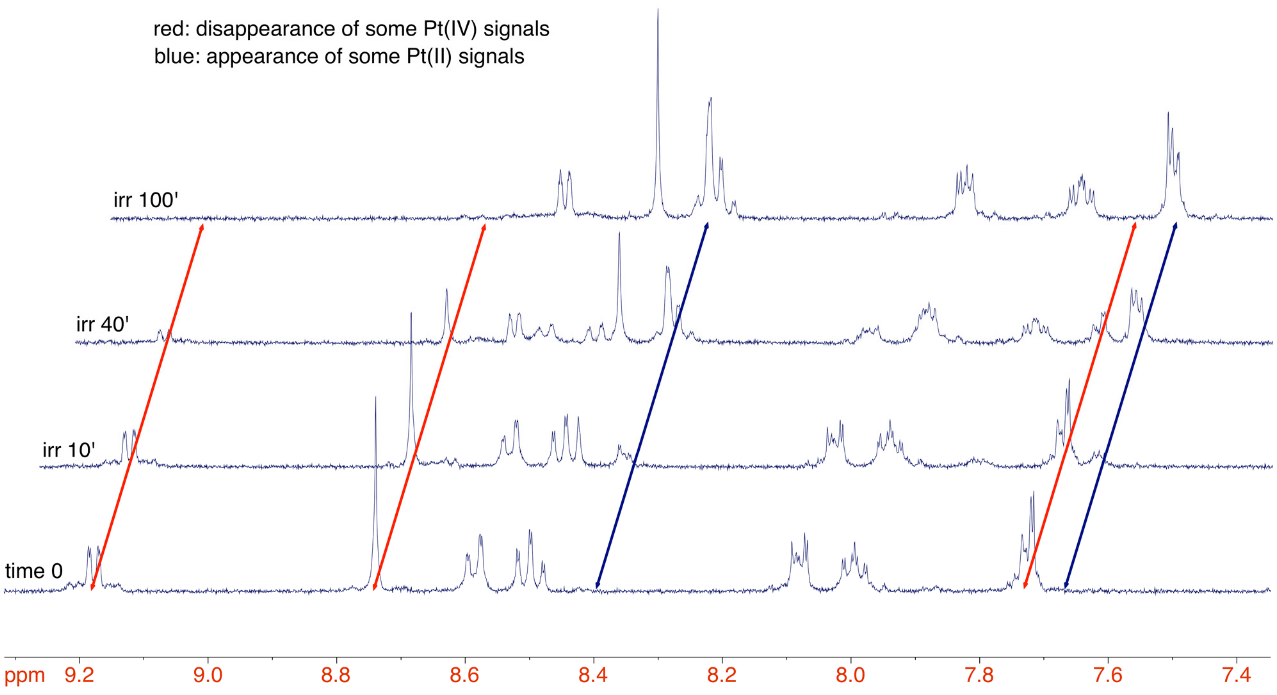

Figure S40. ^{1}H NMR spectra obtained after irradiation of complex **1**, aromatic portion enlarged. Compound **1**, present at time 0, is fully converted to **2**, the only one present at 100’ of irradiation.

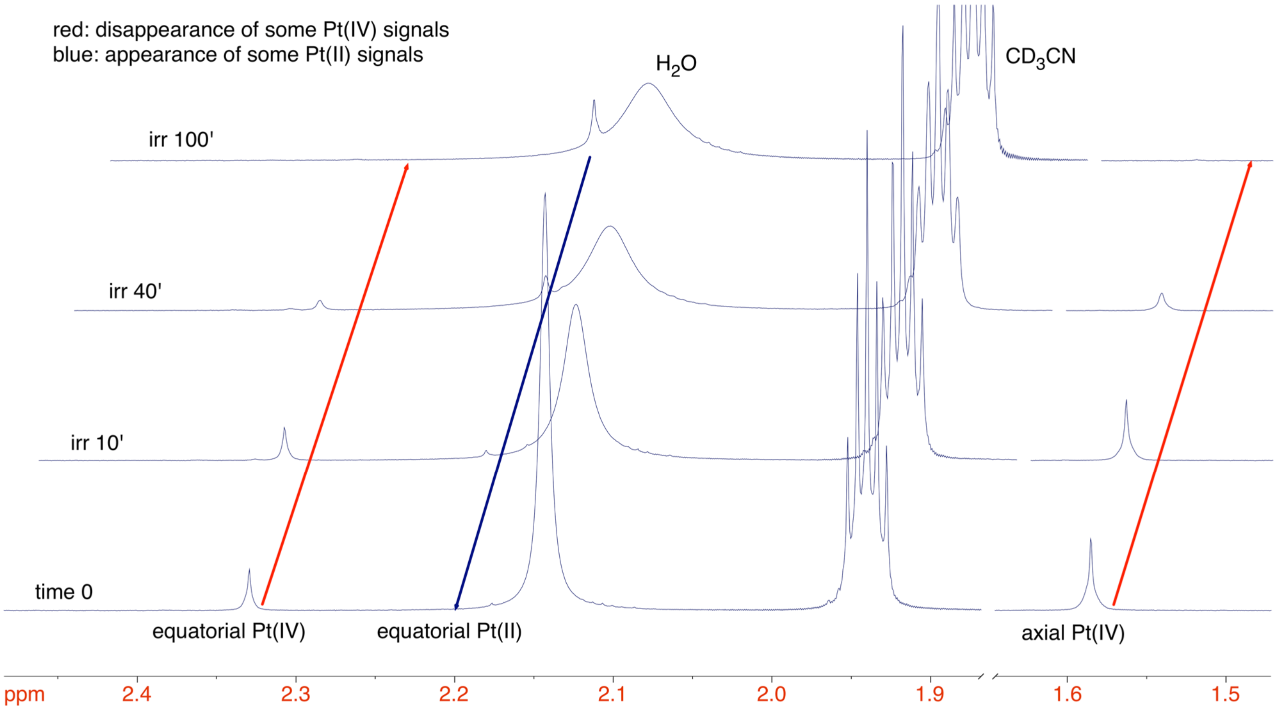

Figure S41. ^{1}H NMR spectra obtained after irradiation of complex **1**, aliphatic portion enlarged. Compound **1**, present at time 0, is fully converted to **2**.

8. ***Photocatalysis studies***

Irradiation with the LED lamp at 460 nm

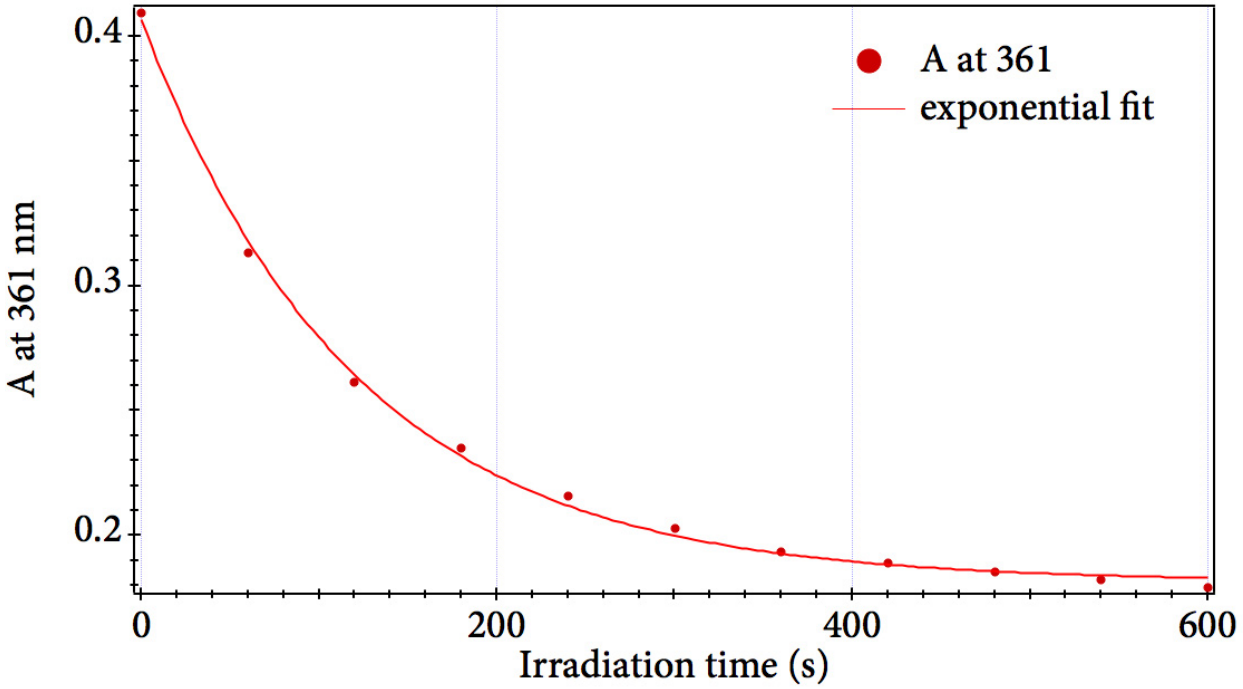

Figure S42a. Absorbance at maximum (361 nm) plotted against the irradiation time, data taken from Figure **2** in main text, where photoconversion of **1** (20×10^{-6} M) with NADH (40×10^{-6} M) and FMN (1×10^{-6} M) in PB (50×10^{-3} M, pH=7.0) was depicted. As shown in the legend, **1** decays exponentially with time.

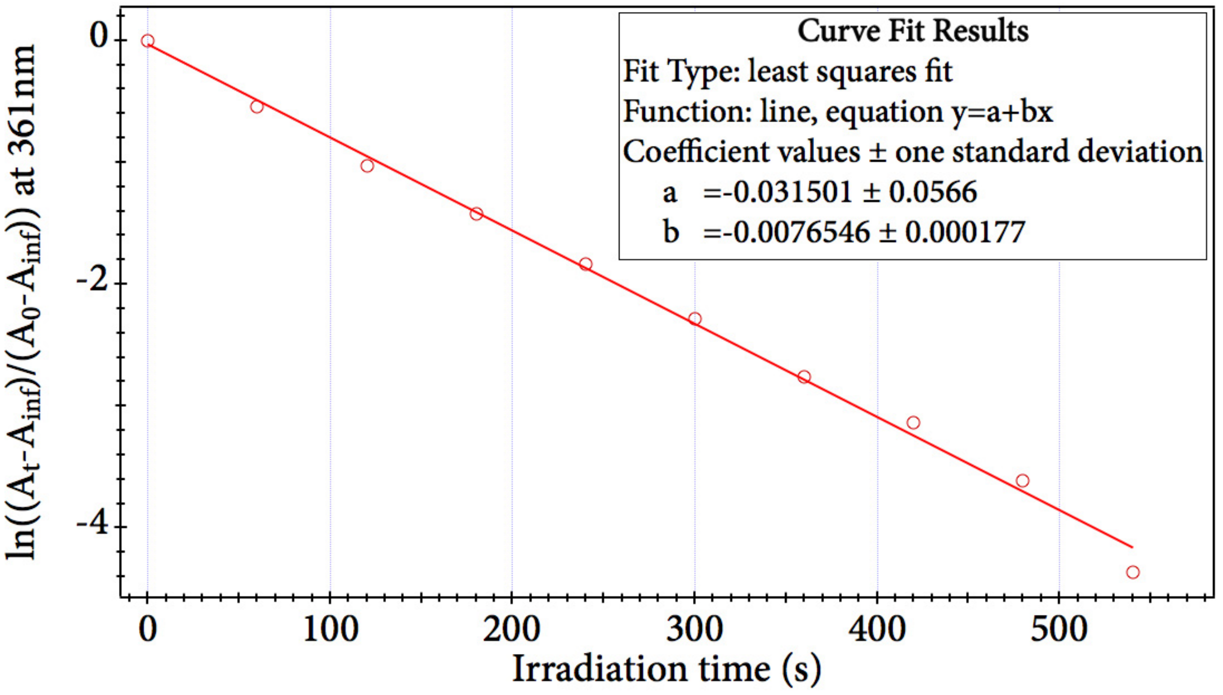

Figure S42b. Plot of ln((A_t-A_{inf})/(A_0-A_{inf})) against irradiation time at 361 nm for compound **1**. The linearization of the data is done based on the graph in Figure S42b and provides the value of τ=131s (obtained as τ=-1/b).

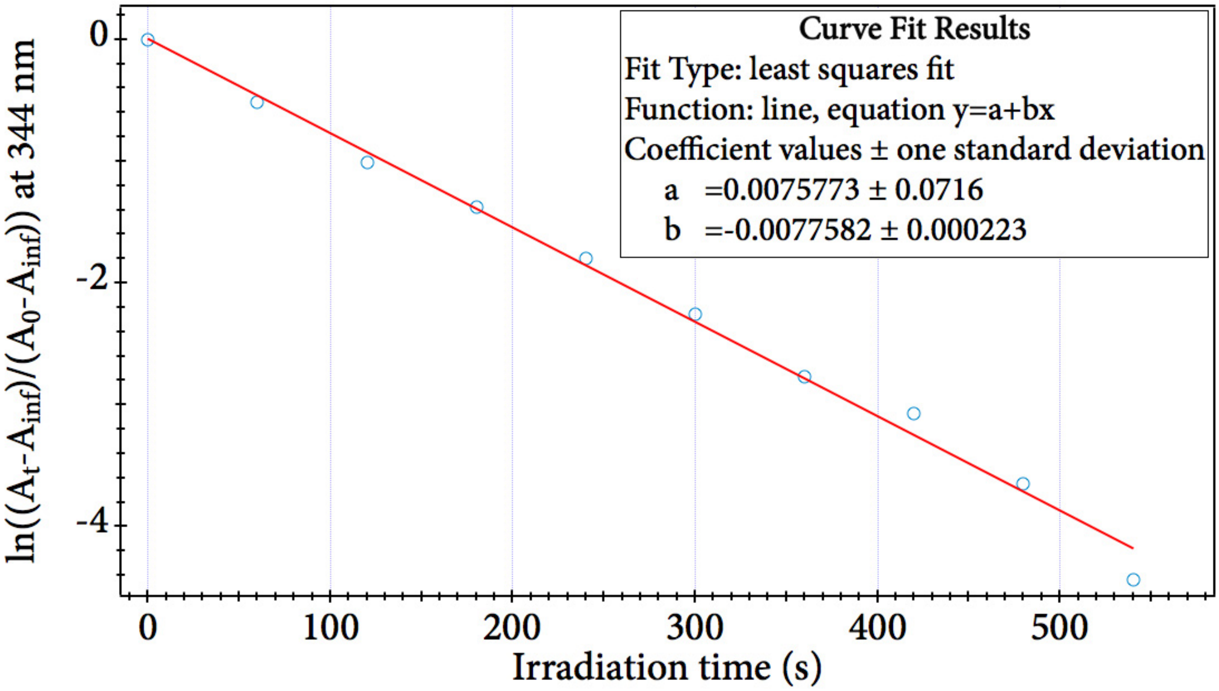

Figure S42c. Plot of ln((A_t-A_{inf})/(A_0-A_{inf})) against irradiation time at 344 nm for compound **1**. The linearization of the data is done based on the graph in Figure S42b and provides the value of τ=129s (obtained as τ=-1/b).

The mean τ value obtained from Figures S42b and S42c is 130s.

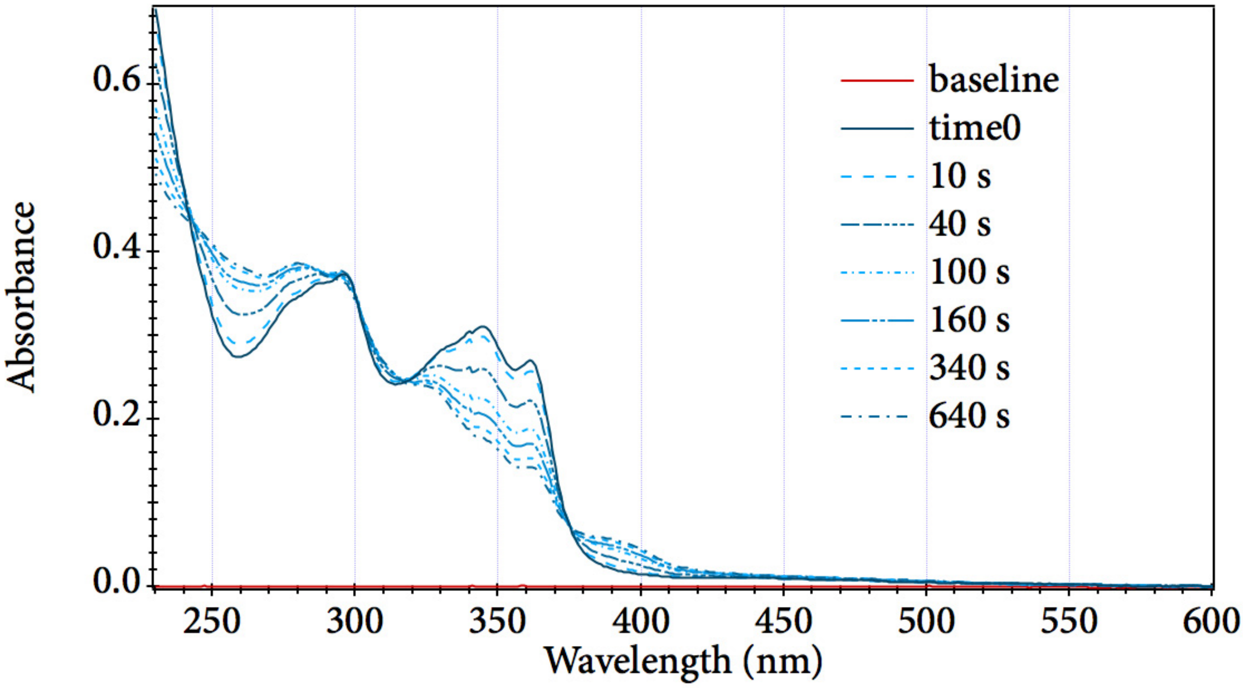

Figure S43. Photoconversion of **1** (15×10^{-6} M) with NADH (30×10^{-6} M) and FMN (1×10^{-6} M) in PB (50×10^{-3} M, pH=7.0).

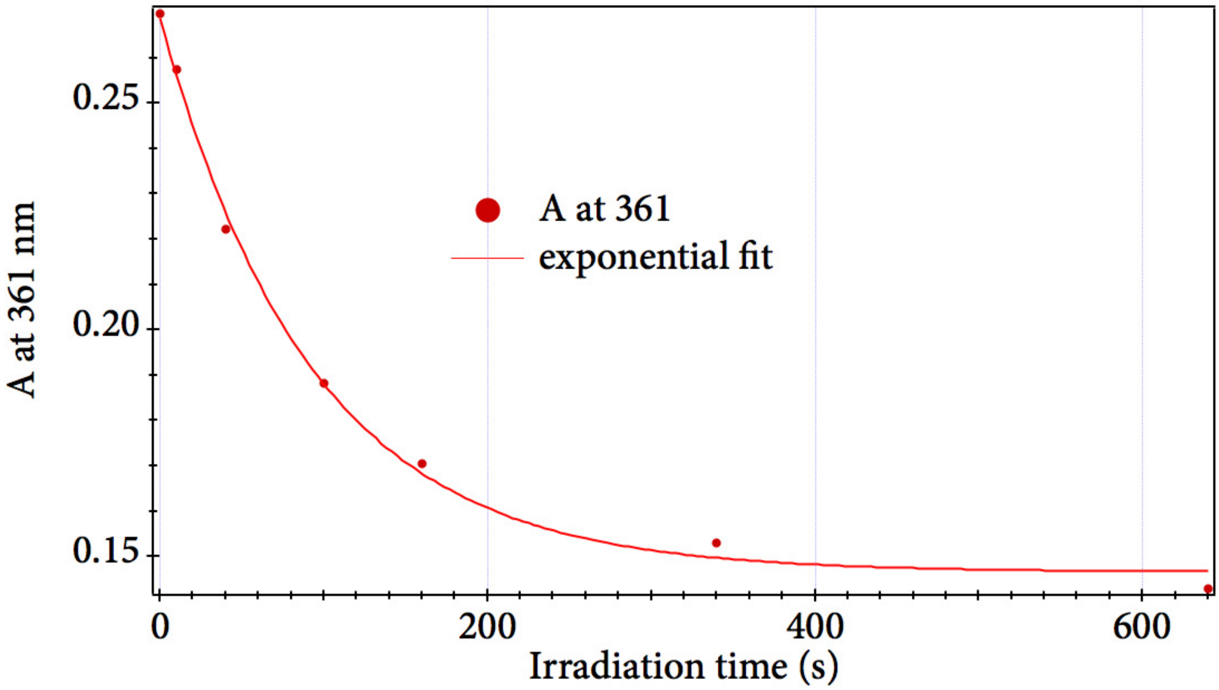

Figure S44a. Absorbance at the maximum (361 nm) from Figure S43 plotted against the irradiation time. **1** decays exponentially with time.

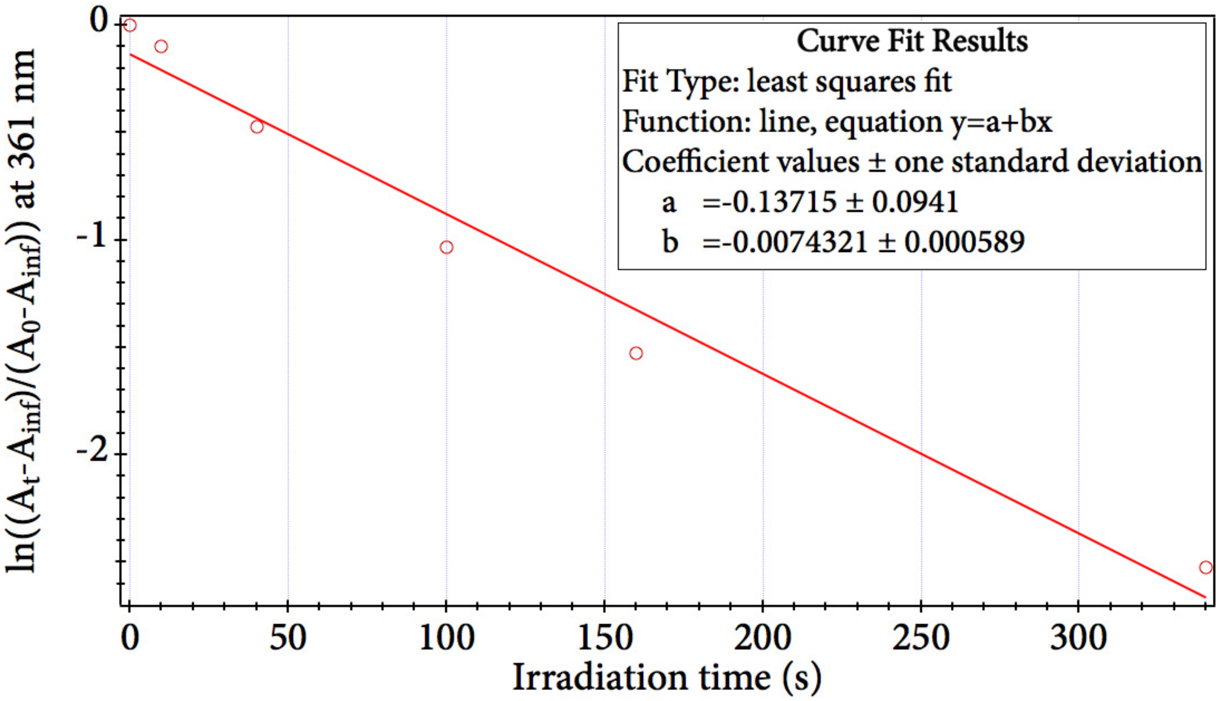

Figure S44b. Plot of $\ln((A_t-A_{inf})/(A_0-A_{inf})$ against irradiation time at 361 nm for compound **1**. The linearization of the data is done based on the graph in Figure S44a and provides the value of τ=134s (obtained as τ=-1/b).

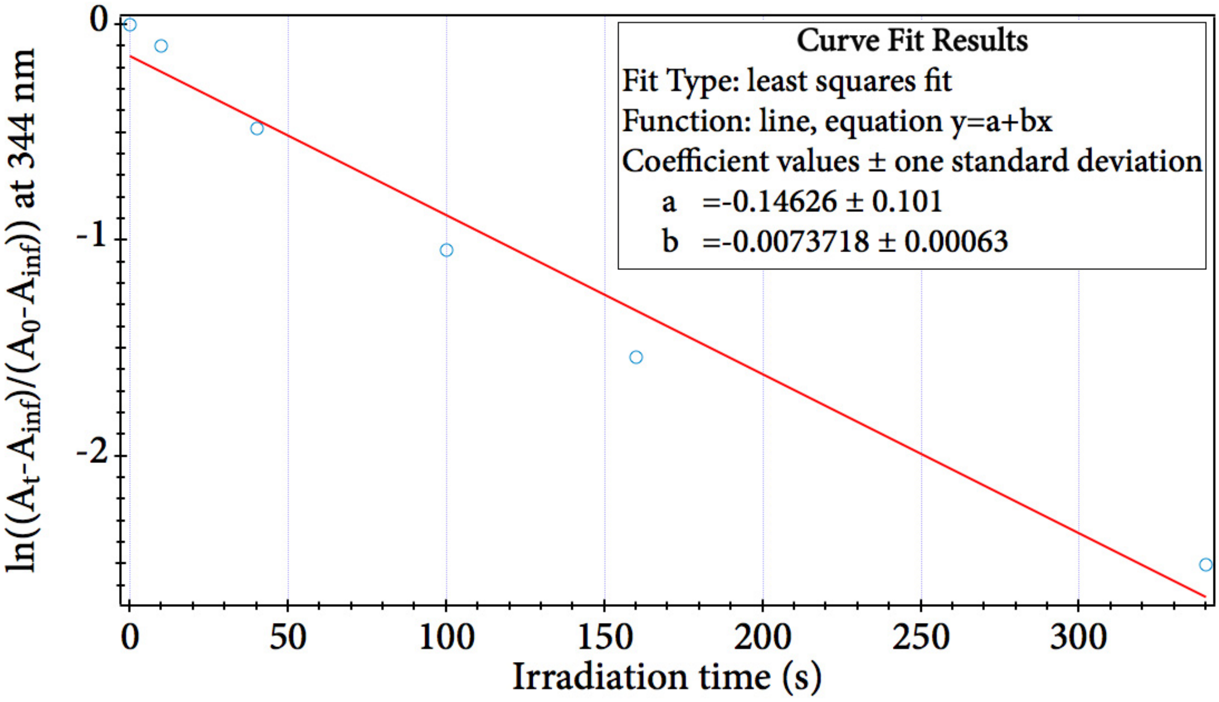

Figure S44c. Plot of ln((A_t-A_{inf})/(A_0-A_{inf}) against irradiation time at 344 nm for compound **1**. The linearization of the data is done based on the graph in Figure S44a and provides the value of τ=136s (obtained as τ=-1/b).

The mean τ value obtained from Figures S44b and S44c is 135s.

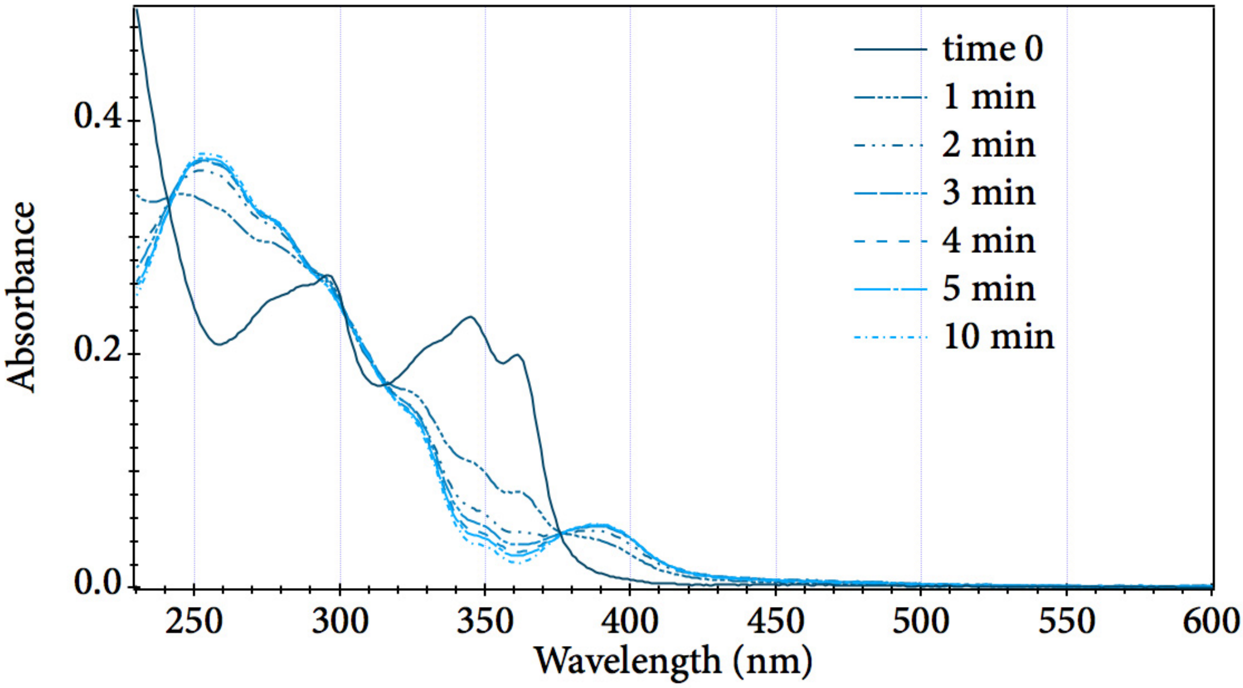

Figure S45. Irradiation of **1** (10×10^{-6} M) with NADH (100×10^{-6} M) and FMN (1×10^{-6} M) in PB (50×10^{-3} M, pH=7.0).

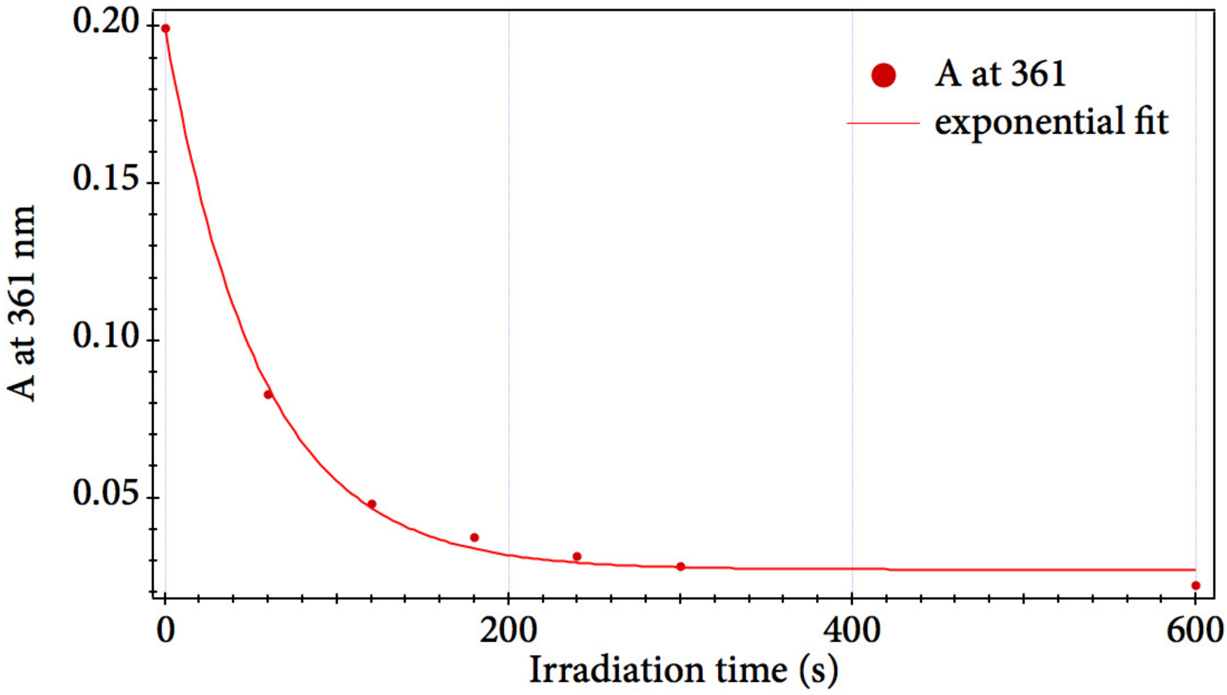

Figure S46a. Absorbance at the maximum (361 nm) from Figure S45 plotted against the irradiation time. **1** decays exponentially with time.

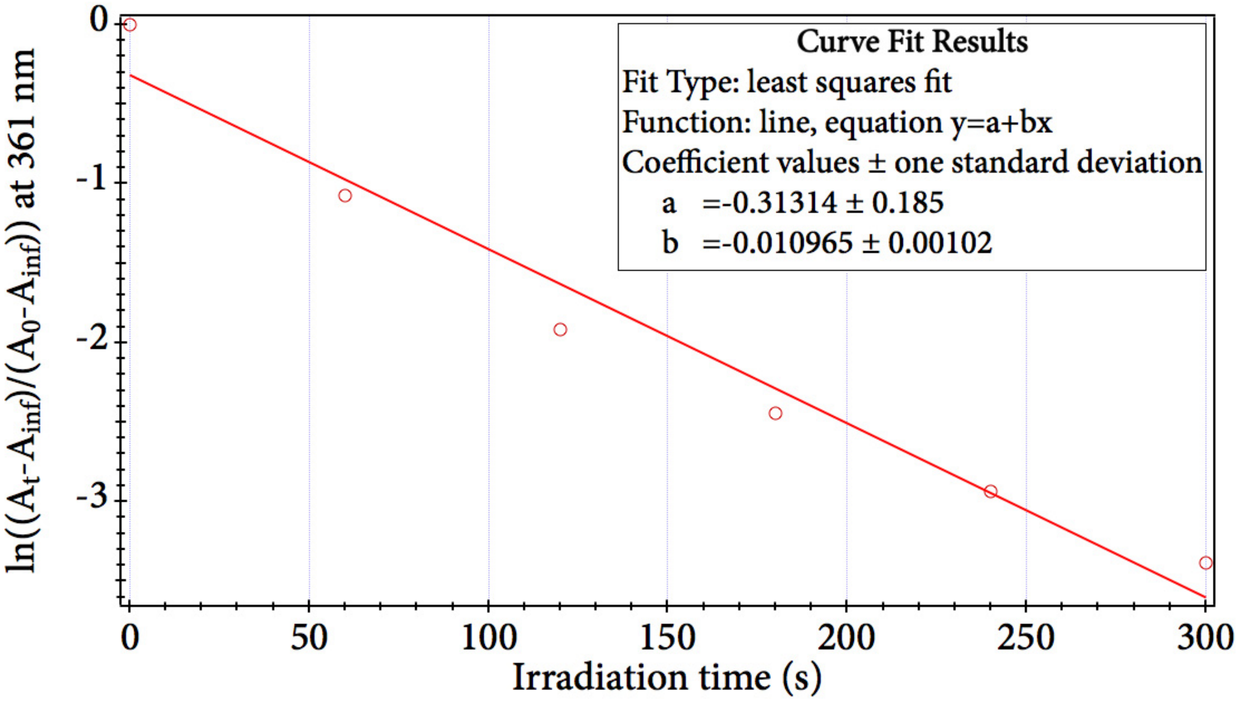

Figure S46b. Plot of $\ln((A_t - A_{inf})/(A_0 - A_{inf})$ against irradiation time at 361 nm for compound **1**. The linearization of the data is done based on the graph in Figure S46a and provides the value of τ=91s (obtained as τ=-1/b).

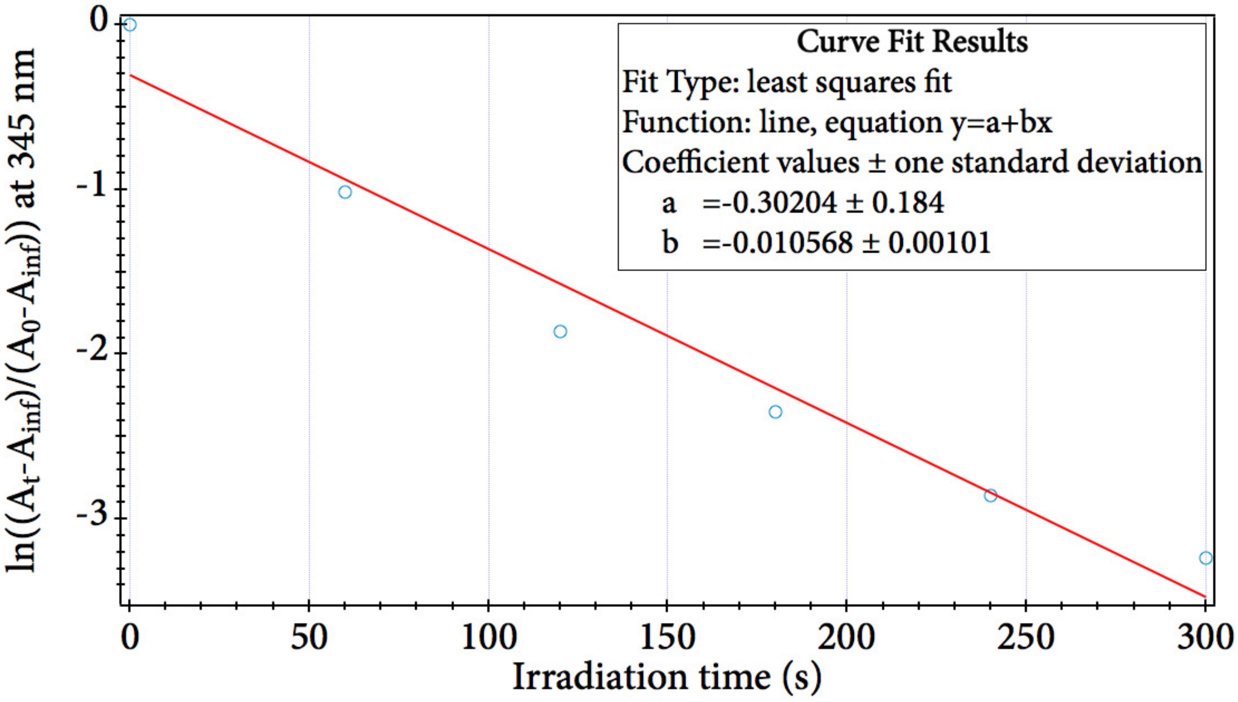

Figure S46c. Plot of $\ln((A_t\text{-}A_{inf})/(A_0\text{-}A_{inf})$ against irradiation time at 345 nm for compound **1**. The linearization of the data is done based on the graph in Figure S46b and provides the value of τ=95s (obtained as τ=-1/b).

The mean τ value obtained from Figures S46b and S46c is 93s.

Stability studies

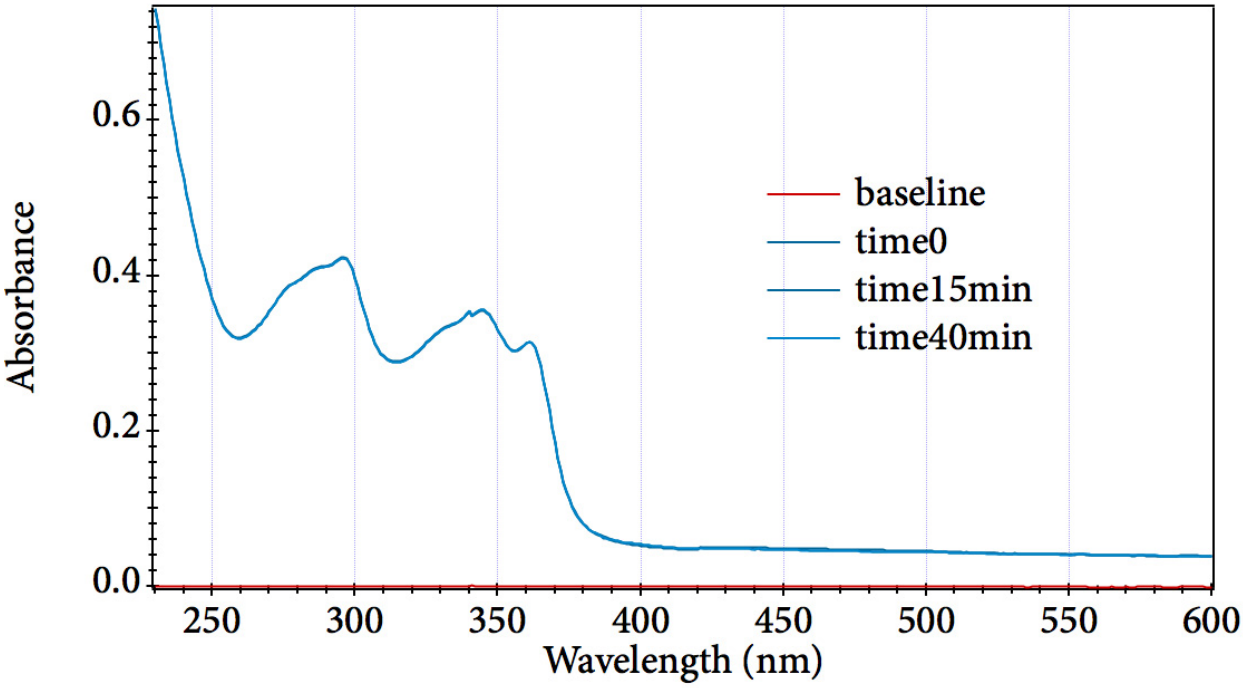

Figure S47. UV-Vis absorption spectra of **1** (15×10^{-6} M) in the presence of NADH (30×10^{-6} M) in PB (50×10^{-3} M, pH=7.0), recorded at subsequent times to see if degradation takes place.

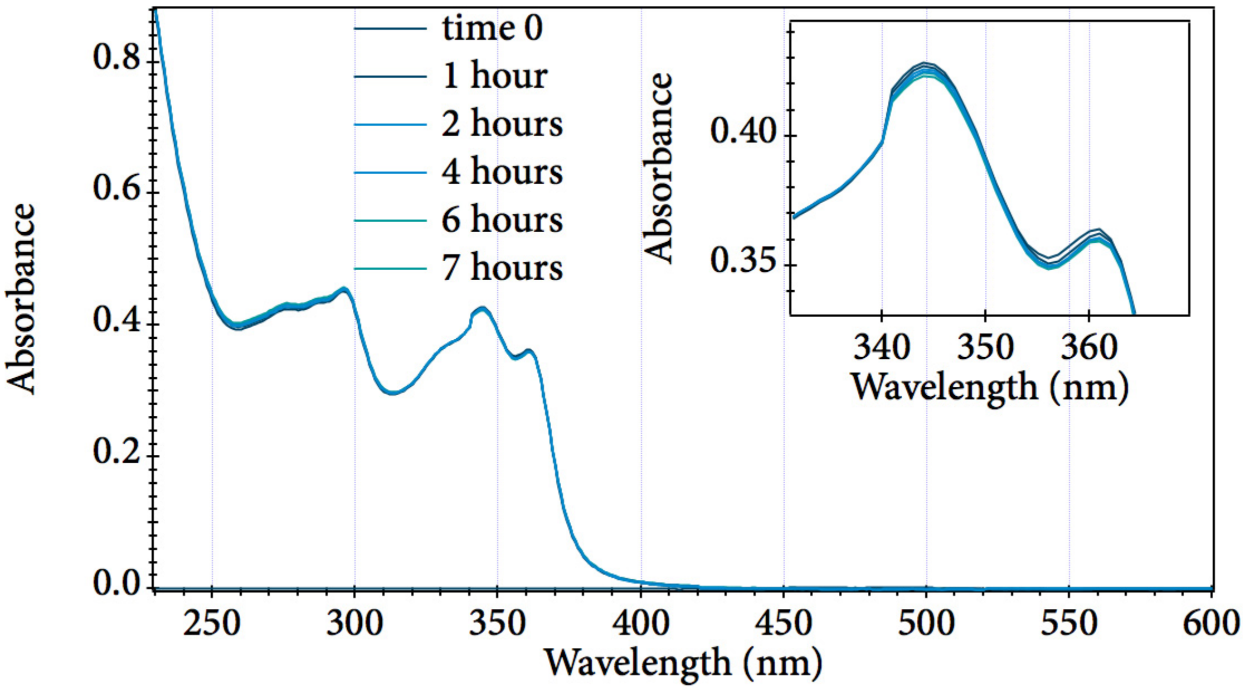

Figure S48. UV-Vis absorption spectra of **1** (20×10^{-6} M) in the presence of NADH (40×10^{-6} M) in PB (50×10^{-3} M, pH=7.0), recorded at subsequent times in the dark, to see if degradation takes place.

The dark stability experiment was repeated with a large excess of NADH (1:10 equivalents), firstly in the dark and with irradiation. The dark stability showed almost no change for 6 hours, conc: [**1**] = 10 uM; [NADH]=100 uM. Then we irradiated the system for 10 minutes and we saw a very small reduction to Pt(II), because of the decrease of the band at 361 nm. Anyway, this reduction is much smaller with respect to what we observe in the presence of FMN for the same irradiation time.

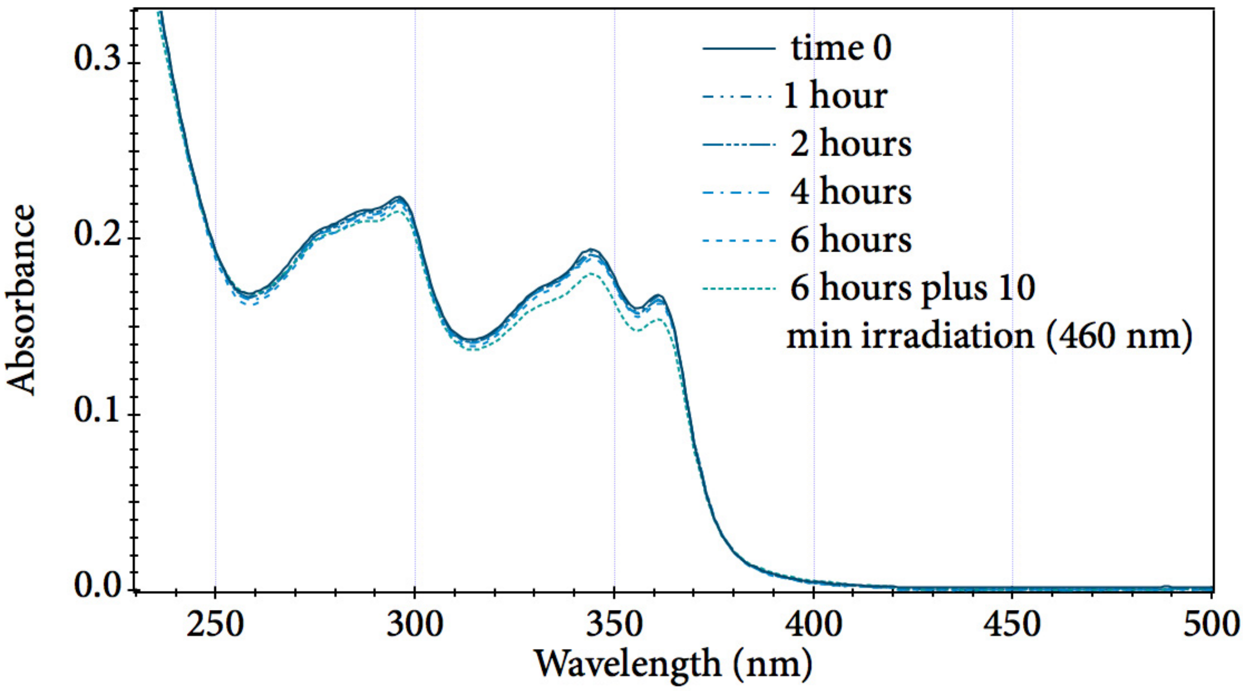

Figure S49. UV-Vis absorption spectra of **1** (10×10^{-6} M) in the presence of NADH (100×10^{-6} M) in PB (50×10^{-3} M, pH=7.0), recorded at subsequent times in the dark and with 10 minutes of irradiation.

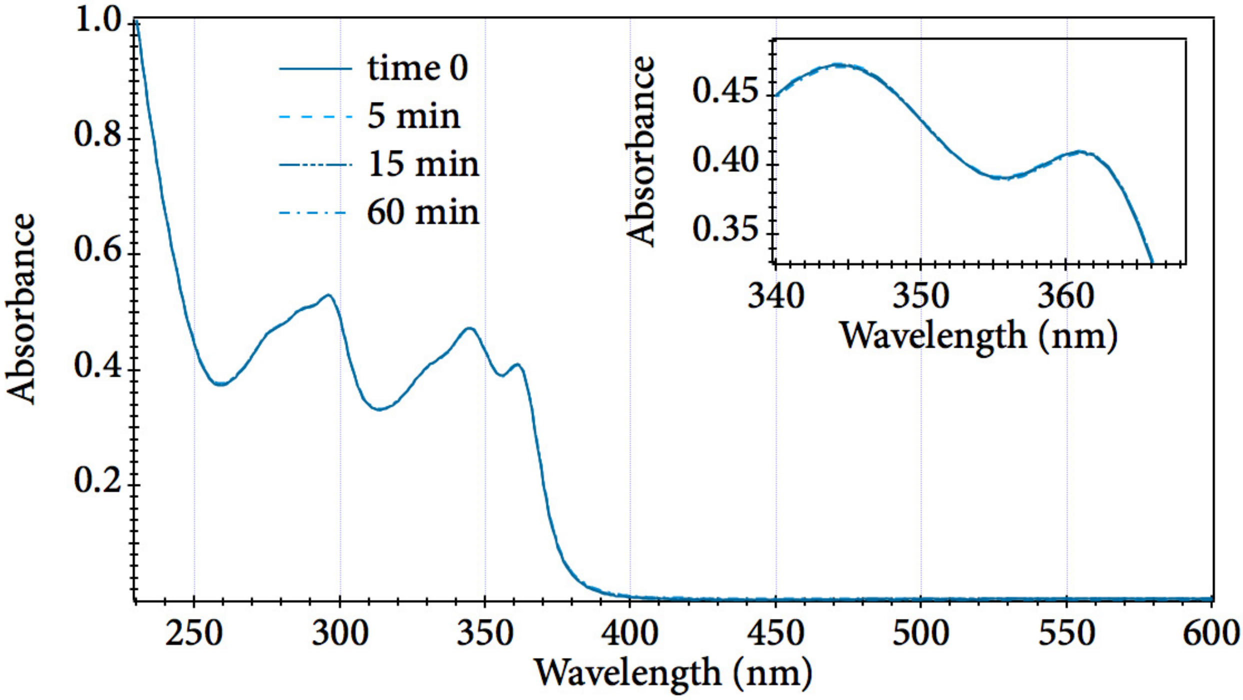

Figure S50. UV-Vis absorption spectra of **1** (20×10^{-6} M) in the presence of NADH (40×10^{-6} M) and FMN (1×10^{-6} M) in PB (50×10^{-3} M, pH=7.0), recorded at subsequent times in the dark, to see if degradation takes place. No interaction was visible during this time scale.

^{1}H NMR experiments

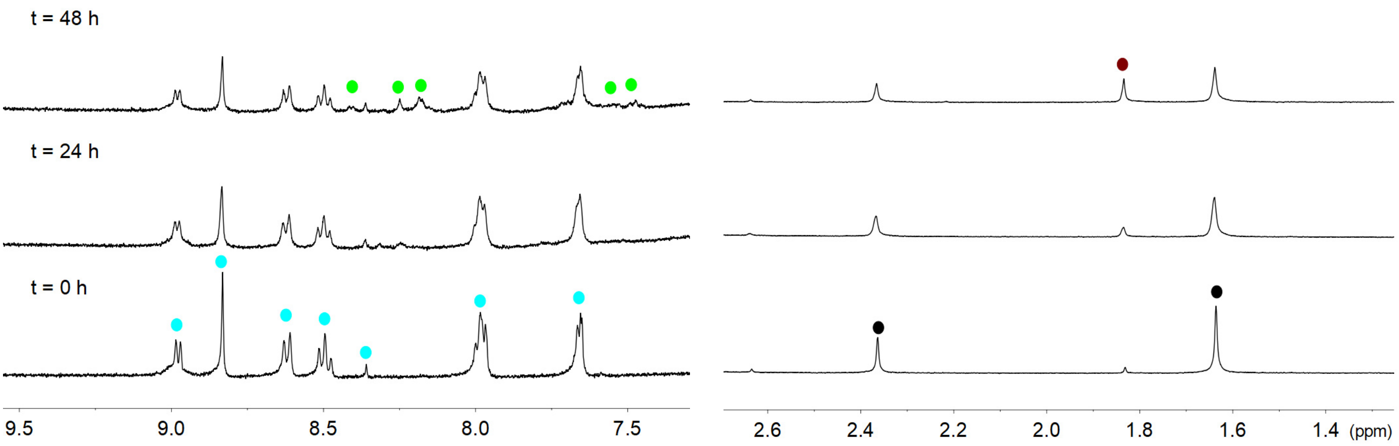

Figure S51. Dark stability of complex **1** (500 µM) in 18mM MES (pH 6.0, 10%D_2O) for 48 h. ^{1}H NMR signal labelling: ● Complex **1** Pt (IV), ● Complex **2** Pt (II), ● Pt–OCO**CH₃** Pt (IV), ● free $^-$OCO**CH₃**.

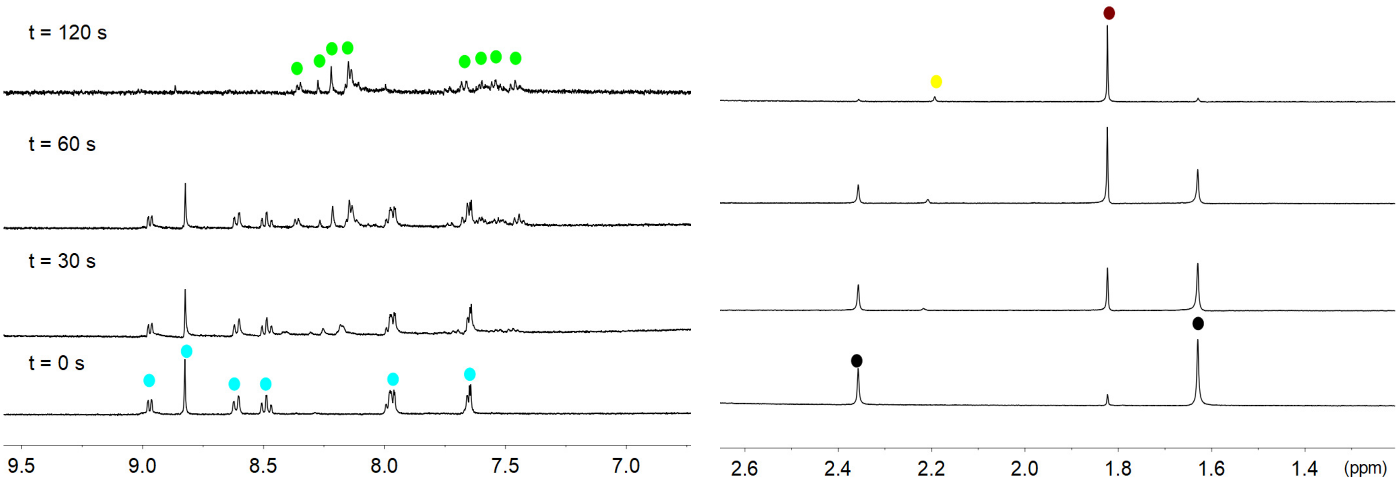

Figure S52. Photocatalytic activation of complex **1** (500 µM) in 18mM MES (pH 6.0, 10%D_2O) in the presence of 1 µM **FMN,** under 460-nm light irradiation (6 mW×cm^{-2}).

^{1}H NMR signal labelling: ● Complex **1** Pt (IV), ● Complex **2** Pt (II), ● Pt–OCO**CH₃** Pt (IV), ● free $^-$OCO**CH₃**, ● Pt–OCO**CH₃** Pt (II).

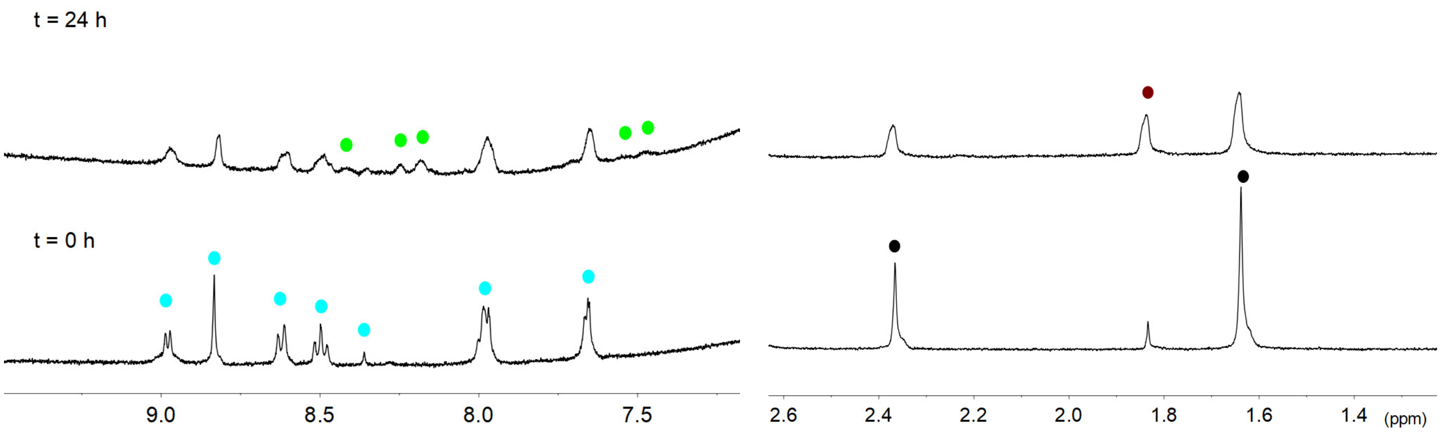

Figure S53. Dark stability of complex **1** (500 μM) in 18mM MES (pH 6.0, 10%D_2O) in the presence of 1 μM **FMN** for 24 h.

^{1}H NMR signal labelling: ● Complex **1** Pt (IV), ● Complex **2** Pt (II), ● Pt–OCO**CH$_3$** Pt (IV), ● free $^-$OCO**CH$_3$**.

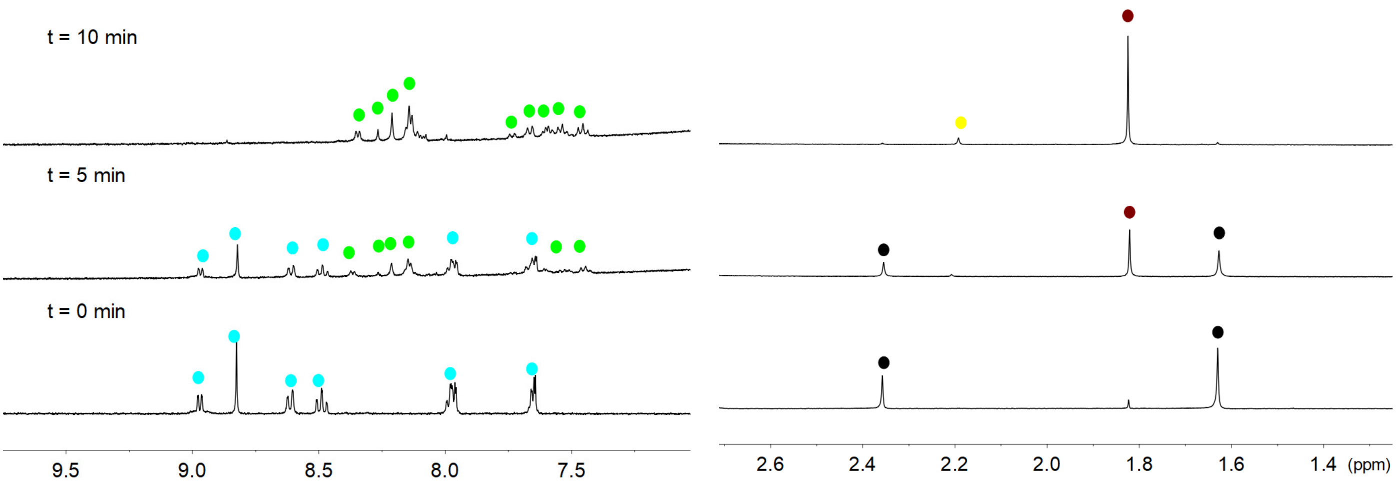

Figure S54. Photostability of complex **1** (500 μM) in 18mM MES (pH 6.0, 10%D_2O) for 10 minutes under 460-nm light irradiation (6 $mW \times cm^{-2}$).

^{1}H NMR signal labelling: ● Complex **1** Pt (IV), ● Complex **2** Pt (II), ● Pt–OCO**CH$_3$** Pt (IV), ● free $^-$OCO**CH$_3$**, ● Pt–OCO**CH$_3$** Pt (II).

9. ***Electrochemistry.***

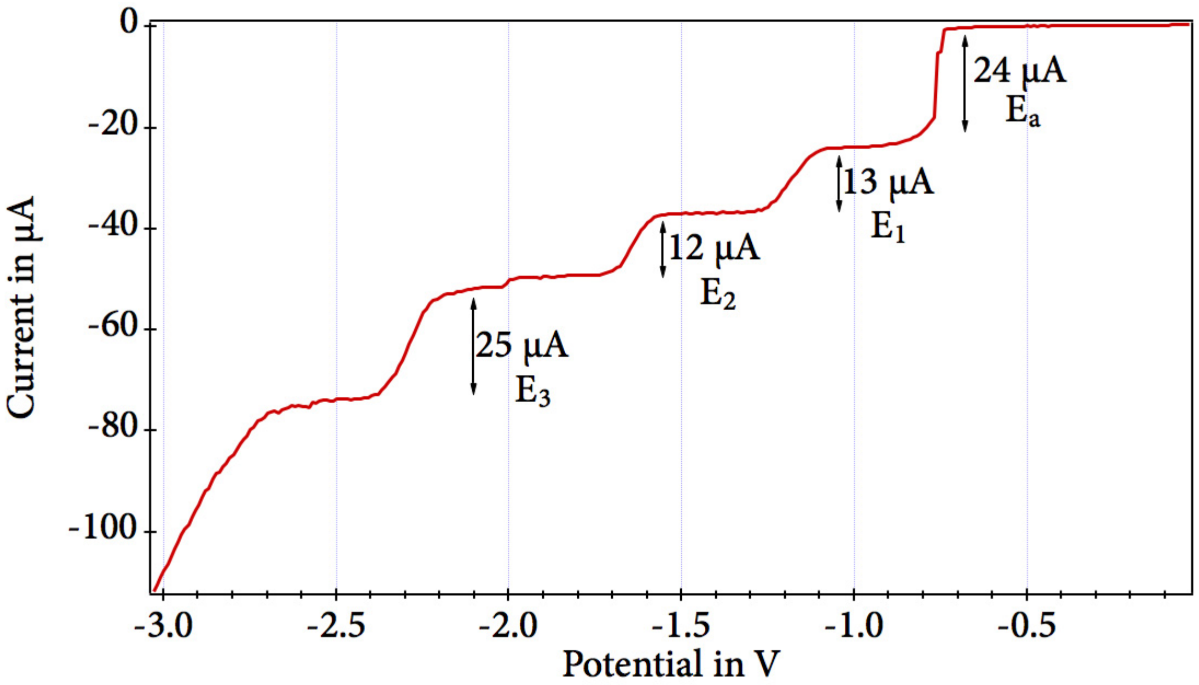

Figure S55. Hydrodynamic voltammetry of **1** (10^{-3} M) in DMSO with $[N^nBu_4][CF_3SO_3]$ (0.1 M) as supporting electrolyte. The limiting current for E_a is the double with respect to E_1 and E_2. E_3 seems to be a two-electron process, but the CV reveals that two one electron waves are very close to each other.

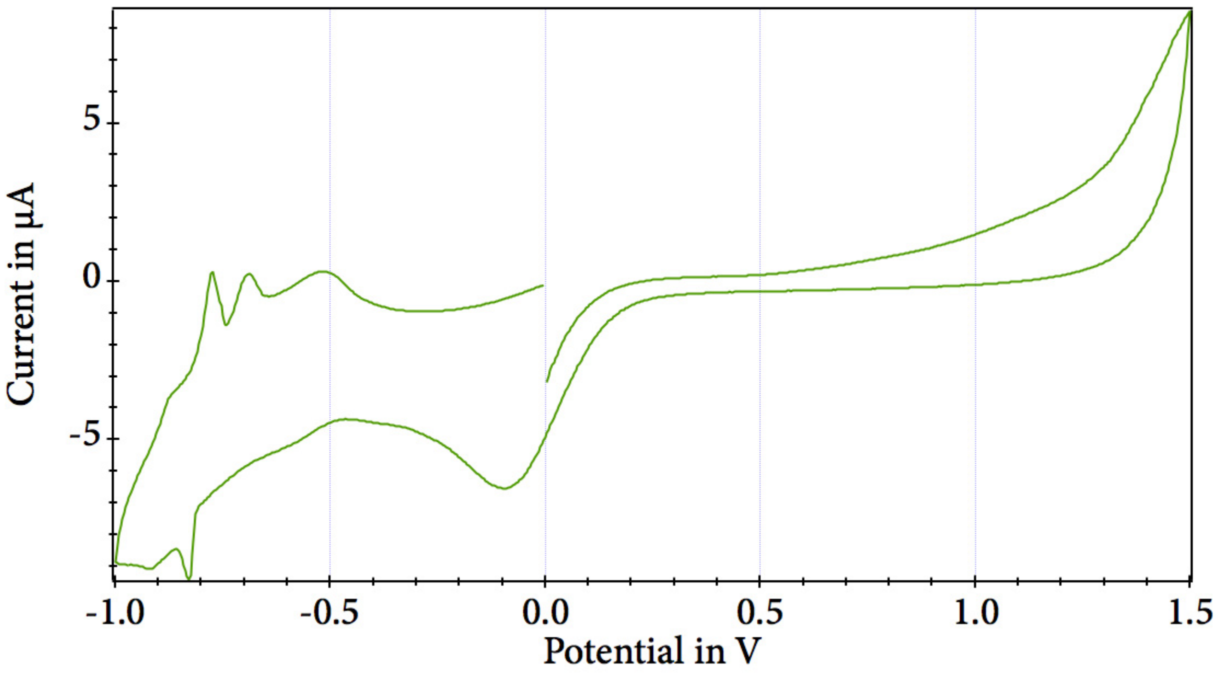

Figure S56. CV of compound **1** (5×10^{-4} M) in PB (50 mM, pH=7.0), referenced to Ag│AgCl.

10. ***Interaction with biomolecules.***

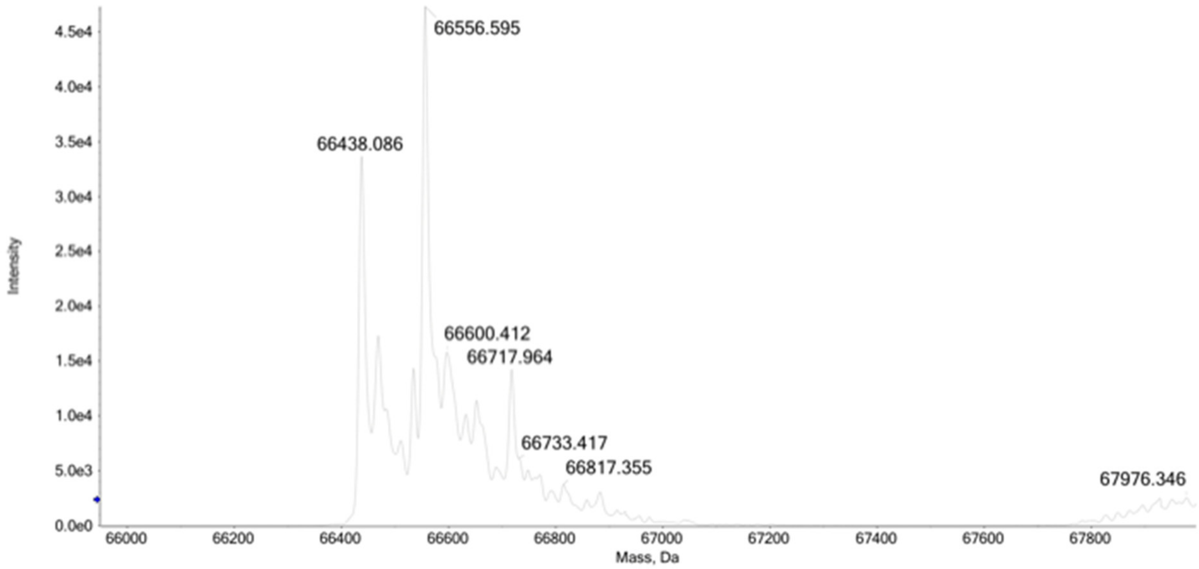

Figure S57. Deconvoluted ESI mass spectra of HSA (10^{-7} M) in Ammonium Acetate buffer (2×10^{-3} M, pH=6.8) with 0.1% of Formic Acid at 25 °C.
