# Supplementary material for: Synthesis, Characterization and Photoactivation Studies on the Novel Pt(IV)-Based [Pt(OCOCH3)3(phterpy)] Complex
Source: Int J Mol Sci. 2023 Jan 6;24(2):1106. doi: 10.3390/ijms24021106 (PMC9864011; doi:10.3390/ijms24021106)

checkCIF/PLATON report

Structure factors have been supplied for datablock(s) I

THIS REPORT IS FOR GUIDANCE ONLY. IF USED AS PART OF A REVIEW PROCEDURE FOR PUBLICATION, IT SHOULD NOT REPLACE THE EXPERTISE OF AN EXPERIENCED CRYSTALLOGRAPHIC REFEREE.

No syntax errors found. CIF dictionary Interpreting this report

Datablock: I

```
Bond precision:  C-C = 0.0057 A         Wavelength=0.71073

Cell:           a=9.7976(4)         b=10.6848(4)     c=11.3494(5)
                alpha=104.667(1)    beta=93.256(1)   gamma=93.129(1)
Temperature:    296 K

                 Calculated                  Reported
Volume           1144.62(8)                  1144.62(8)
Space group      P -1                        P -1
Hall group       -P 1                        -P 1
Moiety formula   C21 H15 I N3 Pt, C F3 O3 S  C21 H15 I N3 Pt, C F3 O3 S
Sum formula      C22 H15 F3 I N3 O3 Pt S     C22 H15 F3 I N3 O3 Pt S
Mr               780.41                      780.42
Dx,g cm-3        2.264                       2.264
Z                2                           2
Mu (mm-1)        7.624                       7.624
F000             732.0                       732.0
F000'            728.37
h,k,lmax         15,17,18                    15,17,18
Nref             10078                       9992
Tmin,Tmax        0.264,0.657                 0.296,0.747
Tmin'            0.025

Correction method= # Reported T Limits: Tmin=0.296 Tmax=0.747
AbsCorr = MULTI-SCAN

Data completeness= 0.991          Theta(max)= 34.998

R(reflections)= 0.0317( 8748)     wR2(reflections)= 0.0815( 9992)

S = 1.041                 Npar= 307
```

The following ALERTS were generated. Each ALERT has the format
test-name_ALERT_alert-type_alert-level.
Click on the hyperlinks for more details of the test.

Alert level B

```
PLAT972_ALERT_2_B Check Calcd Resid. Dens.  0.59A   From I1            -2.63 eA-3
```

Alert level C

```
PLAT244_ALERT_4_C Low    'Solvent' Ueq as Compared to Neighbors of         S1 Check
PLAT260_ALERT_2_C Large Average Ueq of Residue Including       S1      0.116 Check
PLAT910_ALERT_3_C Missing # of FCF Reflection(s) Below Theta(Min).        10 Note
PLAT911_ALERT_3_C Missing FCF Refl Between Thmin & STh/L=    0.600        54 Report
PLAT913_ALERT_3_C Missing # of Very Strong Reflections in FCF ....         8 Note
PLAT972_ALERT_2_C Check Calcd Resid. Dens.  0.66A   From I1            -2.34 eA-3
PLAT972_ALERT_2_C Check Calcd Resid. Dens.  0.39A   From I1            -2.26 eA-3
```

Alert level G

```
PLAT019_ALERT_1_G _diffrn_measured_fraction_theta_full/*_max < 1.0      0.994 Report
PLAT154_ALERT_1_G The s.u.'s on the Cell Angles are Equal ..(Note)      0.001 Degree
PLAT232_ALERT_2_G Hirshfeld Test Diff (M-X)  Pt1      --I1       .       13.7 s.u.
PLAT244_ALERT_4_G Low    'Solvent' Ueq as Compared to Neighbors of        C22 Check
PLAT434_ALERT_2_G Short Inter HL..HL Contact F1       ..F1               2.71 Ang.
                                                 -x,2-y,-z  =      2_575 Check
PLAT883_ALERT_1_G No Info/Value for _atom_sites_solution_primary .     Please Do !
PLAT912_ALERT_4_G Missing # of FCF Reflections Above STh/L=  0.600        23 Note
PLAT933_ALERT_2_G Number of OMIT Records in Embedded .res File ...        15 Note
PLAT978_ALERT_2_G Number C-C Bonds with Positive Residual Density.         3 Info
PLAT992_ALERT_5_G Repd & Actual _reflns_number_gt Values Differ by         2 Check
```

```
   0 ALERT level A = Most likely a serious problem - resolve or explain
   1 ALERT level B = A potentially serious problem, consider carefully
   7 ALERT level C = Check. Ensure it is not caused by an omission or oversight
  10 ALERT level G = General information/check it is not something unexpected

   3 ALERT type 1 CIF construction/syntax error, inconsistent or missing data
   8 ALERT type 2 Indicator that the structure model may be wrong or deficient
   3 ALERT type 3 Indicator that the structure quality may be low
   3 ALERT type 4 Improvement, methodology, query or suggestion
   1 ALERT type 5 Informative message, check
```

checkCIF publication errors

Alert level A

```
PUBL004_ALERT_1_A  The contact author's name and address are missing,
            _publ_contact_author_name and _publ_contact_author_address.
PUBL005_ALERT_1_A  _publ_contact_author_email, _publ_contact_author_fax and
            _publ_contact_author_phone are all missing.
            At least one of these should be present.
PUBL006_ALERT_1_A  _publ_requested_journal is missing
            e.g. 'Acta Crystallographica Section C'
PUBL008_ALERT_1_A  _publ_section_title is missing. Title of paper.
PUBL009_ALERT_1_A  _publ_author_name is missing. List of author(s) name(s).
PUBL010_ALERT_1_A  _publ_author_address is missing. Author(s) address(es).
PUBL012_ALERT_1_A  _publ_section_abstract is missing.
            Abstract of paper in English.
```

Alert level G

```
PUBL017_ALERT_1_G The _publ_section_references section is missing or
            empty.
```

```
   7 ALERT level A = Data missing that is essential or data in wrong format
   1 ALERT level G = General alerts. Data that may be required is missing
```

Publication of your CIF

You should attempt to resolve as many as possible of the alerts in all categories. Often the minor alerts point to easily fixed oversights, errors and omissions in your CIF or refinement strategy, so attention to these fine details can be worthwhile. In order to resolve some of the more serious problems it may be necessary to carry out additional measurements or structure refinements. However, the nature of your study may justify the reported deviations from journal submission requirements and the more serious of these should be commented upon in the discussion or experimental section of a paper or in the "special_details" fields of the CIF. *checkCIF* was carefully designed to identify outliers and unusual parameters, but every test has its limitations and alerts that are not important in a particular case may appear. Conversely, the absence of alerts does not guarantee there are no aspects of the results needing attention. It is up to the individual to critically assess their own results and, if necessary, seek expert advice.

If level A alerts remain, which you believe to be justified deviations, and you intend to submit this CIF for publication in a journal, you should additionally insert an explanation in your CIF using the Validation Reply Form (VRF) below. This will allow your explanation to be considered as part of the review process.

Validation response form

Please find below a validation response form (VRF) that can be filled in and pasted into your CIF.

```
# start Validation Reply Form
_vrf_PUBL004_GLOBAL
;
PROBLEM: The contact author’s name and address are missing,
RESPONSE: ...
;
_vrf_PUBL005_GLOBAL
;
PROBLEM: _publ_contact_author_email, _publ_contact_author_fax and
RESPONSE: ...
;
_vrf_PUBL006_GLOBAL
;
PROBLEM: _publ_requested_journal is missing
RESPONSE: ...
;
_vrf_PUBL008_GLOBAL
;
PROBLEM: _publ_section_title is missing. Title of paper.
RESPONSE: ...
;
_vrf_PUBL009_GLOBAL
;
PROBLEM: _publ_author_name is missing. List of author(s) name(s).
RESPONSE: ...
;
```

```
_vrf_PUBL010_GLOBAL
;
PROBLEM: _publ_author_address is missing. Author(s) address(es).
RESPONSE: ...
;
_vrf_PUBL012_GLOBAL
;
PROBLEM: _publ_section_abstract is missing.
RESPONSE: ...
;
# end Validation Reply Form
```

If you wish to submit your CIF for publication in Acta Crystallographica Section C or E, you should upload your CIF via the web. If you wish to submit your CIF for publication in IUCrData you should upload your CIF via the web. If your CIF is to form part of a submission to another IUCr journal, you will be asked, either during electronic submission or by the Co-editor handling your paper, to upload your CIF via our web site.

PLATON version of 22/04/2020; check.def file version of 09/03/2020

Datablock I - ellipsoid plot

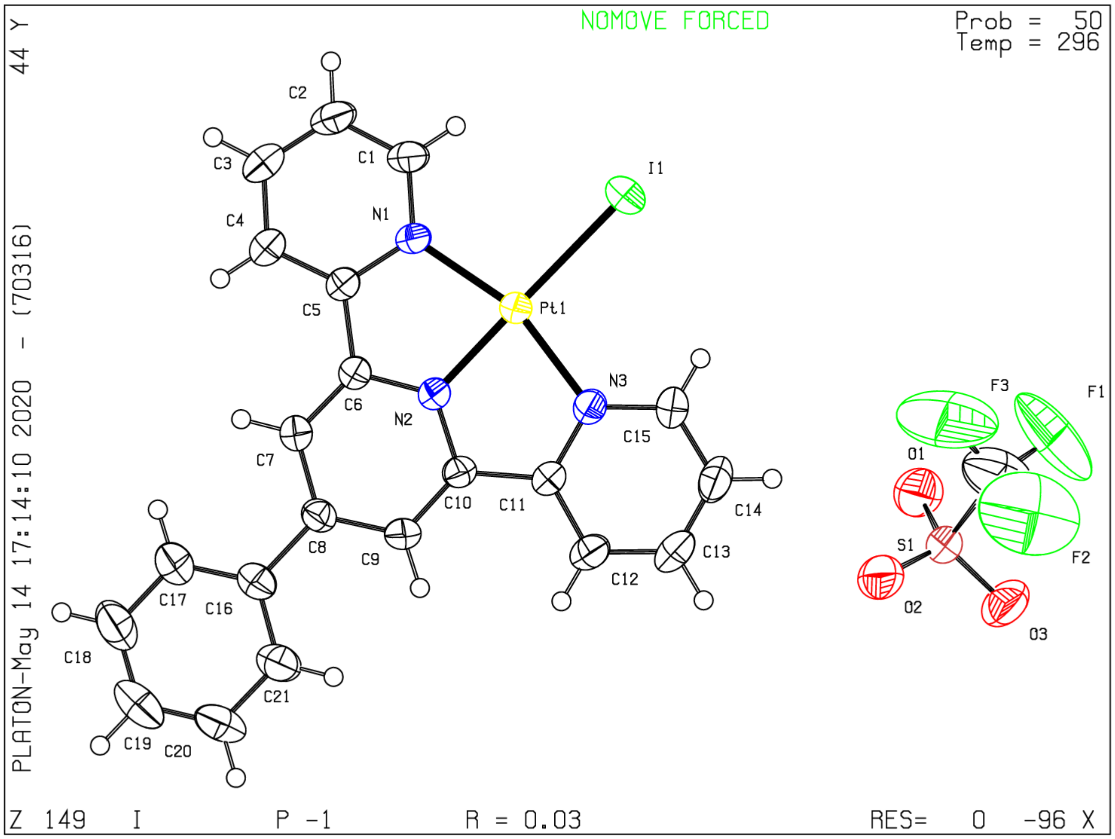

Supplement: Supplementary file 1 [file ijms-24-01106-s001.zip › checkcif_2.pdf]
